# Use of Microwave Dielectric Spectroscopy for the *In Actu* Assessment of Frustrated Lewis Pair Encounter Complexes

**DOI:** 10.1021/jacs.4c02736

**Published:** 2024-07-16

**Authors:** Cihang Yu, Jamie A. Leitch, Lukas Gierlichs, Sampurna Das, Adrian Porch, Rebecca L. Melen, Duncan L. Browne

**Affiliations:** † Department of Pharmaceutical and Biological Chemistry, University College London, School of Pharmacy, 29-39 Brunswick Square, Bloomsbury, London W1CN 1AX, U.K.; ‡ Cardiff Catalysis Institute, School of Chemistry, Cardiff University, Translational Research Hub, Maindy Road, Cathays, Cardiff, Cymru/Wales CF24 4HQ, U.K.; § Centre for High Frequency Engineering, School of Engineering, Cardiff University, Queen’s Buildings, Newport Road, Cardiff, Cardiff CF24 3AA, U.K.

## Abstract

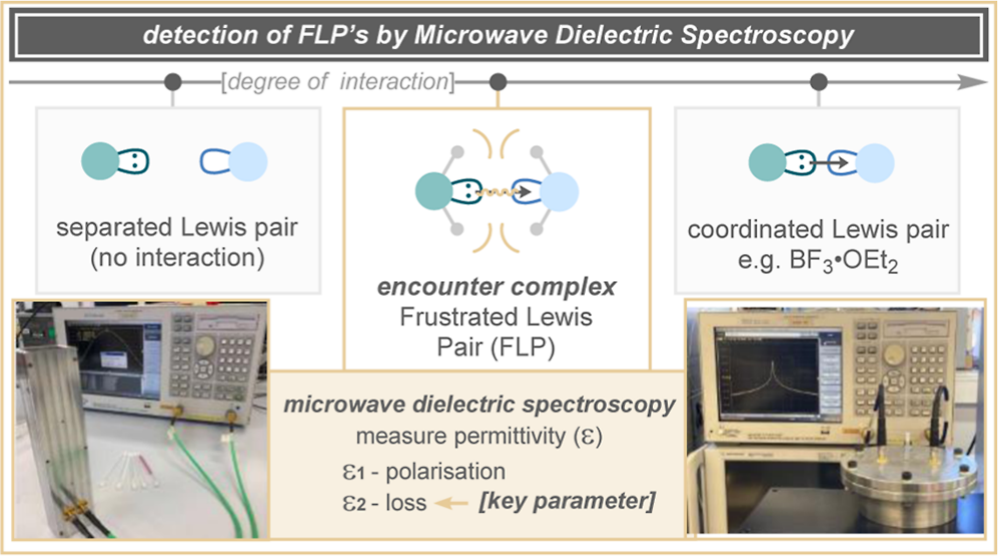

Frustrated Lewis pairs (FLPs) offer an important and promising paradigm for main group catalysis. Reported here is the use of microwave dielectric spectroscopy for the *in actu* detection of FLP encounter complexes. This technique focuses on the room-temperature measurement of the loss component of microwave permittivity (ε_2_) over the bandwidth from 0.5 to 6.8 GHz. The microwave loss measured for a Lewis pair in a toluene host solution is compared with the losses of the individual components when measured separately, and the difference in loss Δε_2_ is used to characterize the electrostatic interaction between the pair. The Δε_2_ value shows a direct correlation with an ability for the FLP encounter complex to split hydrogen gas and abstract hydrogen from γ-terpinene and has led to the identification of a novel FLP encounter complex, tris-pentafluorophenyl borane-eucalyptol pairing.

## Introduction

**Scheme 1:**
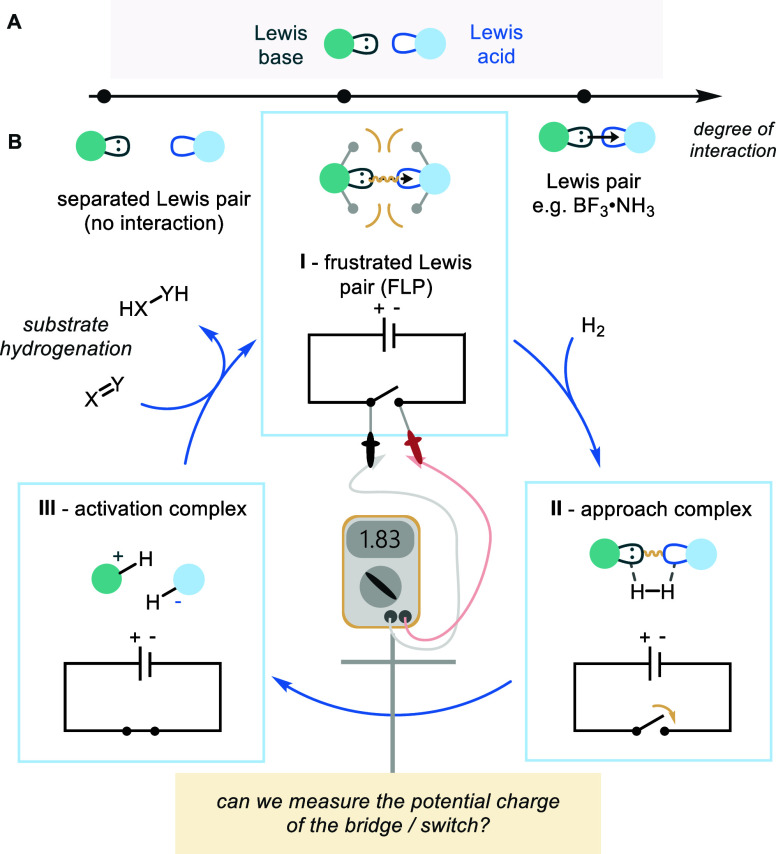
Summary of FLPs in Catalysis (A) degree of interation between Lewis pair components; (B) is there an observable change in potential across the FLP during catalysis?.

Frustrated Lewis pairs (FLPs) have great potential as metal-free catalysts^1a,1b^ for the activation of small molecules such as hydrogen, carbon dioxide, nitrogen oxides, sulfur dioxide, and olefins.^2a−2h^ This has led to intensive research into their catalytic potential and properties over the last two decades. For catalytic applications, the nature of the FLP determines the reaction mechanism for the activation of small molecules. The mechanism for small molecule activation, e.g., hydrogen, could involve either preactivation of the small molecule by its association with the Lewis acid (LA) or Lewis base (LB) with concomitant splitting from the other Lewis partner or by the preformation of a LA–LB reactive encounter complex that can then split a small molecule. The strength and steric effects of interactions within Lewis pair components and substrate molecules, through Coulomb forces or molecular orbital overlap, play crucial roles in determining their catalytic reactivity. In an archetypal catalytical cycle of an encounter complex, the electric field model of a FLP considers the pair to be a charged capacitor (Scheme 1B(I)). Upon the approach of a small molecule, the electric potential energy stored by the FLP can be released, leading to the cleavage of the small molecule and the formation of an ionic complex (Scheme 1B(II + III)).^3a−3d^ In the case of hydrogen activation, subsequent transfer of the hydride and proton to an acceptor substrate (such as an alkene, imine, or carbonyl species) will allow the hydrogenation of the substrate and restoration of the frustration energy locked into the FLP-reactive encounter complex.

Within this context, the fine details of what makes a good FLP remain elusive, and FLP design and optimization are still challenging and largely empirical. The structures and electronic profiles of FLPs can be observed by solid-state NMR and X-ray diffraction.^4a−4c^ The reactivities of FLPs are currently estimated by the Lewis acidity and Lewis basicity of each individual component or the activation energy calculated using DFT.^5a−5i^

A number of different approaches to characterize FLPs have been attempted and reported in the literature. By NMR spectroscopy, there is no difference in ^1^H, ^11^B, ^19^F, and ^31^P spectra between classical FLPs such as P(^*t*^Bu)_3_·B(C_6_F_5_)_3_ and P(mes)_3_·B(C_6_F_5_)_3_ and between those of the individual components.^2b,4b,6a^ The use of ^1^H–^19^F HOESY and solid-state NMR techniques is more fruitful and was reported for measuring the intermolecular interaction between FLP components.^6a,6b^ Further NMR studies facilitated with neutron scattering and supramolecular association analysis have been used to study the presence of weakly associated encounter complexes, specifically P^*t*^Bu_3_·B(C_6_F_5_)_3_ and lutidine·B(C_6_F_5_)_3_ Lewis pairs.^6d−6f^ Further to this, vibrational spectroscopy^7a,7b^ and UV spectroscopy^7c^ have been used to investigate the encounter complexes of FLPs by the teams of Ando and Jupp, respectively.

It occurred to us that given the strong electrostatic interactions between the constituent molecules, it may be possible to directly study (*in actu*) the dynamic interactions between the Lewis pair partners constituting an encounter complex using microwave dielectric spectroscopy (MDS), in which the electric dipole moments thus formed are coupled to the microwave electric field. MDS operates at low power levels (<1 mW, so there is no heating) and is noninvasive, highly precise, and very fast; it could offer the potential of measuring the degree of frustration of the Lewis pairing. Herein, we report our findings on the use of bespoke MDS instrumentation designed specifically to target FLP samples within a suitable host solvent (toluene, which has a relatively small microwave loss).

## Results and Discussion

### Microwave Dielectric Spectroscopy

**Scheme 2:**
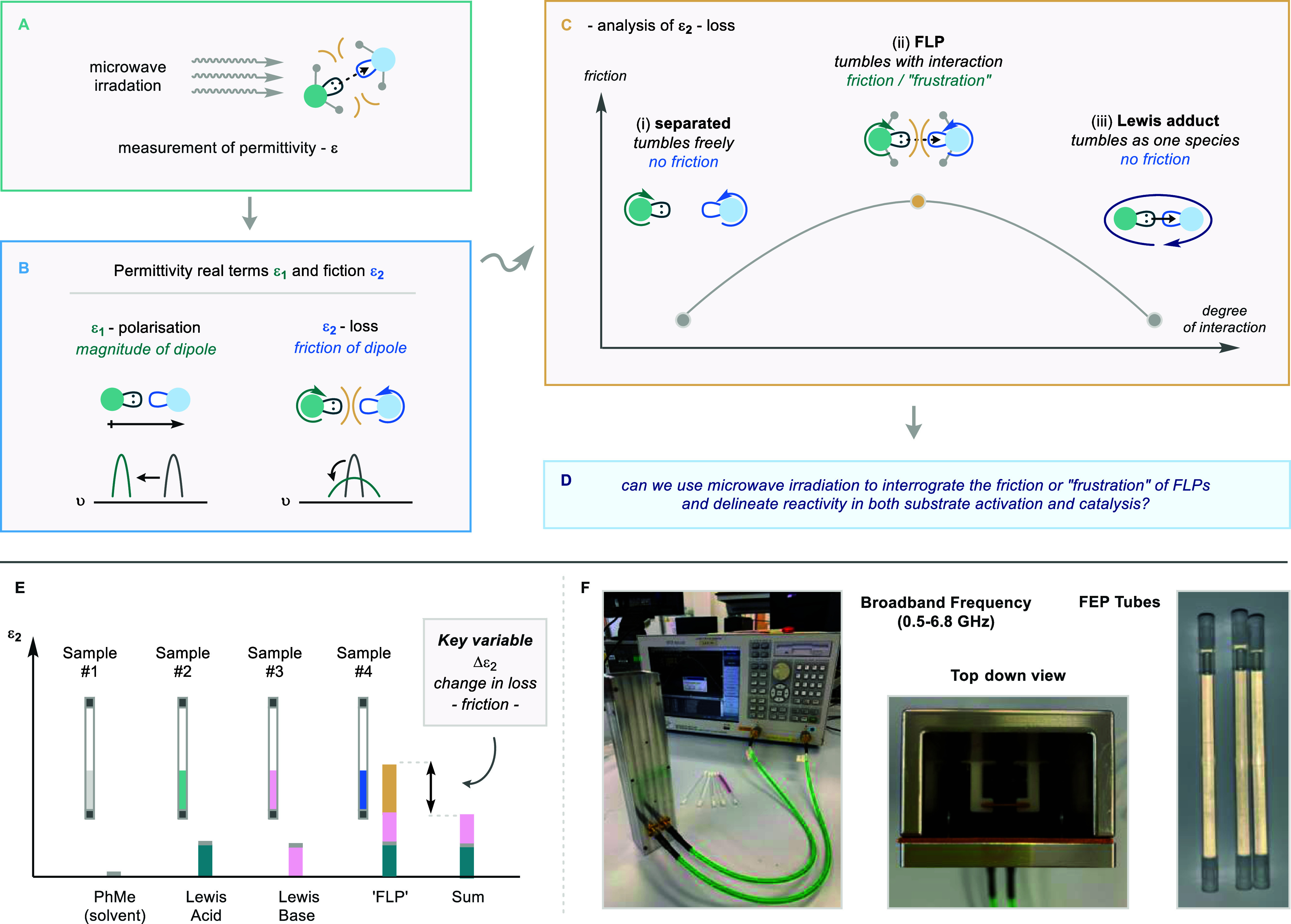
(A) Illustration of Microwave Spectroscopy; (B) Two Permittivity Factors in Microwave Spectroscopy: Polarization, Observed as a Shift in Peak Position, and Loss, Observed as a Broadening or Sharpening in Peak Shape; (C) Expected Relationship between Lewis Acid–Base Pairings with Intermolecular Friction Force; (E) Proposed Methodology for Measuring the Permittivity of Lewis Acid–Base Pairings; and (F) MDS Equipment

The permittivity (ε) of a material is quantified by its electric polarization in response to an applied electric field. This concept is most familiar to synthetic chemists in the measurement and utility of dielectric constants for solvent selection, especially for those being used in microwave reactors. For polar molecules, the largest contribution to ε at microwave frequencies is due to the effects of molecular alignment and rotation (Scheme 2A). The polarization of the sample is quantified in terms of the dielectric constant ε (more properly ε_1_), which is a measure of the polarization and hence the stored electric potential energy per unit electric field (Scheme 2B). However, coupled with the molecular motion is the energy loss due to the dipole–dipole relaxation effect, quantified through the loss term ε_2_; this is most easily thought of as being the frictional energy loss between rotating molecules that are in close proximity with each other (Scheme 2B). MDS involves the simultaneous measurement of both polarization ε_1_ and loss ε_2_ terms over a typical bandwidth of 0.1–10.0 GHz.^8a−8c^

We use microwave resonators for MDS owing to their very high resolution for measurement of loss (ε_2_) in particular and their ability to measure small sample volumes in sealed tubes (since the FLPs studied are highly air sensitive). However, the measurement is restricted to discrete resonant frequencies, so we use the overlapping spectra of a cylindrical cavity resonator (CCR, operating at 2.5, 4.6, 5.7, and 6.8 GHz), a parallel plate resonator (PPR, at 1.0, 2.0, 3.0, and 4.0 GHz), and a hairpin variant of the PPR (known as the HPR) that reduces the frequency further to 0.5 GHz (with harmonics at 1.5, 2.5, and 3.5 GHz).^9a−9c^

All measurements were taken at room temperature (nominally 20 °C) with each measurement taking 5 seconds. Within the context of FLP complexes, the analysis of loss (ε_2_) could provide valuable information about the levels of frustration between a Lewis acid and a Lewis base. Separated Lewis complexes (i, Scheme 2C) will rotate freely with little friction, whereas full Lewis pairs/adducts will rotate as one species, also with little friction (iii, Scheme 2C).

However, we expect that FLP encounter complexes, within which no formal dative bond exists, will rotate independently in very close proximity, causing a high degree of frictional losses, which should be observable as an enhancement of ε_2_ (ii, Scheme 2C). Such strong interactions at the boundary of a Lewis acid and base could be attributed to a reactive encounter complex.^7c,10^ We anticipate much smaller effects on the polarization ε_1_ since this quantifies the total electric dipole moment of the complexes.

Our focus on the measurement of ε_2_ justifies our use of resonant MDS and, since the relaxation frequency of such a physically large FLP complex is likely to be well below 1.0 GHz, we expect the enhancement in ε_2_ due to friction to grow as the frequency is reduced. Hence, the need for a MDS applicator such as the PPR and its HPR variant, which have low fundamental frequencies (set by their lengths, to which frequency is inversely proportional) but which still allow measurement of small sample volumes, in our case around 0.3 mL, within plugged fluorinated ethylene propylene (FEP) sample tubes. We chose FEP owing to its low chemical reactivity, very low microwave loss, and closely matched ε_1_ value to toluene (2.0 and 2.4, respectively).

### Sample Concentration

**Figure 1:**
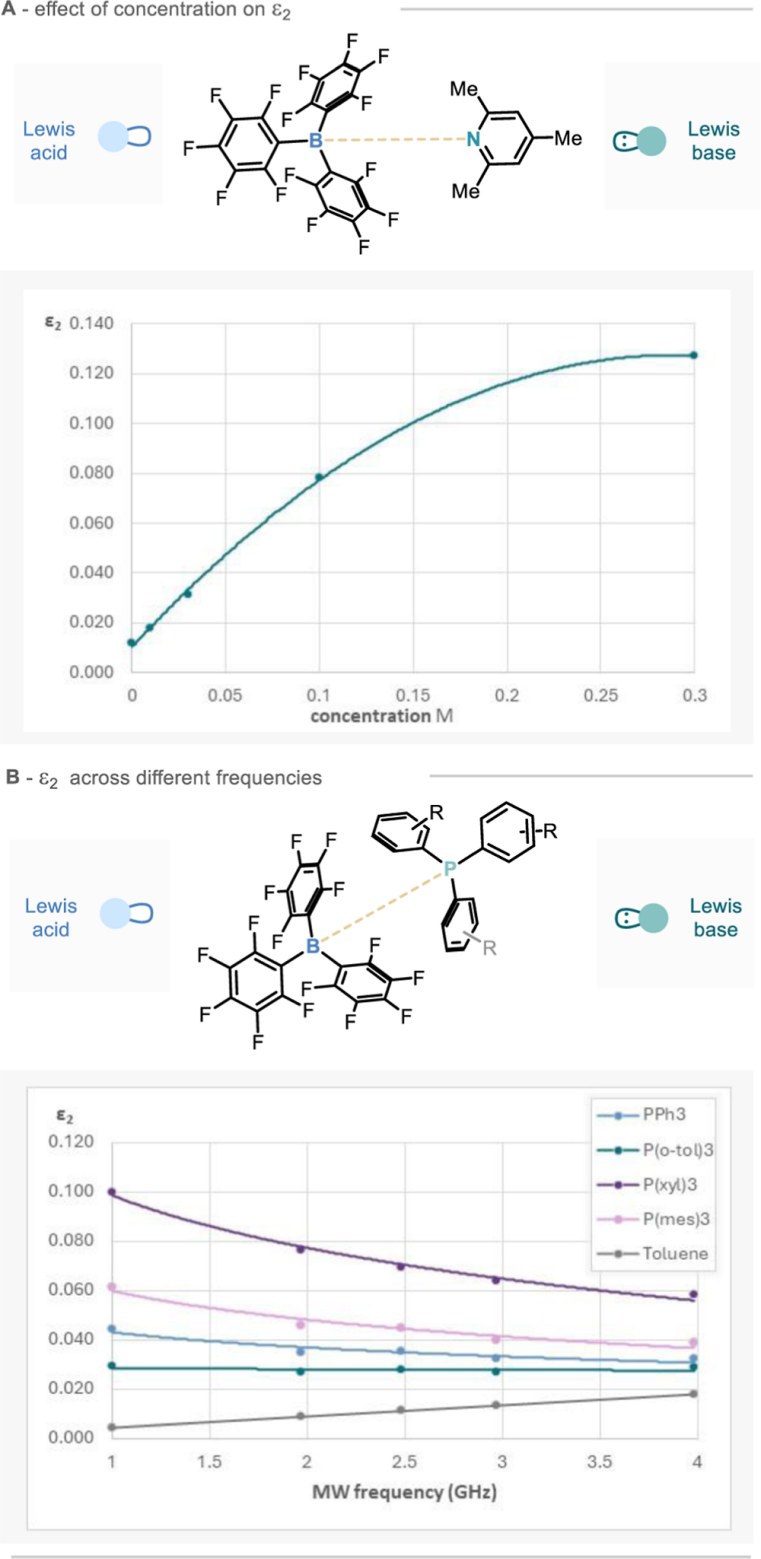
(a) Effect of sample concentration on loss (ε_2_) at 1.0 GHz for tris-pentafluorophenyl borane-collidine. (b) Dependence of loss ε_2_ on frequency from 1.0 to 4.0 GHz for different P–B pairs; standard errors in complex permittivity were evaluated from three independent sample measurements and are less than ±1%.

The ε_1_ and ε_2_ values of the empty FEP sample tube, toluene, Lewis acids (in toluene at 0.1 M), Lewis bases (in toluene at 0.1 M), and Lewis pairs (in toluene, each at 0.1 M) were measured across a range of microwave frequencies (0.5–6.8 GHz). Initial measurements explored the influence of sample concentration on permittivity change. As shown in Figure 1A, measurement of B(C_6_F_5_)_3_, tris-pentafluorophenyl borane, (pentaF, LA3)-collidine at concentrations of 0.1 M shows a significant enhancement in microwave loss over pure toluene and is a common concentration used for FLPs in catalytic reaction processes. Nonetheless, it is also possible to obtain very precise MDS loss measurements (to a standard error of <±1%) at concentrations below 0.01 M.

To optimize the frequency range used for our MDS, a set of measurements were carried out with triaryl phosphines at 1.0, 2.0, 2.5, 3.0, and 4.0 GHz using the PPR and one mode of the CCR. The changes in loss, Δε_2_, increase as the frequency is reduced, as would be expected for a simple Debye-type relaxation model for the complex, as shown in Figure 1B, and leads us to conduct MDS measurements at 1.0 GHz so as to maximize the differentiation in response to various Lewis pairings.

### Loss Enhancement Measurements (Δε_2_)

We performed separate MDS measurements of the loss of the FLP complex ε_2,FLP_, the Lewis acid ε_2,LA_, and the Lewis base ε_2,LB_ (all in toluene), and we then calculate the loss enhancement term Δε_2_ using eq 1, taking care to extract the toluene background from each component (summarized visually in Scheme 2E).
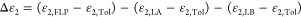
1

Since microwave losses are additive, we expect Δε_2_ to be close to zero for acids and bases that do not interact when mixed, as any loss is associated with dipole interactions between the individual Lewis acid and Lewis base molecules with the surrounding toluene, which will be largely the same as when they are measured in toluene in isolation. However, if frictional loss is present within the FLP complex, then we expect Δε_2_ to be large, increasing at lower frequencies. Hence, we propose that the loss enhancement is a direct measure of the frustration of the FLP complex (Scheme 2E) and can be modeled as a Debye-type relaxation process with a strong intermolecular attraction between the dipoles of large molecules, leading to a relaxation frequency below 1.0 GHz. Our initial measurements of broadband MDS using a coaxial reflectance probe imply a FLP relaxation frequency around 0.1 GHz, though these measurements are invasive and do not offer the high resolution afforded by the resonant MDS as described here.

**Figure 2:**
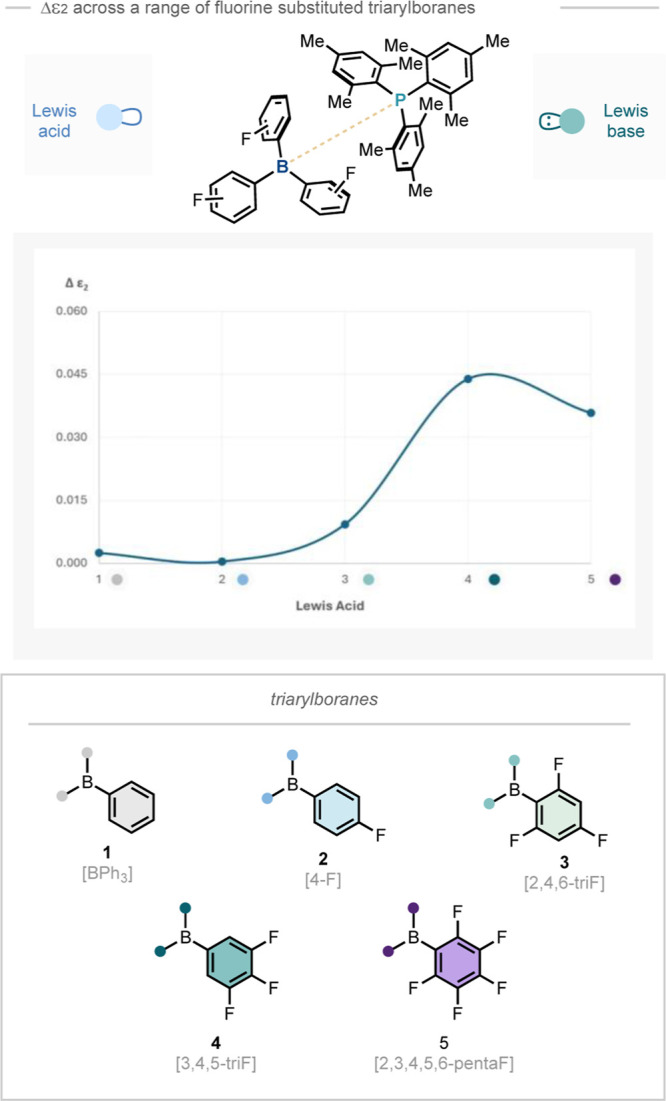
Loss ε_2_ of a range of Lewis acids with P(mes)_3_.

Proof-of-concept experiments were carried out by measuring the Δε_2_ values of FLPs with different Lewis acid components paired with one Lewis base known to be sterically bulky. This method is similar to previous studies on individual FLP components.^5^ In this study, Lewis base P(mes)_3_ (LB3) was selected and measured with a range of borane Lewis acids with established Lewis acidity. An enhanced Δε_2_ value was obtained, which had a strong positive correlation with increased Lewis acidity (Figure 2).^11a,11b^ Mindful of the necessity for dry samples and the potential impact of water on the MDS measurement, we conducted a control experiment. Samples of LA, LB, and FLP were prepared and measured (*t* = 0), the cap of the three sample tubes was then removed, and the samples were exposed to air atmosphere for 2 days with further MDS measurements on day 1 and day 2 of these “open” samples. The data, provided in the Supporting Information document, shows that the MDS response of the LA is (unsurprisingly) dramatically impacted by the air atmosphere, the FLP sample mirrors the LA response, whereas the LB remains largely unchanged. This experiment provides some level of confidence in the absence of water in our samples.

### Screening of Lewis Pairs by MDS

To assess the suitability of MDS for screening of Lewis acids and bases,^1a,1b,12a−12d^ the loss enhancements (Δε_2_) of Lewis pairs were measured and then calculated using eq 1 and summarized in Table 1a. Note that BPh_3_ does not show a strong enhancement (so, we infer, no reactive encounter complex) with any of the Lewis bases tested. This is expected, as without the electron-deficient aryl ring, the borane in BPh_3_ is a very weak Lewis acid.^14^ Most 2,4,6-triF (LA2) Lewis acid combinations with Lewis bases show no loss enhancement (Δε_2_), with the exception of a weak interaction with the electron-rich P(xyl)_3_ and P(mes)_3_ (LB2 and LB3) bases. Large Δε_2_ values were found between the strong Lewis acid (LA3) and most of the phosphine Lewis bases tested. The general tendency of B(C_6_F_5_)_3_, LA3 to give a larger Δε_2_ value with a Lewis base than BPh_3_ or 2,4,6-triF is consistent with the Lewis acidity measurement by the Gutmann–Beckett or Childs Methods.^5g,5h^ Pairs formed between borane Lewis acids (specifically LA3) and ethereal Lewis bases also demonstrate Δε_2_ values of magnitude similar to those of the phosphine Lewis bases. However, nitrogen Lewis bases (such as pyridine and its methyl-substituted derivatives) currently prove elusive for accurate measurement by this MDS approach. We attribute this, in part, to the propensity of 2-methylated pyridine systems to exhibit tautomeric-type behavior in the presence of borane Lewis acids.^13a,13b^ Cationic and organometallic Lewis pairs were not studied at this stage.

**Table 1:**
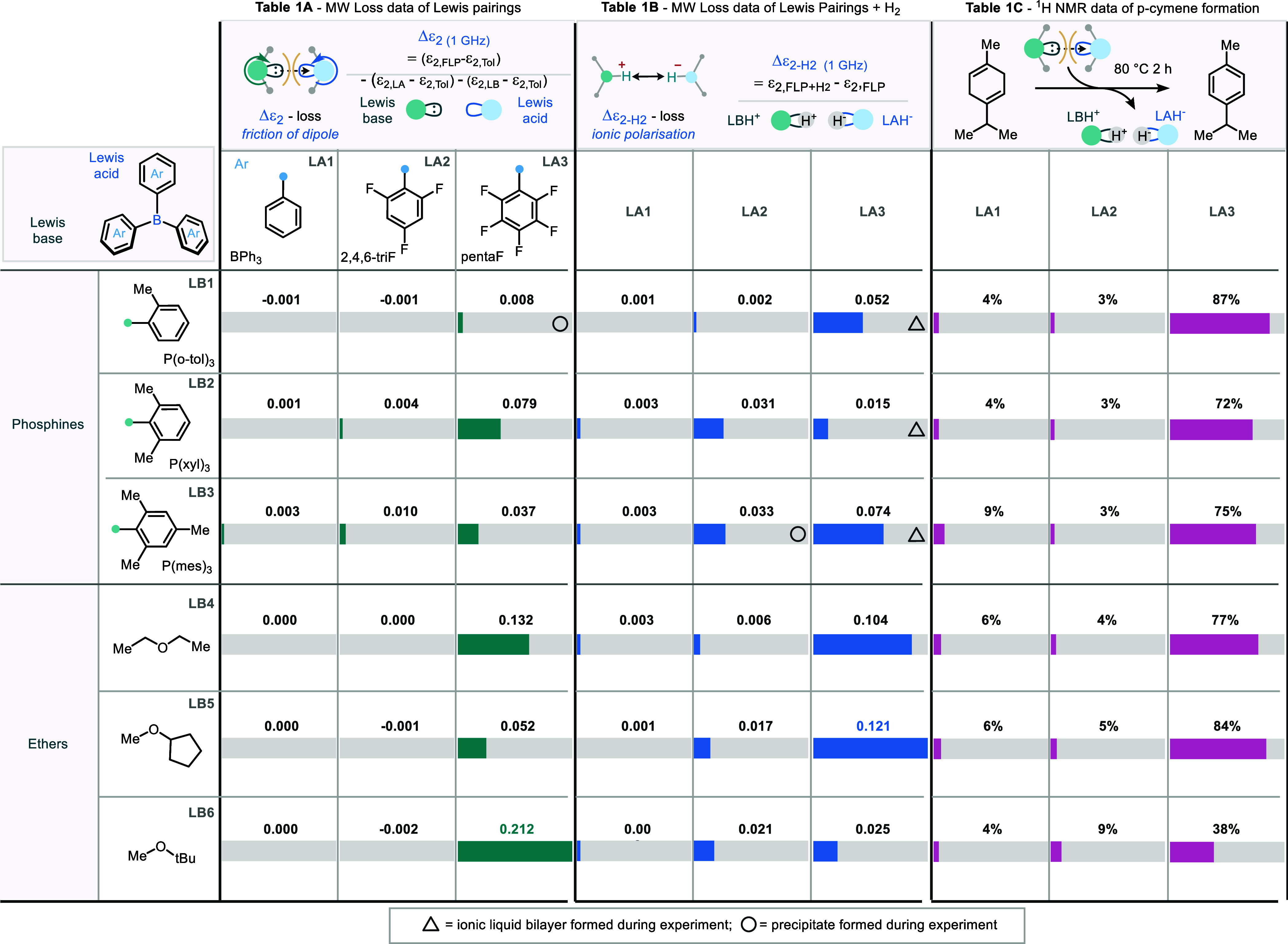
(A) Response Bars of the Loss Enhancement, Δε_2_ of Selected Lewis Pairs, Colored Portion as a Percentage of 0.212; (B) Response Bars of Δε_2_ for the H_2_ Response of Selected Lewis Pairs, Colored Portion as a Percentage of 0.121; and (C) Response Bars of Conversion of γ-Terpinene by Selected Lewis Pairs, Colored Portion as a Percentage^a^

a Triangle denotes the ionic liquid bilayer formed in the sample; circle denotes the precipitation formed in the sample.

### Loss Change during Hydrogen Splitting Δε_2–H_2__

Having demonstrated the ability to measure the Δε_2_ values of FLP encounter complexes with high accuracy and discrimination between different combinations of FLP components, we next sought to measure the ability of each combination to split hydrogen using the MDS technique. To collect the required data, the combination of Lewis pairs measured in Table 1a was charged with hydrogen and the change in the loss of Lewis pairs before and after hydrogenation was recorded as Δε_2–H_2__, defined in eq 2. The absolute loss values before and after hydrogenation contain the same (small) background loss of the toluene host, which is subtracted in eq 2. In separate experiments, we have shown that hydrogenation has no effect on the loss of pure toluene.
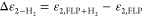
2

**Scheme 3:**
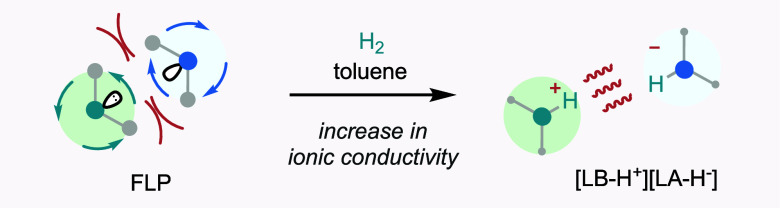
Activation of Hydrogen by a FLP Results in an Enhanced ε_2_ Value Associated with Ionic Conductivity

Prior to the experiment, it was reasoned that the values of Δε_2–H_2__ during the hydrogenation process are likely to be the result of a second type of microwave loss mechanism driven by the microwave electric field that of ionic conduction by the protonated and hydride FLP species formed as products of the reaction (Scheme 3).

Values of Δε_2–H_2__ are summarized in Table 1b. Reactive FLPs are most likely to transfer into a protonated Lewis base [LB-H^+^] and hydride-bound Lewis acid [LA-H^–^].^1a,1b,2a,2b^ The hydrogenated species [LB-H^+^][LA-H^–^] are ionic and therefore will generally increase the ε_2_ value. Since the only factor expected to increase microwave loss during the process of hydrogen splitting is the generation of ion pairs, we propose that changes in Δε_2–H_2__ can be used to determine the progress and success of the dihydrogen activation reaction. We expect Δε_2–H_2__ to be proportional to σ/ω, where σ is the (dc) conductivity of the solution (approximately proportional to the ionic concentration) and ω = 2π*f* is the (angular) frequency. Hence, the conduction losses increase with decreasing frequency (as observed experimentally after hydrogenation), and the sensitivity of MDS for loss measurements is increased at lower frequency (in our case, at 1 GHz and below). Here, we are effectively using MDS as a noninvasive, noncontacting measurement of the electrical conductivity of the solutions. The response bars show that Lewis pairs with Δε_2_ values >0.004 are associated with competent hydrogen splitting, delivering Δε_2–H_2__ values >0.031 or a phase separation (*vide infra*). It is also noted, however, that 2,4,6-triF, in combination with either of the 3 ethereal Lewis bases, affords a negligible Δε_2_ response, but Δε_2–H_2__ values ranging from 0.006 to 0.021. This data suggests that either there is hydrogen splitting activity that was not predictable by the Δε_2_ measurement method described here or that the splitting is occurring via a mechanism that does not involve a discrete reactive encounter complex (i.e., likely via a preassociation of LA or LB with H_2_).^5a,15a−15d^

During the running of these hydrogenation experiments, it was noted that a solid precipitate is formed on the introduction of hydrogen gas to the pairing of LA2 (2,4,6-triF) with LB2 (P(xyl)_3_), which we attribute to the corresponding [LB-H^+^][LA-H^–^] ionic species (Table 1b, marked with a circle). Such a precipitate has a much lower microwave loss compared to the frictional or conduction losses operating on solutions, so the absolute values of these measurements should be discounted. Similarly, in the case of the interaction of LA3 with LB1, 2, and 3, the hydrogenated Lewis pairs form an ionic liquid which separates into a nonmiscible secondary phase (Table 1b, marked with a triangle). A very large increase in ε_2_ was observed upon the formation of this emulsion, and very large values were also obtained with the isolated pure ionic liquids. This observation is consistent with large ε_2_ values previously measured with MDS for ionic liquids.^16^ To summarize this behavior, while both the formation of precipitates and ionic liquids lead to inaccurate absolute values by MDS, the very fact that these materials form demonstrates the hydrogen splitting reactivity of these respective Lewis acid and Lewis base pairings.

Notably, strong Δε_2–H_2__ values are also observable with ethereal Lewis bases, LB4, 5, and 6 at room temperature. Both ethereal and ketone oxygens have been reported as effective Lewis bases in FLPs for reversible hydrogenation and transfer hydrogenation reactions.^12b,17a−17c^

### Correlation of MDS-Measured Δε_2_ Values with ^1^H NMR Hydrogen Extraction Reactivity

**Scheme 4:**
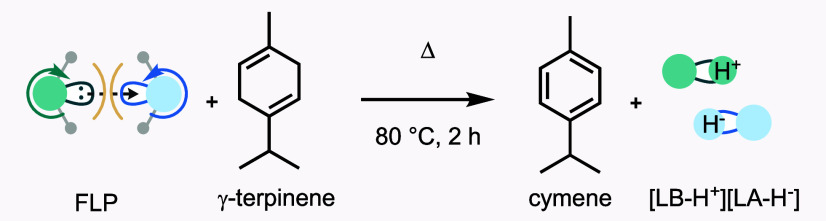
Hydrogen Extraction from γ-Terpinene Forms Dihydrogen Lewis Pairs

Having assessed MDS to measure both Lewis acid/base combinations and their ability to split hydrogen, we sought further verification of the observed trends. Mindful of the propensity for some of these combinations to afford precipitates and ionic liquids, we turned our attention to the process of hydrogen transfer from a model hydrogen donor γ-terpinene and ^1^H NMR spectroscopy (Table 1c and Scheme 4). Such a process commonly appears in transfer hydrogenation methods mediated by FLPs.^18a,18b^ In such an experiment, reactive Lewis pairs can extract hydrogen from a dihydrogen surrogate such as γ-terpinene, driven by the aromatization of the 1,4-cyclodiene ring, of which both species (starting material and product) can be monitored by ^1^H NMR spectroscopy. The response bars of the hydrogen extraction process demonstrate that Lewis pairs with values of Δε_2_ > 0.037 are also associated with competent hydrogen extraction reactivity (>38%, Table 1).

Taken collectively, the data presented in Table 1 demonstrate that MDS is an effective technique for gauging if a Lewis acid/base combination is competent for splitting or abstracting hydrogen, a characteristic property of a frustrated Lewis pair. However, the current capability does not assess if the combination is a competent catalyst (i.e., no on/off or transfer rates for hydrogen have been measured).

### Using MDS to Discover and Develop New FLPs

To demonstrate the potential of MDS to find new, active FLP combinations, a novel Lewis base (eucalyptol) was examined in combination with the three boranes by MDS and ^1^H NMR spectroscopy (Table 2). Notably, the ether oxygen of eucalyptol is sterically hindered with a bridged bond angle (C–O–C) of 115° rather than a typical monocyclic (C–O–C) bond angle of 110°.^19^ Additionally, the two electron-rich tertiary carbon atoms attached to eucalyptol contribute to an increased electron density on the oxygen lone pairs. The data shows that eucalyptol has a moderate Δε_2_ value with LA3 (0.027), congruent with those entries in Table 1a which led to activity for hydrogen splitting and hydrogen abstraction. The MDS data for the eucalyptol–LA3 pair in the presence of hydrogen indeed give rise to a Δε_2–H_2__ value of 0.152, signifying the splitting of hydrogen. Moreover, treatment of the eucalyptol–LA3 pair with γ-terpinene led to the formation of 78% of cymene, demonstrating the ability of the pairing to competently abstract hydrogen (on par with the phosphine–LA3 pairings in Table 1c).

**Table 2:**
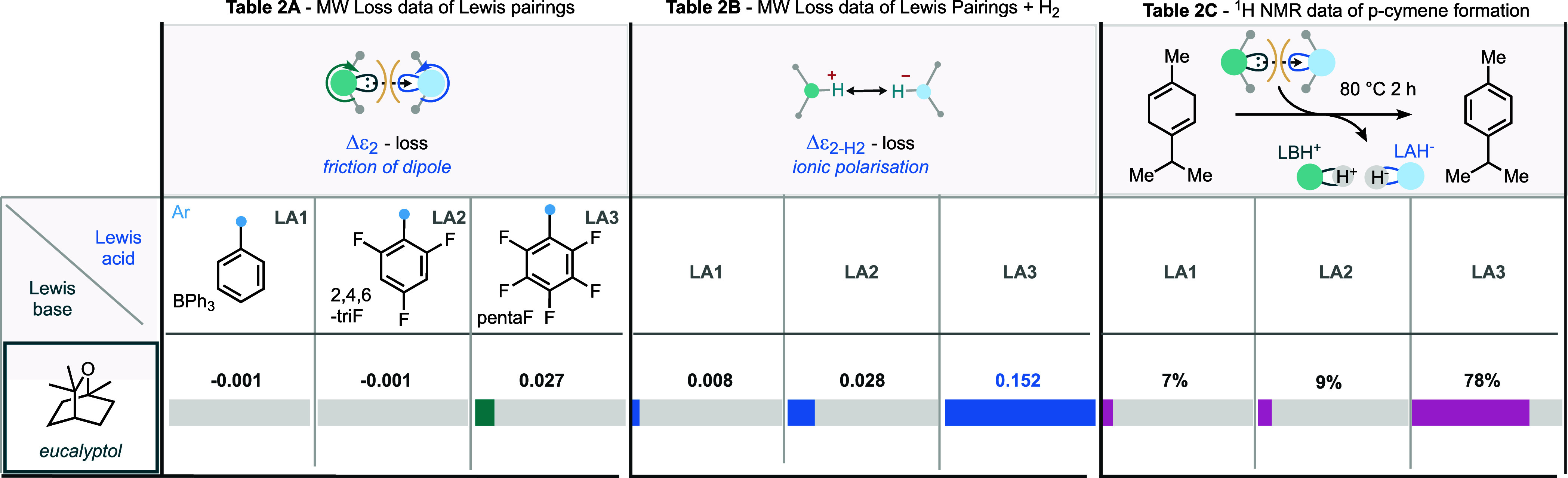
Screening for FLP Activity of Eucalyptol Lewis Base: (A) Loss Enhancement (Δε_2_) of Selected Eucalyptol Lewis Pairs, (B) Loss Enhancement (Δε_2–H_2__) for the H_2_ Response of Eucalyptol Lewis Pairs, and (C) Hydrogen Abstraction of Eucalyptol Lewis Pairs

## Conclusions

In conclusion, MDS has been demonstrated as a promising tool for the *in actu* detection of FLP encounter complexes. By measurement and calculation of the net microwave loss (Δε_2_), a FLP encounter complex can be detected. Both splitting of hydrogen gas and abstraction of hydrogen from γ-terpinene have shown a correlation with those samples with an enhanced microwave loss (Δε_2_), pointing toward reactivity through a FLP encounter complex, whereas those combinations with negligible or small Δε_2_ values do not demonstrate an ability to split or abstract hydrogen. This capability led us to postulate a novel FLP in the form of a LA3–eucalyptol pairing. Finally, the accuracy of MDS measurements and the numerical values provided in the microwave loss may point toward the degree of frustration; further work is planned to understand the correlation of these absolute values with catalytic competency and performance.

## Supporting Information Available

Experimental details; ^1^H, ^13^C, and ^19^F NMR spectra for all compounds; raw MDS data and additional experimental details; and materials and methods, including photographs of the experimental setup (PDF)
