# Supplementary material for: Use of Microwave Dielectric Spectroscopy for the In Actu Assessment of Frustrated Lewis Pair Encounter Complexes
Source: J Am Chem Soc. 2024 Jul 16;146(29):19809–17. doi: 10.1021/jacs.4c02736 (PMC11273348; doi:10.1021/jacs.4c02736)
Supplement: Supplementary file 1 — ja4c02736_si_001.pdf [file ja4c02736_si_001.pdf]

Supplementary Information

The use of Microwave Dielectric Spectroscopy for the *in actu* Assessment of Frustrated Lewis Pair Encounter Complexes

Cihang Yu, [Δ] Jamie A. Leitch,[Δ] Lukas Gierlichs,[°] Sampurna Das,[°] Adrian Porch,[*,¥] Rebecca L. Melen,[*,°] and Duncan L. Browne*[Δ]

Δ Department of Pharmaceutical and Biological Chemistry, University College London, School of Pharmacy, 29-39 Brunswick Square, Bloomsbury, London, W1CN 1AX, United Kingdom

° Cardiff Catalysis Institute, School of Chemistry, Cardiff University, Translational Research Hub, Maindy Road, Cathays, Cardiff, CF24 4HQ, Cymru/Wales, United Kingdom

¥ Centre for High Frequency Engineering, School of Engineering, Cardiff University, Queen's Buildings, Newport Road, Cardiff, Cardiff, CF24 3AA, United Kingdom

Table of contents

Contents

Table of contents 2
Experimental Procedures 3
General Information 3
S1. Synthesis of Lewis acids and base 4
S2. Microwave measurements 7
S2.1. Microwave spectroscopy design 7
S2.2. Sample tubes and plugs 8
S2.3. Resonant Microwave Applicators 9
S2.4. Calibration using known mixtures and extraction of complex permittivity 10
S3 MDS analysis of Lewis Pairings 13
S3.1 General information and flow chart guide 13
S3.2 Microwave measurement of Lewis pairs 15
S3.2.1 Key result of FLP microwave response (Table 1A) 15
S3.2.2 Raw data of FLP microwave response (Table 1A) 17
S3.3 Concentration studies (Figure 1a) 21
S3.4 Frequency study (Figure 1b) 21
S3.5 Lewis acidity comparison with $PMes_3$ (Figure 2) 23
S3.5.1 MDS measurement 23
S3.5.2 NMR measurement and water influence 23
S3.6 Microwave measurement of Lewis pairs hydrogen activation (Table 1B) 25
Key result of Lewis pairs hydrogen activation (Table 1B) 26
Raw data of Lewis pairs hydrogen activation (Table 1B) 27
NMR of hydrogenation reaction 28
S3.7 Control experiment - leaving the FLP sample open to air and the resultant MDS response 32
S5 NMR Spectra 34
S6 Reference 63

Experimental Procedures

General Information

Unless stated otherwise, all air-sensitive reactions and measurement were performed under an inert atmosphere, either within a nitrogen-filled glovebox, or using a Schlenk lines equipped with an inline gas purification column containing Drierite. Microwave dielectric spectroscopy measurement samples were prepared under inert atmosphere in glove box/Schlenk into an airtight FEP tube dried under vacuum overnight. All glassware used in air-sensitive operations were pre-dried in an oven (150 ˚C) overnight prior to use. Dry solvent and reagents are required to avoid the formation of water adducts with organoborane.

Dry toluene and other solvent were obtained from solvent purification system (SPS-Mbraun, SPS-800). All other solvents and commercial reagents were kept with activated molecular sieves and used without further purification unless stated otherwise.

Room temperature refers to 20−25 ˚C. All reactions involving heating were conducted using DrySyn blocks and a contact thermoprobe linked to the hotplate. *In vacuo* refers to reduced pressure of rotary evaporator. Organoboranes were prepared according to literature method[1-3]. $B(C_6F_5)_3$ used in γ-terpinene hydrogen extraction experiment was obtained from TCI as a commercial reagent and kept under inert atmosphere as a stock solution.

Commercially available chemicals were obtained from various chemical retailers (Sigma-Aldrich, Acros, Alfa Asear, TCI, Fluorochem) and were used as delivered. ^{1}H, ^{11}B, ^{19}F and ^{31}P NMR spectra were recorded on a Bruker Avance 400 or 500 MHz spectrometers. Chemical shifts are expressed as parts per million (ppm, δ) downfield of tetramethylsilane (TMS) and are referenced to $CDCl_3$ (7.26/77.16 ppm) and Toluene (2.11/21.37 ppm). The description of signals includes s = singlet, d = doublet, t = triplet, q = quartet, and m = multiplet, br. = broad. All coupling constants are absolute values and are expressed in Hertz (Hz).

S1. Synthesis of Lewis acids and base

The synthetic procedure of organoborane was synthesized based on published procedures.[1-6]

S1.1 *Synthesis of tris(pentafluorophenyl)borane (**LA3, PentaF**):*

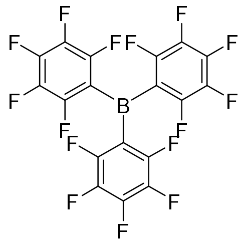

Synthesized according to a literature procedure.[1]In a dry 3-neck round bottom flask, magnesium (2.43 g, 100 mmol, 3.0 equiv.) was activated via the addition of 1 iodine bead. Dry Et_2O was added, before the slow addition of desired 1-bromo pentafluoro benzene derivative (24.70 g, 100 mmol, 3.0 equiv.). Cooling at 0 °C is necessary to ensure there is no thermal runaway. The reaction mixture was left to stir for 2 hours to ensure a complete reaction. The reaction mixture was then transferred to a solution of $BF_3{\cdot}OEt_2$ (4.73 g, 33 mmol, 1.0 equiv.) in toluene in a Schlenk flask. Et_2O solvent was removed via an external solvent trap. The solution was heated under reflux at 100 °C overnight (16 hours). Toluene was removed via an external solvent trap. The resulting brown cake was extracted with 3 × 60 mL of hexane, before being left in the freezer at -50 °C to crystallize. This was then subjected to a two-fold sublimation (120 °C, 1 x 10-3 mbar). Upon initial crystallization ~5 mL of Et_2O is added and the vessel is returned to the freezer to obtain a pure white microcrystalline solid of tris(pentafluorophenyl)borane (11.95 g, 23 mmol, 70%). The spectroscopic data agrees with literature values.[1]

^{11}B NMR (128 MHz, $CDCl_3$, 298K) δ 59.79 (br., s); **^{19}F NMR** (376 MHz, $CDCl_3$, 298K) δ: -127.65 (br., s, 6F), -142.48 (br., s, 3F), -159.83 (br., s, 6F).

S1.2 *Synthesis of tris(2,4,6-trifluorophenyl)borane (**LA2, 2,4,6-triF**):*

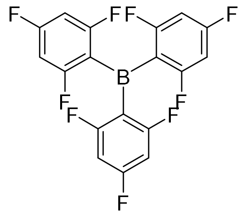

Synthesised according to a literature procedure.[1] In a dry 3-neck round bottom flask, magnesium (2.43 g, 100 mmol, 3.0 equiv.) was activated via the addition of 1 iodine bead. Dry Et_2O was added, before the slow addition of desired 1-Bromo-2,4,6-trifluorobenzene derivative (21.10 g, 100 mmol, 3.0 equiv.).

Cooling at 0 °C is necessary to ensure there is no thermal runaway. The reaction mixture was left to stir for 2 hours to ensure a complete reaction. The reaction mixture was then transferred to a solution of $BF_3.OEt_2$ (4.73 g, 33 mmol, 1.0 equiv.) in toluene in a Schlenk flask. Et_2O solvent was removed via an external solvent trap. The solution was heated under reflux at 100 °C overnight (16 hours). Toluene was removed via an external solvent trap. The resulting brown cake was extracted with 3 × 60 mL of hexane, before being left in the freezer at -50 °C to crystallize. This was then subjected to a two-fold sublimation (120 °C, 1 x 10-3 mbar). Upon

initial crystallization ~5 mL of Et_2O is added and the vessel is returned to the freezer to obtain a white solid of tris(2,4,6-trifluorophenyl)borane (11.45 g, 28 mmol, 85%). The spectroscopic data agrees with literature established values.[1]

^{1}H NMR (400 MHz, $CDCl_3$, 298 K) δ: 6.63 (dd, 6H); **^{11}B NMR** (128 MHz, $CDCl_3$, 298 K) δ: 59.77 (br., s); **^{19}F NMR** (376 MHz, $CDCl_3$, 298 K) δ: -95.75 (t, *J* = 9.3 Hz, 6F),
-100.30 (t, *J* = 9.7 Hz, 3F).

*S1.3 Synthesis of tris(3,4,5-trifluorophenyl) borane (**3,4,5-triF**):*

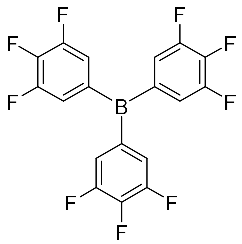

Synthesised according to a literature procedure.[1] In a dry 3-neck round bottom flask, magnesium (2.43 g, 100 mmol, 3.0 equiv.) was activated via the addition of 1 iodine bead. Dry Et_2O was added, before the slow addition of desired 1-Bromo-3,4,5 trifluorobenzene derivative (21.10 g, 100 mmol, 3.0 equiv.). Cooling at 0 °C necessary to ensure there is no thermal runaway. The reaction mixture was left to stir for 2 hours to ensure a complete reaction. The reaction mixture was then transferred to a solution of $BF_3.OEt_2$ (4.73 g, 33 mmol, 1.0 equiv.) in toluene in a Schlenk flask. Et_2O solvent was removed via an external solvent trap. The solution was heated under reflux at 100 °C overnight (16 hours). All volatiles were removed in vacuo, and sublimation of the resultant solid (120 °C, 1 x 10-3 mbar) resulted in oily yellow crystals. Upon initial crystallization ~5 mL of Et_2O is added and the vessel is returned to the freezer to obtain a white solid of tris (3,4,5-trifluorophenyl) borane (2.15 g, 5 mmol, 16%). The spectroscopic data agrees with literature values.[1]

^{1}H NMR (400 MHz, $CDCl_3$, 298 K) δ: 7.13-7.21 (m, 6H); **^{11}B NMR** (128 MHz, $CDCl_3$, 298 K) δ: 65.76 (br., s); **^{19}F NMR** (376 MHz, $CDCl_3$, 298 K) δ: -133.19 (d, *J* = 7.1 Hz, 6F),
-152.44 (t, *J* = 20.1 Hz, 3F).

*S1.4 Synthesis of tris(4-fluorophenyl)borane (**4-F**) :*

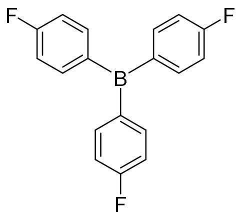

Synthesized according to a literature procedure.[2] In a dry 3-neck round bottom flask, magnesium (2.43 g, 100 mmol, 3.0 equiv.) was activated via the addition of 1 iodine bead. Dry Et_2O was added, before the slow addition of desired 1-Bromo 4-fluorobenzene derivative (17.50 g, 100 mmol, 3.0 equiv.). Cooling at 0 °C is necessary to ensure there is no thermal runaway. The reaction mixture was left to stir for 2 hours to ensure a complete reaction. The reaction mixture was then transferred to a solution of $BF_3.OEt_2$ (4.73 g, 33 mmol, 1.0 equiv.) in toluene in a Schlenk flask. Et_2O solvent was removed via an external solvent trap. The solution was heated under reflux at 100 °C overnight (16 hours). All volatiles were

removed in vacuo, and sublimation of the resultant solid (120 °C, 1 x 10-3 mbar) resulted in oily yellow crystals. Upon initial crystallization ~5 mL of Et_2O is added and the vessel is returned to the freezer to obtain a white solid of tris (4-fluorophenyl)borane (3.16 g, 11 mmol, 32%). The spectroscopic data agrees with literature established values. [2]

^{1}H NMR (400 MHz, $CDCl_3$, 298 K) δ: 7.63-7.57 (m, 2H), 7.19 – 7.14 (m, 2H); **^{11}B NMR** (128 MHz, $CDCl_3$, 298 K) δ: 66.17 (br., s); **^{19}F NMR** (376 MHz, $CDCl_3$, 298 K) δ: -107.81 (s, 3F).

*S1.5 Synthesis of tris(2,6-dimethylphenyl)phosphine **LB2***

To a suspension of magnesium turning (1.08 g, 45 mmol, 3 equiv.) in THF(1.0 M), 2-bromo-1,3-dimethylbenzene (6 ml, 45 mmol) were added dropwise. Thre reaction started

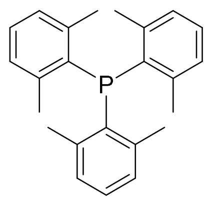

spontaneously after about 2 minutes, which was indicated by a colour change and warming of the reaction mixture. After the addition was completed, the mixture was heated under reflux for 3h. After cooling to ambient temperature, the Grignard reaction was then added to a solution of PCl_3 (1.31 ml, 15 mmol, 1.00 equiv.) in THF (0.4 M) at -78 °C. The reaction mixture was then warmed to ambient temperature and stirred for 18 h before it was quenched with aqueous saturated NH_4Cl. The organic layers were then extracted using Et_2O, washed with distilled water and brine, dried using $MgSO_4$, filtered and concentrated *in vacuo* to give the crude product, which was purified by recrystallisation in ethanol, gaves ***LB2*** as white crystal, 2.1 g, 40 % yield.

^{1}H NMR (400 MHz, $CDCl_3$, 298 K) δ: (400 MHz, $CDCl_3$) 2.09 (s, 18H, C*H₃*), 6.96 (dd, *J* 5.4,3.4 Hz, 6H, Ar-H), 7.11 (t, *J 7.5* Hz, 3H, Ar-H) **^{13}C NMR** δC (100 MHz, CDCl3) 23.0, 23.1, 128.2, 129.0, 134.8, 135.0,142.8,143.0;**^{31}P NMR** δP (162 MHz, CDCl3) −34.5 (s, 1P). The NMR data is in accordance with the literature. [6]

S2. Microwave measurements

S2.1. Microwave spectroscopy design

We measure the voltage transmission coefficient S_{21}[7] in the frequency domain using a vector network analyser (VNA, Agilent E5071C) under computer control, for two resonant microwave applicators (a cylindrical cavity resonator, CCR, or a parallel plate resonator, PPR). The simple, benchtop experimental set-up with the VNA and CCR is shown in Fig. S2.1(a), with typical measurement results shown in Fig. S2.1(b) for the transmitted power $|S_{21}|^2$. We read the marker data shown for each resonant mode directly from the VNA using a bespoke VBA program. To illustrate the measurement, we plot resonant traces in Fig. S2.1 for the CCR at 2.5 GHz without the sample (denoted by the subscript "0") and with the sample under test (denoted by the subscript "s"); f is the resonant frequency, f_B is the 3 dB (i.e. half-power) bandwidth and L is the insertion loss at resonance (in dB).

(a) (b)

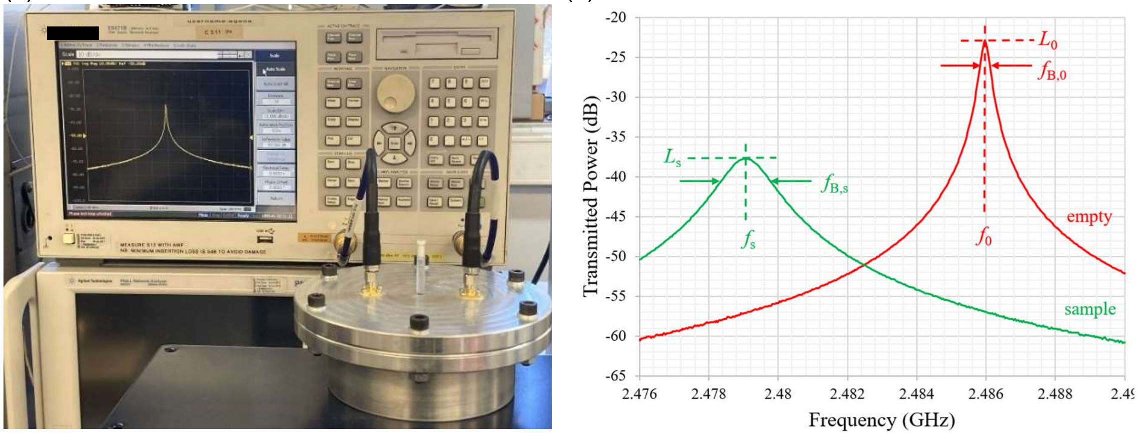

Figure 2.1: (a) Benchtop experimental set-up of the cylindrical cavity resonator connected to the vector network analyser via two identical, flexible coaxial cables, showing the on-screen measurement of transmitted power. (b) The resonant traces measured in the frequency domain and the associated marker data for an empty cavity (red), for a cavity filled with a lossy dielectric sample (green). The characteristic perturbations are a decreased resonant frequency, associated with sample polarisation, and an increased 3dB bandwidth (or reduced quality factor), associated with sample loss.

Referring to Fig. S2.1(b), we define the loaded quality factor as the dimensionless ratio $Q_L = f / f_B$, taken directly from the VNA marker data, where f_B is the 3dB (or half power) bandwidth. We correct all loaded (i.e. as-measured) quality factors Q_L for the effects of microwave coupling by first performing careful measurement of the microwave losses along the two flexible coaxial cables, allowing a true value of insertion loss L to be deduced. Then, unloaded Q can be calculated from $Q = Q_L/(1 - b)$, where $b = 10^{L/20}$; all Q factors used in the paper are unloaded values.

To mitigate the effects of systematic errors associated with sample volume, precise sample position in the resonator and small changes in ambient temperature from day-

to-day, we introduce normalised changes in resonant frequency and changes in loss using the dimensionless parameters experimental parameters *x* and *y*. These are defined by

$$x = \frac{f_{0,s} - f_{0,\mathrm{tube}}}{f_{0,\mathrm{tol}} - f_{0,\mathrm{tube}}} \qquad \text{(Equation S1)}$$

$$y = \frac{(1/2Q)_s - (1/2Q)_{\mathrm{tube}}}{(1/2Q)_{\mathrm{tol}} - (1/2Q)_{\mathrm{tube}}} \qquad \text{(Equation S2)}$$

where $f_{0,s}, f_{0,\mathrm{tol}}, f_{0,\mathrm{tube}}$ are the resonant frequencies with a sample, with pure toluene and with an empty sample tube, respectively; $Q_{0,s}, Q_{0,\mathrm{tol}}, Q_{0,\mathrm{tube}}$ are the corresponding values of unloaded *Q* factors. All of our computed changes in permittivity $\Delta\varepsilon_1$ and $\Delta\varepsilon_2$ are then relative to those of toluene, which has well-known microwave properties. Since we always use toluene as a solvent, this procedure also mitigates for any changes in the dielectric properties of toluene from batch to batch.

S2.2. Sample tubes and plugs

We use tubes formed from fluorinated ethylene propylene (FEP), of inner and outer diameters of 3.0 ± 0.1 mm and 5.0 ± 0.1 mm, respectively (Polyflon Technology Limited, Stafford, UK). FEP has excellent microwave properties, high chemical resistance, high optical transparency, low gas diffusion and can be thermoformed. FEP sample tubes are cut to a standard length of 90 mm and straightened (in pairs) using stainless steel rods of diameter 3.0 mm, length 200 mm held at 100 ˚C in an oven for 4 hours, followed by natural cooling back to room temperature.

(a) (b)

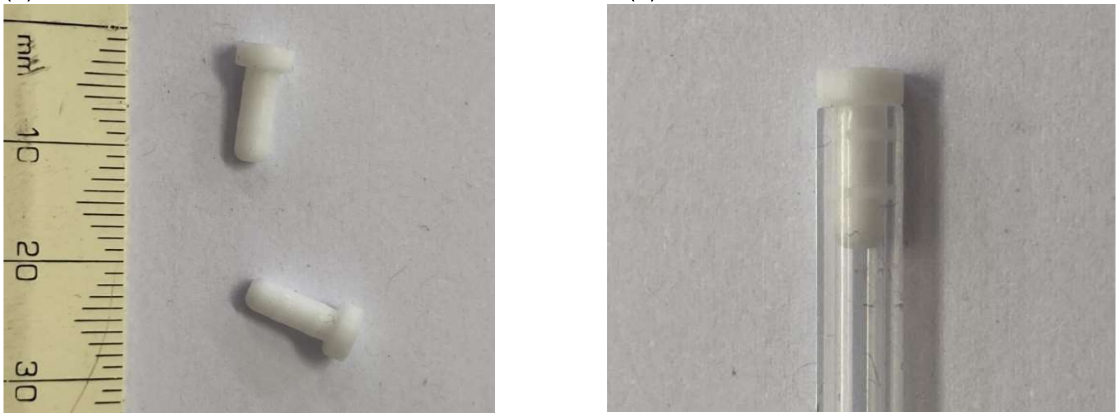

Figure S2.2: (a) The bespoke PFTE plugs used to seal the FEP sample tubes used in these experiments. (b) One end of a sealed FEP sample tube, showing the pair of raised collars on the plug body that provide a trap to prevent air-ingress.

Liquid samples are pipetted into the FEP tubes and sealed in a glove box under argon gas using a pair of CNC-manufactured PTFE plugs, leaving as little space as possible at the tube ends, typically 5 to 10 mm, so that the sample volume per tube is around 500 µl, with an active volume of 280 µl in the cylindrical cavity resonator and about 110 ml in the parallel plate resonator. The tight-fitting PTFE plugs are 9 mm long with a 7 mm long plug section, on which there are two raised, circular collar regions, as seen in Fig. S2.2.

S2.3. Resonant Microwave Applicators

The first type of microwave applicator used in these experiments is the cylindrical cavity resonator (CCR), shown in Fig. S3(a), of internal diameter 92 mm and internal length 40 mm, whose use for determining microwave dielectric properties has been described in detail elsewhere [7]. The CCR is CNC machined from aluminium and is operated in its TM_{010} (2.5GHz), TM_{011} (4.6GHz), TM_{020} (5.7GHz) and TM_{021} (6.9GHz) modes. In these TM modes the microwave electric field is directed parallel to the axis of the CCR, i.e. parallel to the FEP sample tube inserted along its axis and has a maximum electric field amplitude on-axis for each mode. The CCR is used for accurate, absolute measurements of the complex permittivity of toluene and for dilute toluene:solvent mixtures.

(a) (b)

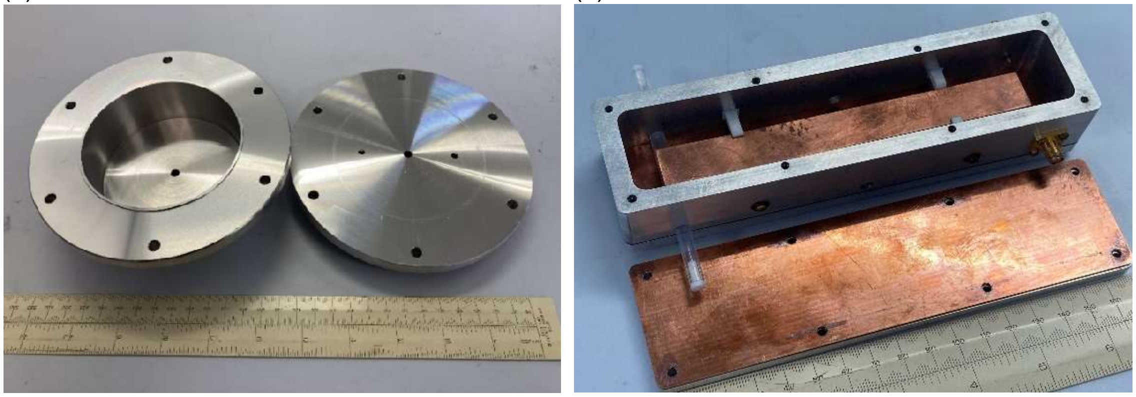

Figure S2.3: (a) The disassembled TM cylindrical resonant cavity (CCR), and (b) parallel plate resonator (PPR) used in all of the experiments. The sample tube is placed axially along the CCR and at one end of the PPR (as shown).

A half-wave, parallel plate resonator (PPR) has been developed to extend the measurements down to 1.0 GHz (Fig. S2.3(b)). This is a quasi-TEM structure which is resonant when its length is an integer number p of half-wavelengths, which defines the harmonic spectrum of the TEM_{00p} modes at frequencies $f_{00p} \approx pc/2\ell$. A fundamental of 1GHz corresponds to a length $\ell = 150\,\mathrm{mm}$, but in practice this needs to be reduced slightly to account for the additional effective length associated with the electric field spilling out of the ends of the resonator. Choosing $\ell = 140\mathrm{mm}$ sets the first four modes at 1.0. 2.0, 3.0 and 4.0 GHz when the PPR is empty. The sample enters the electric field antinode for each mode, which is at one end of the plate. The other end has a pair

of coaxial coupling probes (similar to those used to excite the CCR) allowing two-port VNA measurements of S_{21}. A scale diagram of the PPR and its critical dimensions is shown in Fig. S2.4.

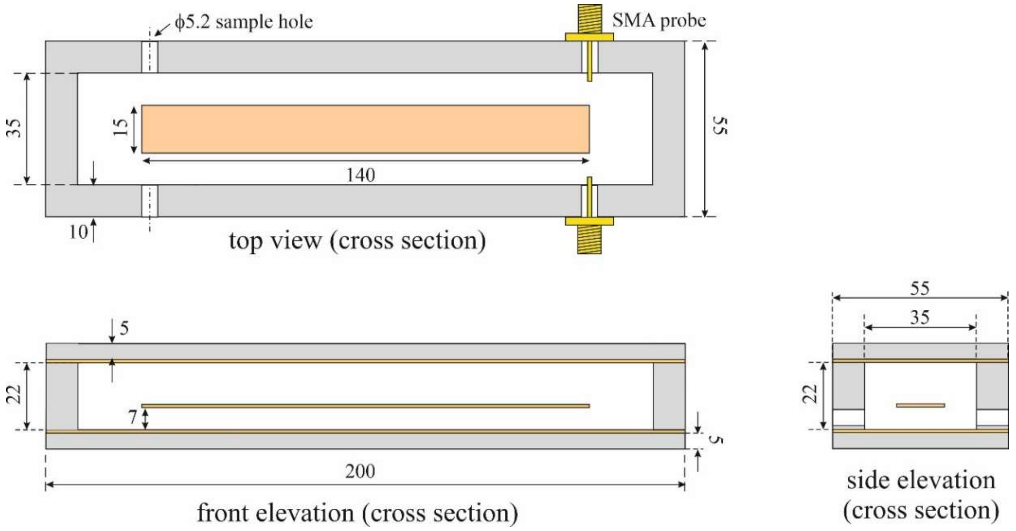

Figure S2.4: Scale drawing of the parallel plate resonator (PPR), with all dimensions in mm. The outer housing is made from aluminium and the central plate made from 1mm thick OFHC copper sheet, which also makes up the ground planes above and below the central plate. Symmetric, capacitive coupling is achieved using an identical pair of SMA probes.

S2.4. Calibration using known mixtures and extraction of complex permittivity

We limit our discussion to the measurement of samples in the low loss limit $\varepsilon_2 << \varepsilon_1 - 1$ (more correctly $\varepsilon_2^2 << (\varepsilon_1 - 1)^2$) of low permittivity $1 \leq \varepsilon_1 < 4$, which is a good approximation for all of the samples studied here. Then the normalised values of the polarisation and loss terms defined in Eqns. (S1) and (S2) can be written as theoretical functions of sample complex permittivity using

$$x(\varepsilon_1) \approx \frac{\varepsilon_1 - 1}{\varepsilon_{1,\text{tol}} - 1} \frac{e_1(\varepsilon_1)}{e_1(\varepsilon_{1,\text{tol}})} \qquad \text{(Equation S3)}$$

$$y(\varepsilon_1, \varepsilon_2) \approx \frac{\varepsilon_2 e_1^2(\varepsilon_1)}{\varepsilon_{2,\text{tol}} e_1^2(\varepsilon_{1,\text{tol}})} \qquad \text{(Equation S4)}$$

where we define $e_1(\varepsilon_1)$ to be the electric field depolarisation factor of the sample, i.e. the fraction E/E_0, where E is the internal field in the sample and E_0 is the applied field. For all of the CCR modes we have $e_1(\varepsilon_1) \approx 1$, since E_0 is applied parallel to the long length of the sample tubes and so there is little depolarisation. However, for all of the

PPR modes, $e_1(\varepsilon_1) < 1$ since E_0 is now predominantly perpendicular to the long dimension of the sample, becoming smaller as e_1 is increased and affecting the loss term *y* more than the polarisation term *x*.

Rather than determining $e_1(\varepsilon_1)$ theoretically or by simulation, which are difficult owing to the complex geometry of the PPR, we deduce it empirically by experiment using pure toluene and a range of dilute toluene:solvent mixtures (ethanol EtOH, dichloromethane DCM, diethyl ether Et_2O, hexane and acetonitrile MeCN, all at 2.5GHz and 20°C). These were first measured in the CCR, from which we calculate the known ("calibrated") values of $\varepsilon_1 = 1 + x_{\mathrm{CCR}}(\varepsilon_{1,\mathrm{tol}} - 1)$ and $\varepsilon_2 = \varepsilon_{2,\mathrm{tol}} y_{\mathrm{CCR}}$, used for the horizontal axis of Fig. S4 and for calculating the pure toluene data of Table S1 with the aid of Finite Element Modelling (LANL Superfish [9]). The empirical curve fit of Fig. S5 gives the following expression (to second order in ε_1^2), which is universal for all PPR modes

$$x(\varepsilon_1) = -0.06137\,\varepsilon_1^2 + 0.8288\varepsilon_1 - 0.6270 \qquad \text{(Equation S5)}$$

Hence, ε_1 is calculated for any PPR mode based on the experimental value of *x* for an unknown sample, by simple solution of the quadratic equation formed from Eqn. (S5).

Finally, to extract the loss term ε_2 from *y* for any unknown sample in any PPR mode, we combine Eqns. (S3) and (S4) to give

$$\varepsilon_2 \approx \varepsilon_{2,\mathrm{tol}} \left(\frac{\varepsilon_1 - 1}{\varepsilon_{1,\mathrm{tol}} - 1} \right)^2 \frac{y}{x^2} \qquad \text{(Equation S6)}$$

Note that the complex permittivity of toluene in the PPR extraction of the complex permittivity of any unknown sample is taken from Table S2.1.

Mode	Frequency (GHz)	Measurement (CCR) $\varepsilon = \varepsilon_1 - i\,\varepsilon_2$	Debye Model $\varepsilon = \varepsilon_1 - i\,\varepsilon_2$
TEM_{001} (PPR)	0.98	-	2.384 – *i* 0.00462
TEM_{002} (PPR)	1.97	-	2.383 – *i* 0.00923
TM_{010} (CCR)	2.48	2.38(2) – *i* 0.0116(1)	2.383 – *i* 0.01157
TEM_{003} (PPR)	2.97	-	2.382 – *i* 0.01378
TEM_{004} (PPR)	3.98	-	2.381 – *i* 0.01820
TM_{011} (CCR)	4.56	2.42(4) – *i* 0.0209(4)	2.380 – *i* 0.02064
TM_{020} (CCR)	5.65	2.36(2) – *i* 0.0250(3)	2.378 – *i* 0.02502
TM_{021} (CCR)	6.83	2.35(2) – *i* 0.0294(3)	2.375 – *i* 0.02939

Table S2.1: Complex permittivity of pure toluene at 20°C from calculated from CCR data with the aid of FEM simulation (Superfish), with the Debye model prediction [8] which can be used to extrapolate for the lower PPR frequencies. The FEP sample tube is measured to have e = 2.02(1) – j0.00, inner diameter 3.00(5) mm, outer diameter 5.00(5)mm. The CCR has a refined inner diameter 45.9(2) mm and inner length 39.2(2) mm, calculated from the frequencies of the four TM modes used here. The standard error associated with multiple samples is less than 0.5% in both parts of the complex permittivity, with the errors quoted due to systematic errors in all sizes.

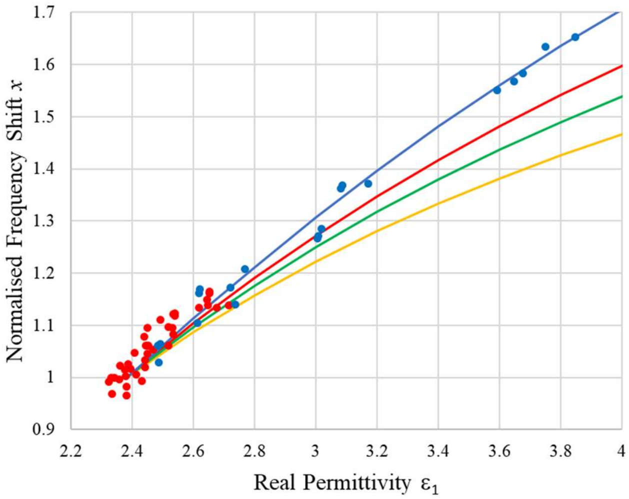

Figure S2.5: The normalised frequency shift for 63 dilute toluene solutions (red data points, with EtOH, DCM, Et_2O and hexane; blue data points with MeCN), evaluated by averaging the data from the 2GHz and 3GHz PPR modes, plotted as a function of real permittivity evaluated at 2.5GHz in the CCR. The blue solid curve is the empirical data fit used to calculate real permittivity for an unknown sample in the PPR. Other curves are attempts to model depolarisation using a “bare” cylindrical sample (orange), an isolated sample within the FEP tube (green) and an FEM model of the same tube separated by 1mm gaps from the copper PPR plates above and below (red). None of these models fit the data well, leading us to deduce the empirical, experimental formula of Eqn. (S5).

S3 MDS analysis of Lewis Pairings

S3.1 General information and flow chart guide

General information, following the measurement procedure as described in S2. All measurement were conducted in triplicate. As shown in Figure S3.1, samples of Lewis acid, samples of Lewis base, and samples of Lewis pair were prepared by dissolving solid components or adding liquid components in dry toluene in given concentration in glove box. The solution was transferred into a FEP tube and sealed with PTFE plug. A triplicate toluene and an empty FEP tube were prepared as well for background measurement. The samples were taken out and measured in microwave resonator. The data was collected in house made VBA program and processed in Excel spreadsheet to give ε_1, ε_2 and other parameters. The $\Delta\varepsilon_2$ data were calculated by Eqn.1.

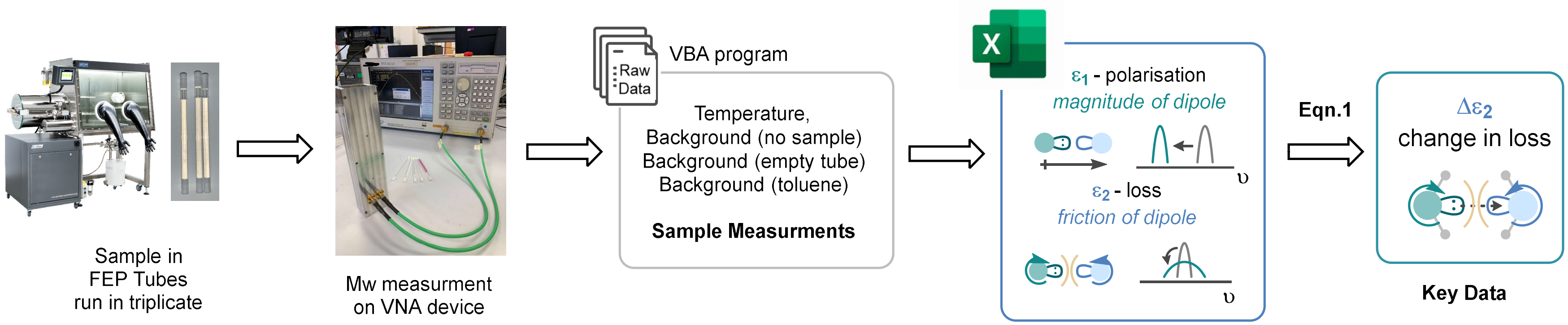

Figure S3.1 Flow chart of general procedure for MDS measurement on loss enhancement

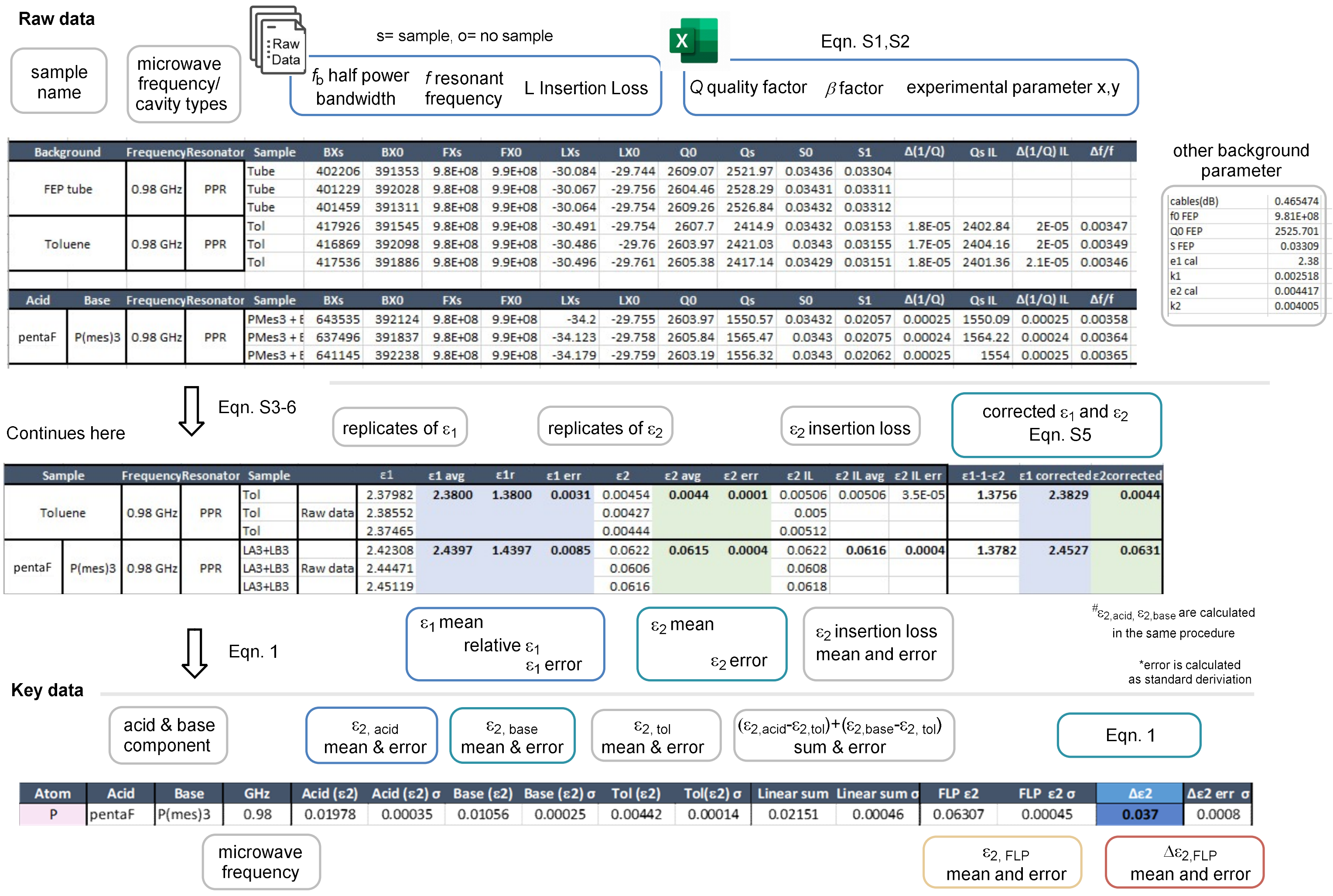

Background	Frequency	Resonator	Sample	BXs	BX0	FXs	FX0	LXs	LX0	Q0	Qs	S0	S1	Δ(1/Q)	Qs IL	Δ(1/Q) IL	Δf/f
FEP tube	0.98 GHz	PPR	Tube	402206	391353	9.8E+08	9.9E+08	-30.084	-29.744	2609.07	2521.97	0.03436	0.03304				
			Tube	401229	392028	9.8E+08	9.9E+08	-30.067	-29.756	2604.46	2528.29	0.03431	0.03311				
			Tube	401459	391311	9.8E+08	9.9E+08	-30.064	-29.754	2609.26	2526.84	0.03432	0.03312				
Toluene	0.98 GHz	PPR	Tol	417926	391545	9.8E+08	9.9E+08	-30.491	-29.754	2607.7	2414.9	0.03432	0.03153	1.8E-05	2402.84	2E-05	0.00347
			Tol	416869	392098	9.8E+08	9.9E+08	-30.486	-29.76	2603.97	2421.03	0.0343	0.03155	1.7E-05	2404.16	2E-05	0.00349
			Tol	417536	391886	9.8E+08	9.9E+08	-30.496	-29.761	2605.38	2417.14	0.03429	0.03151	1.8E-05	2401.36	2.1E-05	0.00346

Acid	Base	Frequency	Resonator	Sample	BXs	BX0	FXs	FX0	LXs	LX0	Q0	Qs	S0	S1	Δ(1/Q)	Qs IL	Δ(1/Q) IL	Δf/f
pentaF	P(mes)3	0.98 GHz	PPR	PMes3 + E	643535	392124	9.8E+08	9.9E+08	-34.2	-29.755	2603.97	1550.57	0.03432	0.02057	0.00025	1550.09	0.00025	0.00358
				PMes3 + E	637496	391837	9.8E+08	9.9E+08	-34.123	-29.758	2605.84	1565.47	0.0343	0.02075	0.00024	1564.22	0.00024	0.00364
				PMes3 + E	641145	392238	9.8E+08	9.9E+08	-34.179	-29.759	2603.19	1556.32	0.0343	0.02062	0.00025	1554	0.00025	0.00365

cables(dB)	0.465474
f0 FEP	9.81E+08
Q0 FEP	2525.701
S FEP	0.03309
e1 cal	2.38
k1	0.002518
e2 cal	0.004417
k2	0.004005

Sample		Frequency	Resonator	Sample		ε1	ε1 avg	ε1r	ε1 err	ε2	ε2 avg	ε2 err	ε2 IL	ε2 IL avg	ε2 IL err	ε1-1-ε2	ε1 corrected	ε2corrected
Toluene		0.98 GHz	PPR	Tol		2.37982	**2.3800**	**1.3800**	**0.0031**	0.00454	**0.0044**	**0.0001**	0.00506	0.00506	3.5E-05	**1.3756**	**2.3829**	**0.0044**
				Tol	Raw data	2.38552				0.00427			0.005					
				Tol		2.37465				0.00444			0.00512					
pentaF	P(mes)3	0.98 GHz	PPR	LA3+LB3		2.42308	**2.4397**	**1.4397**	**0.0085**	0.0622	**0.0615**	**0.0004**	0.0622	**0.0616**	**0.0004**	**1.3782**	**2.4527**	**0.0631**
				LA3+LB3	Raw data	2.44471				0.0606			0.0608					
				LA3+LB3		2.45119				0.0616			0.0618					

Atom	Acid	Base	GHz	Acid (ε2)	Acid (ε2) σ	Base (ε2)	Base (ε2) σ	Tol (ε2)	Tol(ε2) σ	Linear sum	Linear sum σ	FLP ε2	FLP ε2 σ	Δε2	Δε2 err σ
P	pentaF	P(mes)3	0.98	0.01978	0.00035	0.01056	0.00025	0.00442	0.00014	0.02151	0.00046	0.06307	0.00045	**0.037**	0.0008

Figure S3.2 Annotated spreadsheet example of FLP PenaF-PMes₃ (LA3-LB3) measurement and data processing

S3.2 Microwave measurement of Lewis pairs

Under nitrogen atmosphere in a glovebox, Lewis acid (0.15 mmol, 1 eq.), Lewis base (0.15 mmol, 1 eq.), were dissolved in dry toluene (1.5 ml). The resulting 0.1 M Lewis pair solution was transferred into a FEP tube (OD 5.00+/-0.10mm, >7 cm long) and sealed with PTFE stopper. The tubes were taken out from glovebox and measured in microwave dielectric spectrum resonator. Empty tube, dry toluene, Lewis acid (0.1 M) and Lewis base (0.1 M) were measured separately in triplicates.

The data obtained were workout following the flowchart of Figure S3.1 and Figure S3.2. The raw data were processed in an excel spreadsheet. An example of pentaF-PMes$_3$ (LA3-LB3) Lewis pair measurement were shown in Figure S3.2. The ε_1 and ε_2 value were calculated by Eqn. S1-4,6 and corrected by Eqn. S5. Then the $\Delta\varepsilon_2$ value is calculated by Eqn. 1 (main text).

S3.2.1 Key result of FLP microwave response (Table 1A)

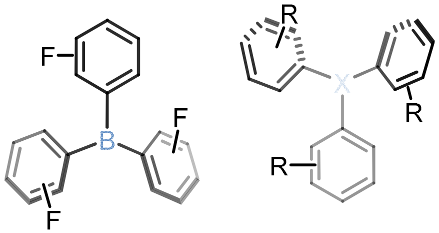

The result of Lewis acid, Lewis base and Lewis pair measurements listed in table 1A were collected as below.

Atom	Acid	Base	GHz	Acid (ε2)	Acid (ε2) σ	Base (ε2)	Base (ε2) σ	Tol (ε2)	Tol(ε2) σ	Linear sum	Linear sum σ	FLP ε2	FLP ε2 σ	Δε2	Δε2 err σ
P	BPh3	PPh3	0.98	0.00491	0.00013	0.01147	0.00011	0.00442	0.00014	0.00755	0.00022	0.00699	0.00005	**-0.005**	-0.0001
P	BPh3	P(o-tol)3	0.98	0.00491	0.00013	0.00606	0.00012	0.00442	0.00014	0.00213	0.00023	0.00564	0.00008	**-0.001**	-0.0001
P	BPh3	P(xyl)3	0.98	0.00491	0.00013	0.00832	0.00014	0.00442	0.00014	0.00440	0.00024	0.00934	0.00020	**0.001**	0.0000
P	BPh3	P(mes)3	0.98	0.00491	0.00013	0.01056	0.00025	0.00442	0.00014	0.00664	0.00032	0.01366	0.00009	**0.003**	0.0001
P	245triF	PPh3	0.98	0.01062	0.00018	0.01147	0.00011	0.00442	0.00014	0.01326	0.00025	0.01361	0.00019	**-0.004**	-0.0001
P	245triF	P(o-tol)3	0.98	0.01062	0.00018	0.00606	0.00012	0.00442	0.00014	0.00785	0.00026	0.01078	0.00020	**-0.001**	-0.0001
P	245triF	P(xyl)3	0.98	0.01062	0.00018	0.00832	0.00014	0.00442	0.00014	0.01011	0.00026	0.01847	0.00017	**0.004**	0.0001
P	245triF	P(mes)3	0.98	0.01062	0.00018	0.01056	0.00025	0.00442	0.00014	0.01235	0.00034	0.02643	0.00046	**0.010**	0.0003
P	pentaF	PPh3	0.98	0.01978	0.00035	0.01147	0.00011	0.00442	0.00014	0.02242	0.00039	0.04440	0.00034	**0.018**	0.0003
P	pentaF	P(o-tol)3	0.98	0.01978	0.00035	0.00606	0.00012	0.00442	0.00014	0.01700	0.00040	0.02990	0.00055	**0.008**	0.0003
P	pentaF	P(xyl)3	0.98	0.01978	0.00035	0.00832	0.00014	0.00442	0.00014	0.01927	0.00040	0.10259	0.00154	**0.079**	0.0020
P	pentaF	P(mes)3	0.98	0.01978	0.00035	0.01056	0.00025	0.00442	0.00014	0.02151	0.00046	0.06307	0.00045	**0.037**	0.0008
O	BPh3	Et2O	0.98	0.00491	0.00013	0.00386	0.00003	0.00442	0.00014	-0.00006	0.00019	0.00418	0.00003	**0.000**	-0.0006
O	BPh3	CPME	0.98	0.00491	0.00013	0.00459	0.00008	0.00442	0.00014	0.00067	0.00021	0.00475	0.00007	**0.000**	-0.0001
O	BPh3	TBME	0.98	0.00491	0.00013	0.00520	0.00011	0.00442	0.00014	0.00128	0.00022	0.00576	0.00014	**0.000**	0.0000
O	BPh3	Eucalypto	0.98	0.00491	0.00013	0.00613	0.00008	0.00442	0.00014	0.00221	0.00021	0.00598	0.00008	**-0.001**	-0.0001
O	245triF	Et2O	0.98	0.01062	0.00018	0.00386	0.00003	0.00442	0.00014	0.00565	0.00023	0.00989	0.00012	**0.000**	0.0000
O	245triF	CPME	0.98	0.01062	0.00018	0.00459	0.00008	0.00442	0.00014	0.00638	0.00024	0.01005	0.00033	**-0.001**	0.0000
O	245triF	TBME	0.98	0.01062	0.00018	0.00520	0.00011	0.00442	0.00014	0.00699	0.00025	0.00994	0.00010	**-0.001**	-0.0001
O	245triF	Eucalypto	0.98	0.01062	0.00018	0.00613	0.00008	0.00442	0.00014	0.00792	0.00024	0.01114	0.00046	**-0.001**	-0.0001
O	pentaF	Et2O	0.98	0.01978	0.00035	0.00386	0.00003	0.00442	0.00014	0.01481	0.00038	0.15096	0.00415	**0.132**	0.0050
O	pentaF	CPME	0.98	0.01978	0.00035	0.00459	0.00008	0.00442	0.00014	0.01554	0.00039	0.07180	0.00095	**0.052**	0.0015
O	pentaF	TBME	0.98	0.01978	0.00035	0.00520	0.00011	0.00442	0.00014	0.01615	0.00039	0.23285	0.00194	**0.212**	0.0042
O	pentaF	Eucalypto	0.98	0.01978	0.00035	0.00613	0.00008	0.00442	0.00014	0.01708	0.00039	0.04884	0.00012	**0.027**	0.0006
														*Precipitation	

S3.2.2 Raw data of FLP microwave response (Table 1A)

Background

The sample tube and the solvent of dry toluene were calibrated for each measurement and check against the standard value.

Sample	Frequency	Resonator	Sample	BXs	BX0	FXs	FX0	LXs	LX0	Q0	Qs	S0	S1
FEP Tube	0.98 GHz	PPR	TUBE1	404868.6	394171.4	9.81E+08	9.86E+08	-29.0554	-28.6865	2602.016	2515.809	0.038194	0.036606
			TUBE2	403942.7	394318.7	9.81E+08	9.86E+08	-29.0612	-28.686	2601.061	2521.446	0.038196	0.036581
			TUBE3	402779.7	393539.4	9.82E+08	9.86E+08	-29.0336	-28.6834	2606.251	2529.951	0.038207	0.036698

Background	Frequency	Resonator	Sample	BXs	BX0	FXs	FX0	LXs	LX0	Q0	Qs	S0	S1	Δ(1/Q)	Qs IL	Δ(1/Q) IL	Δf/f	ε1 avg	ε1 avg	ε1r	ε1 err	ε2	ε2 avg	ε2 err	ε2 IL	ε2 IL avg	ε2 IL err	ε1-1-ε2	ε1 corrected	ε2corrected
Toluene	0.98 GHz	PPR	Tol	421602.2	396713.7	9.78E+08	9.86E+08	-30.4976	-29.7931	2573.761	2394.001	0.034168	0.031506	1.69E-05	2381.829	1.9E-05	0.003541	2.389783	**2.3800**	**1.3800**	**0.0060**	0.0043	**0.0044**	**0.0001**	0.0048	**0.0048**	**0.0000**	**1.3756**	**2.3829**	**0.0044**
			Tol	421726.3	396465.1	9.78E+08	9.86E+08	-30.4984	-29.7931	2575.376	2393.343	0.034168	0.031503	1.7E-05	2381.589	1.91E-05	0.003519	2.381101				0.0043			0.0048					
			Tol	423321.9	370470.6	9.78E+08	9.86E+08	-30.4936	-29.5096	2759.317	2384.438	0.035302	0.031521	1.86E-05	2382.973	1.88E-05	0.003488	2.369115				0.0047			0.0048					

Lewis Acids

Acid	Frequency	Resonator	Sample	BXs	BX0	FXs	FX0	LXs	LX0	Q0	Qs	S0	S1	Δ(1/Q)	Qs IL	Δ(1/Q) IL	Δf/f	ε1	ε1 avg	ε1r	ε1 err	ε2	ε2 avg	ε2 err	ε2 IL	ε2 IL avg	ε2 IL err	ε1-1-ε2	ε1 corrected	ε2corrected
BPh3	0.98 GHz	PPR	BPh3	423132.4	396615.7	9.78E+08	9.86E+08	-30.5171	-29.7942	2574.384	2385.189	0.034164	0.031436	1.85E-05	2376.315	2E-05	0.003532	2.386517	**2.3992**	**1.3992**	**0.0108**	0.0047	**0.0049**	**0.0001**	0.0051	**0.0051**	**0.0000**	**1.3944**	**2.4050**	**0.0049**
			BPh3	423803.1	396562.9	9.77E+08	9.86E+08	-30.5266	-29.7947	2574.721	2381.122	0.034162	0.031401	1.92E-05	2373.638	2.05E-05	0.00362	2.420783				0.0048			0.0052					
			BPh3	424977.2	396798.8	9.78E+08	9.86E+08	-30.5134	-29.7942	2573.196	2374.844	0.034164	0.031449	2.03E-05	2377.356	1.98E-05	0.003542	2.390432				0.0051			0.0050					
246triF	0.98 GHz	PPR	BTF 1	441592.7	393081.5	9.77E+08	9.86E+08	-30.8817	-29.7397	2597.713	2281.918	0.034375	0.03014	4.04E-05	2267.492	4.32E-05	0.003566	2.407863	**2.4047**	**1.4047**	**0.0167**	0.0102	**0.0105**	**0.0002**	0.0109	**0.0112**	**0.0002**	**1.3942**	**2.4113**	**0.0106**
			BTF 2	443988	391429.8	9.77E+08	9.86E+08	-30.919	-29.7206	2608.875	2269.5	0.034451	0.030011	4.28E-05	2257.492	4.51E-05	0.00348	2.374189				0.0108			0.0114					
			BTF 3	443232.8	393755.6	9.77E+08	9.86E+08	-30.9145	-29.7456	2593.2	2273.07	0.034352	0.030027	4.21E-05	2258.704	4.49E-05	0.003626	2.431944				0.0106			0.0113					
pentaF	0.98 GHz	PPR	BCF	483683.4	394913.1	9.77E+08	9.86E+08	-31.6771	-29.7851	2585.579	2077.919	0.0342	0.027506	8.05E-05	2070.835	8.21E-05	0.003649	2.432174	**2.4022**	**1.4022**	**0.0152**	0.0203	**0.0196**	**0.0004**	0.0207	**0.0200**	**0.0003**	**1.3826**	**2.4085**	**0.0198**
			BCF	480083.8	394242.4	9.78E+08	9.86E+08	-31.6028	-29.7895	2589.933	2094.225	0.034182	0.027742	7.67E-05	2089.14	7.79E-05	0.003546	2.391735				0.0194			0.0196					
			BCF	479263.8	396786.1	9.78E+08	9.86E+08	-31.6054	-29.7907	2573.313	2097.838	0.034178	0.027734	7.59E-05	2088.492	7.8E-05	0.003523	2.382764				0.0191			0.0197					
345triF	0.98 GHz	PPR	3,4,5-BTF	431125.4	390965	9.77E+08	9.86E+08	-29.7605	-28.6412	2623.051	2346.384	0.038393	0.033751	3.32E-05	2325.565	3.70E-05	0.003554	2.391	**2.3880**	**1.3880**	**0.0060**	0.0078	**0.0070**	**0.0010**	0.0087	**0.0083**	**0.0005**	**1.3811**	**2.3920**	**0.0070**
			3,4,5-BTF	431787.1	391172.9	9.77E+08	9.86E+08	-29.7638	-28.6405	2621.666	2342.728	0.038396	0.033738	3.38E-05	2324.658	3.72E-05	0.003567	2.396				0.0080			0.0088					
			3,4,5-BTF	419237.9	391619.7	9.77E+08	9.86E+08	-29.6409	-28.6432	2618.646	2414.178	0.038384	0.034219	2.12E-05	2358.95	3.09E-05	0.003516	2.376				0.0050			0.0073					
26diF	0.98 GHz	PPR	2,6-BDF	427983.6	390421.6	9.78E+08	9.86E+08	-29.715	-28.6424	2627.028	2364.307	0.038388	0.033928	3.09E-05	2343.979	3.45E-05	0.003697	2.458	**2.4050**	**1.4050**	**0.0300**	0.0069	**0.0066**	**0.0002**	0.0077	**0.0075**	**0.0002**	**1.3984**	**2.4117**	**0.0067**
			2,6-BDF	425119.4	390382.5	9.78E+08	9.86E+08	-29.664	-28.6418	2627.3	2381.363	0.038391	0.034128	2.78E-05	2358.265	3.20E-05	0.003432	2.354				0.0062			0.0072					
			2,6-BDF	427146.9	390535.4	9.78E+08	9.86E+08	-29.6919	-28.6419	2626.271	2369.492	0.03839	0.034019	2.99E-05	2350.458	3.34E-05	0.003557	2.403				0.0067			0.0075					
4F	0.98 GHz	PPR	4-F	422561.3	400160.8	9.79E+08	9.86E+08	-28.6611	-28.0524	2571.232	2410.717	0.041748	0.038922	1.62E-05	2402.775	1.76E-05	0.002574	2.331	**2.3710**	**1.3710**	**0.0240**	0.0051	**0.0054**	**0.0002**	0.0055	**0.0056**	**0.0001**	**1.3656**	**2.3727**	**0.0054**
			4-F	423984.6	399691	9.79E+08	9.86E+08	-28.6659	-28.0477	2574.314	2402.4	0.04177	0.038901	1.76E-05	2401.405	1.78E-05	0.002645	2.368				0.0055			0.0056					
			4-F	424112.3	399410.4	9.79E+08	9.86E+08	-28.6812	-28.0477	2576.123	2401.293	0.04177	0.038832	1.78E-05	2397.01	1.86E-05	0.002733	2.414				0.0056			0.0058					

Lewis Bases

Base	Frequency	Resonator		BXs	BX0	FXs	FX0	LXs	LX0	Q0	Qs	S0	S1	Δ(1/Q)	Qs IL	Δ(1/Q) IL	Δf/f	ε1	ε1 avg	ε1r	ε1 err	ε2	ε2 avg	ε2 err	ε2 IL	ε2 IL avg	ε2 IL err	ε1-1-ε2	ε1 corrected	ε2corrected
				448993.1	396865.3	9.77E+08	9.86E+08	-31.017	-29.7934	2572.766	2243.642	0.034167	0.029677	4.49E-05	2239.353	4.58E-05	0.003575	2.403156	**2.4115**	**1.4115**	**0.0046**	0.0113	**0.0113**	**0.0001**	0.0115	**0.0116**	**0.0001**	**1.4002**	**2.4193**	**0.0115**
PPh3	0.98 GHz	PPR		448149.4	396824.9	9.77E+08	9.86E+08	-31.0236	-29.7976	2572.983	2247.722	0.03415	0.029655	4.41E-05	2237.602	4.61E-05	0.003615	2.419062				0.0111			0.0116					
				449620.1	396921.3	9.77E+08	9.86E+08	-31.0327	-29.7931	2572.406	2240.336	0.034168	0.029624	4.56E-05	2235.18	4.66E-05	0.003598	2.412393				0.0115			0.0118					
				425596	394566	9.77E+08	9.86E+08	-30.6779	-29.8908	2586.533	2369.643	0.033783	0.030856	2.4E-05	2361.124	2.55E-05	0.003563	2.392901	**2.3890**	**1.3890**	**0.0091**	0.0058	**0.0060**	**0.0001**	0.0062	0.0064	0.0001	**1.3830**	**2.3932**	**0.0061**
P(o-tol)3	0.98 GHz	PPR		427276	394972	9.77E+08	9.86E+08	-30.7113	-29.8907	2583.881	2359.979	0.033783	0.030737	2.57E-05	2351.775	2.72E-05	0.003588	2.402466				0.0062			0.0066					
				426587	394749	9.77E+08	9.86E+08	-30.7021	-29.8878	2585.371	2364.057	0.033794	0.03077	2.5E-05	2354.346	2.67E-05	0.003509	2.371778				0.0061			0.0065					
				431277.9	393355.6	9.78E+08	9.86E+08	-30.4712	-29.4903	2599.028	2340.701	0.035377	0.031599	2.99E-05	2325.39	3.27E-05	0.00356	2.365576	**2.3905**	**1.3905**	**0.0140**	0.0077	**0.0079**	**0.0001**	0.0084	**0.0085**	**0.0001**	**1.3826**	**2.3949**	**0.0079**
P(xyl)3	0.98 GHz	PPR		432948.9	393803	9.77E+08	9.86E+08	-30.4888	-29.4911	2596.067	2331.217	0.035374	0.031535	3.16E-05	2320.515	3.36E-05	0.003686	2.41405				0.0081			0.0086					
				432342.4	393446	9.78E+08	9.86E+08	-30.4796	-29.491	2598.423	2334.704	0.035374	0.031569	3.1E-05	2323.067	3.31E-05	0.003628	2.391801				0.0079			0.0085					
				440050	391738.5	9.77E+08	9.86E+08	-30.9638	-29.7578	2606.399	2289.381	0.034307	0.02986	4.09E-05	2271.557	4.43E-05	0.003541	2.40642	**2.4043**	**1.4043**	**0.0038**	0.0102	**0.0105**	**0.0003**	0.0111	**0.0110**	**0.0000**	**1.3938**	**2.4109**	**0.0106**
P(mes)3	0.98 GHz	PPR		443109.7	392005.5	9.77E+08	9.86E+08	-30.9587	-29.7605	2604.603	2273.595	0.034296	0.029877	4.39E-05	2272.912	4.4E-05	0.003549	2.409484				0.0110			0.0110					
				440061.2	391983.6	9.77E+08	9.86E+08	-30.9611	-29.7583	2604.782	2289.399	0.034305	0.029869	4.09E-05	2272.276	4.42E-05	0.003517	2.396943				0.0102			0.0110					
				419210.7	392512.8	9.77E+08	9.86E+08	-30.4146	-29.7218	2602.099	2408.295	0.03445	0.031809	1.94E-05	2405.028	2E-05	0.003643	2.408274	**2.3877**	**1.3877**	**0.0108**	0.0038	**0.0039**	**0.0000**	0.0039	**0.0040**	**0.0000**	**1.3838**	**2.3917**	**0.0039**
Et2O	0.98 GHz	PPR		419747.6	392690	9.78E+08	9.86E+08	-30.4229	-29.7189	2600.953	2405.298	0.034461	0.031778	1.99E-05	2402.644	2.04E-05	0.003577	2.382716				0.0039			0.0040					
				419293	392474	9.78E+08	9.86E+08	-30.417	-29.7282	2602.288	2408.027	0.034424	0.0318	1.95E-05	2404.344	2.01E-05	0.003549	2.371991				0.0038			0.0039					
				420842	393883	9.77E+08	9.86E+08	-30.5998	-29.8865	2591.068	2397.047	0.033799	0.031135	1.91E-05	2383.136	2.16E-05	0.003586	2.401669	**2.3779**	**1.3779**	**0.0122**	0.0046	**0.0046**	**0.0001**	0.0052	**0.0051**	**0.0001**	**1.3733**	**2.3805**	**0.0046**
CPME	0.98 GHz	PPR		420017	394378	9.77E+08	9.86E+08	-30.5822	-29.8909	2587.765	2402.16	0.033782	0.031198	1.83E-05	2388.125	2.07E-05	0.003483	2.361416				0.0044			0.0050					
				421158	394094	9.77E+08	9.86E+08	-30.5937	-29.8886	2589.659	2395.493	0.033791	0.031156	1.94E-05	2384.864	2.13E-05	0.003506	2.370582				0.0047			0.0052					
				419881.7	392052	9.77E+08	9.86E+08	-30.5283	-29.7678	2604.378	2403.318	0.034268	0.031395	2.02E-05	2392.143	2.21E-05	0.003472	2.378894	**2.3850**	**1.3850**	**0.0043**	0.0050	**0.0052**	**0.0001**	0.0055	**0.0055**	**0.0000**	**1.3798**	**2.3886**	**0.0052**
TBME	0.98 GHz	PPR		420348.3	393033.7	9.77E+08	9.86E+08	-30.5219	-29.7719	2597.837	2400.684	0.034251	0.031418	2.06E-05	2393.962	2.18E-05	0.003481	2.382719				0.0051			0.0054					
				421327.4	392439.9	9.77E+08	9.86E+08	-30.533	-29.7667	2601.833	2394.941	0.034272	0.031378	2.16E-05	2390.78	2.23E-05	0.003508	2.393291				0.0054			0.0056					
				426891	394237.3	9.77E+08	9.86E+08	-30.5817	-29.7641	2589.816	2362.786	0.034279	0.031199	2.48E-05	2361.156	2.51E-05	0.003678	2.440537	**2.4240**	**1.4240**	**0.0083**	0.0062	**0.0060**	**0.0001**	0.0062	**0.0061**	**0.0001**	**1.4180**	**2.4341**	**0.0061**
Eucalyptol	0.98 GHz	PPR		425777.1	394518.7	9.77E+08	9.86E+08	-30.5617	-29.7619	2587.994	2369.304	0.034288	0.031271	2.37E-05	2366.778	2.41E-05	0.00361	2.414077				0.0059			0.0060					
				426416.3	394903	9.77E+08	9.86E+08	-30.5679	-29.7626	2585.468	2365.677	0.034285	0.031249	2.43E-05	2365.032	2.44E-05	0.003619	2.417484				0.0060			0.0061					

Lewis Pairs

FLP																															
Acid	Base	Frequency	Resonator	Sample	BXs	BX0	FXs	FX0	LXs	LX0	Q0	Qs	S0	S1	Δ(1/Q)	Qs IL	Δ(1/Q) IL	Δf/f	ε1	ε1 avg	ε1r	ε1 err	ε2	ε2 avg	ε2 err	ε2 IL	ε2 IL avg	ε2 IL err	ε1-1-ε2	ε1 correcte	ε2corrected
BPH3	PPh3	0.98 GHz	PPR	BPh3 + PP	434523.5	393079	9.78E+08	9.86E+08	-30.7208	-29.714	2598.423	2320.951	0.034481	0.030707	3.51E-05	2319.092	3.54E-05	0.003571	2.380704	**2.3952**	**1.3952**	**0.0074**	0.0069	**0.0070**	**0.0001**	0.0069	**0.0070**	**0.0000**	**1.3883**	**2.4003**	**0.0070**
				BPh3 + PP	434653.5	393074.4	9.77E+08	9.86E+08	-30.7256	-29.7137	2598.457	2320.102	0.034482	0.03069	3.52E-05	2317.75	3.57E-05	0.00362	2.399523				0.0069			0.0070					
				BPh3 + PP	435376.9	393128.5	9.77E+08	9.86E+08	-30.7325	-29.7162	2598.072	2316.153	0.034472	0.030665	3.59E-05	2315.846	3.6E-05	0.003635	2.405396				0.0071			0.0071					
BPH3	P(o-tol)3	0.98 GHz	PPR	10_BPh3_	425007	394197	9.77E+08	9.86E+08	-30.6607	-29.8891	2588.977	2373.108	0.033789	0.030917	2.34E-05	2365.954	2.46E-05	0.00355	2.38772	**2.3953**	**1.3953**	**0.0062**	0.0057	**0.0056**	**0.0001**	0.0060	**0.0060**	**0.0000**	**1.3897**	**2.4004**	**0.0056**
				11_BPh3_	425155	394378	9.77E+08	9.86E+08	-30.6727	-29.8877	2587.804	2372.056	0.033795	0.030874	2.35E-05	2362.583	2.52E-05	0.003601	2.407647				0.0057			0.0061					
				12_BPh3_	424055	394869	9.77E+08	9.86E+08	-30.6669	-29.8917	2584.544	2378.364	0.033779	0.030895	2.24E-05	2364.212	2.49E-05	0.003557	2.39051				0.0054			0.0061					
BPH3	P(xyl)3	0.98 GHz	PPR	F1 2	438487.4	394246.8	9.77E+08	9.86E+08	-30.827	-29.7481	2589.95	2298.462	0.034342	0.030331	3.81E-05	2297.194	3.84E-05	0.003602	2.402501	**2.4151**	**1.4151**	**0.0115**	0.0090	**0.0092**	**0.0002**	0.0091	**0.0093**	**0.0001**	**1.4059**	**2.4235**	**0.0093**
				F1 4	440828.3	393457.4	9.77E+08	9.86E+08	-30.857	-29.7484	2595.141	2285.801	0.034341	0.030226	4.05E-05	2289.032	3.99E-05	0.003693	2.437982				0.0096			0.0095					
				F1 6	438197.9	393647.6	9.77E+08	9.86E+08	-30.8338	-29.7499	2593.869	2299.91	0.034335	0.030307	3.78E-05	2295.331	3.87E-05	0.003608	2.404822				0.0090			0.0092					
BPH3	P(mes)3	0.98 GHz	PPR	PMes3 + B	453194.8	391421.5	9.77E+08	9.86E+08	-31.1556	-29.7589	2608.524	2221.376	0.034303	0.029207	5.42E-05	2220.435	5.44E-05	0.003589	2.425697	**2.4291**	**1.4291**	**0.0052**	0.0135	**0.0134**	**0.0001**	0.0136	**0.0136**	**0.0001**	**1.4157**	**2.4401**	**0.0137**
				PMes3 + B	452282	392046.7	9.77E+08	9.86E+08	-31.1654	-29.7608	2604.352	2225.707	0.034295	0.029174	5.34E-05	2217.855	5.5E-05	0.003624	2.439298				0.0133			0.0137					
				PMes3 + B	452059.2	391699.5	9.77E+08	9.86E+08	-31.1489	-29.7565	2606.727	2227.028	0.034312	0.02923	5.31E-05	2222.217	5.41E-05	0.003581	2.422288				0.0133			0.0135					
246triF	PPh3	0.98 GHz	PPR	PPh3 1	452941.8	393901.5	9.77E+08	9.86E+08	-31.0921	-29.7375	2592.32	2223.11	0.034384	0.029419	5.2E-05	2211.59	5.43E-05	0.003556	2.404112	**2.4302**	**1.4302**	**0.0130**	0.0131	**0.0133**	**0.0002**	0.0137	**0.0140**	**0.0002**	**1.4169**	**2.4414**	**0.0136**
				PPh3 2	453238.8	394126.2	9.77E+08	9.86E+08	-31.1174	-29.7436	2590.775	2221.235	0.03436	0.029333	5.23E-05	2204.972	5.57E-05	0.003656	2.443429				0.0132			0.0140					
				PPh3 3	455245.5	394515.5	9.77E+08	9.86E+08	-31.1407	-29.7407	2588.247	2211.267	0.034371	0.029255	5.44E-05	2198.87	5.69E-05	0.003655	2.443042				0.0137			0.0143					
246triF	P(o-tol)3	0.98 GHz	PPR	Potol3 1	442219.8	395002.8	9.77E+08	9.86E+08	-30.8831	-29.7445	2585.013	2278.623	0.034356	0.030135	4.1E-05	2267.122	4.32E-05	0.003587	2.416177	**2.4210**	**1.4210**	**0.0061**	0.0103	**0.0106**	**0.0002**	0.0109	**0.0111**	**0.0002**	**1.4104**	**2.4305**	**0.0108**
				Potol3 2	444769.8	393644.5	9.77E+08	9.86E+08	-30.9295	-29.7403	2593.975	2265.087	0.034373	0.029975	4.36E-05	2254.68	4.57E-05	0.00363	2.433166				0.0110			0.0115					
				Potol3 3	442745.9	394007.3	9.77E+08	9.86E+08	-30.8942	-29.7418	2591.57	2275.84	0.034367	0.030097	4.15E-05	2264.154	4.38E-05	0.00358	2.41372				0.0105			0.0110					
246triF	P(xyl)3	0.98 GHz	PPR	Poxyl3 1	471505.3	394519.6	9.77E+08	9.86E+08	-31.4211	-29.7416	2588.207	2133.059	0.034368	0.028326	7.1E-05	2126.996	7.23E-05	0.003613	2.426565	**2.4168**	**1.4168**	**0.0122**	0.0179	**0.0182**	**0.0002**	0.0182	**0.0186**	**0.0002**	**1.3986**	**2.4255**	**0.0185**
				Poxyl3 2	473734	394012.6	9.77E+08	9.86E+08	-31.4751	-29.7363	2591.596	2122.615	0.034389	0.02815	7.33E-05	2113.419	7.53E-05	0.003625	2.431296				0.0185			0.0190					
				Poxyl3 3	472894.5	394615.1	9.77E+08	9.86E+08	-31.4535	-29.7465	2587.532	2126.746	0.034348	0.02822	7.23E-05	2118.839	7.41E-05	0.003527	2.392548				0.0182			0.0187					
246triF	P(mes)3	0.98 GHz	PPR	Pmes3 1	501197.9	394722.5	9.77E+08	9.86E+08	-31.965	-29.7408	2586.888	2003.122	0.034371	0.026606	0.000101	1994.355	0.000104	0.003625	2.431209	**2.4317**	**1.4317**	**0.0003**	0.0255	**0.0259**	**0.0005**	0.0261	**0.0264**	**0.0004**	**1.4059**	**2.4432**	**0.0264**
				Pmes3 2	505948.7	393913.1	9.77E+08	9.86E+08	-32.0387	-29.741	2592.201	1983.852	0.03437	0.026381	0.000106	1977.047	0.000108	0.003626	2.431648				0.0268			0.0272					
				Pmes3 3	500184.2	394359.2	9.77E+08	9.86E+08	-31.9592	-29.7408	2589.27	2007.213	0.034371	0.026624	0.0001	1995.72	0.000103	0.003627	2.432258				0.0253			0.0260					
pentaF	PPh3	0.98 GHz	PPR	pentaF + F	572692.2	394485.8	9.77E+08	9.86E+08	-33.1668	-29.7804	2588.379	1747.125	0.034218	0.023171	0.000174	1738.365	0.000176	0.003651	2.412593	**2.3930**	**1.3930**	**0.0098**	0.0438	**0.0442**	**0.0003**	0.0445	**0.0449**	**0.0003**	**1.3488**	**2.3977**	**0.0444**
				pentaF + F	576888.7	394915.5	9.77E+08	9.86E+08	-33.2308	-29.7848	2585.519	1734.243	0.034201	0.023	0.000178	1725.293	0.000181	0.003576	2.383776				0.0449			0.0456					
				pentaF + F	573099.4	395506.6	9.77E+08	9.86E+08	-33.1766	-29.7917	2581.579	1745.974	0.034174	0.023144	0.000174	1736.364	0.000177	0.003573	2.382494				0.0439			0.0447					
pentaF	P(o-tol)3	0.98 GHz	PPR	13_pentaF	524102	394141	9.77E+08	9.86E+08	-32.5059	-29.8903	2589.33	1912.507	0.033785	0.025	0.000125	1901.527	0.000128	0.003667	2.433552	**2.4248**	**1.4248**	**0.0134**	0.0303	**0.0293**	**0.0006**	0.0310	**0.0300**	**0.0006**	**1.3954**	**2.4349**	**0.0299**
				14_pentaF	516426	394479	9.77E+08	9.86E+08	-32.3682	-29.8928	2587.088	1941.904	0.033775	0.025399	0.000117	1932.705	0.000119	0.003577	2.39848				0.0284			0.0290					
				15_pentaF	520401	395345	9.77E+08	9.86E+08	-32.4348	-29.8933	2581.413	1926.471	0.033773	0.025205	0.000121	1917.561	0.000123	0.00369	2.44232				0.0294			0.0299					
pentaF	P(xyl)3	0.98 GHz	PPR	PXyl3 pent	819196	393824.2	9.77E+08	9.86E+08	-36.2164	-29.7488	2592.716	1212.66	0.034339	0.016308	0.000428	1217.569	0.000424	0.003676	2.431266	**2.4389**	**1.4389**	**0.0038**	0.1013	**0.1000**	**0.0015**	0.1005	**0.0995**	**0.0016**	**1.3389**	**2.4518**	**0.1026**
				PXyl3 pent	821071.2	394484.5	9.77E+08	9.86E+08	-36.2633	-29.7481	2588.378	1209.747	0.034342	0.016221	0.00043	1210.898	0.000429	0.003705	2.442738				0.1018			0.1016					
				PXyl3 pent	801091.3	393681	9.77E+08	9.86E+08	-36.0323	-29.7396	2593.749	1240.47	0.034376	0.016658	0.000409	1244.085	0.000407	0.003705	2.442692				0.0969			0.0964					
pentaF	P(mes)3	0.98 GHz	PPR	PMes3 + p	643534.6	392123.6	9.77E+08	9.86E+08	-34.2003	-29.7546	2603.97	1550.571	0.03432	0.020571	0.000249	1550.091	0.000249	0.003583	2.423082	**2.4397**	**1.4397**	**0.0085**	0.0622	**0.0615**	**0.0004**	0.0622	**0.0616**	**0.0004**	**1.3782**	**2.4527**	**0.0631**
				PMes3 + p	637496.1	391837.1	9.77E+08	9.86E+08	-34.1231	-29.7584	2605.84	1565.466	0.034305	0.020755	0.000243	1564.219	0.000243	0.003637	2.444709				0.0606			0.0608					
				PMes3 + p	641145.1	392238	9.77E+08	9.86E+08	-34.1789	-29.7585	2603.187	1556.32	0.034304	0.020622	0.000247	1554.004	0.000248	0.003654	2.451189				0.0616			0.0618					

FLP																															
**Acid**	**Base**	**Frequency**	**Resonator**	**Sample**	**BXs**	**BX0**	**FXs**	**FX0**	**LXs**	**LX0**	**Q0**	**Qs**	**S0**	**S1**	**Δ(1/Q)**	**Qs IL**	**Δ(1/Q) IL**	**Δf/f**	**ε1**	**ε1 avg**	**ε1r**	**ε1 err**	**ε2**	**ε2 avg**	**ε2 err**	**ε2 IL**	**ε2 IL avg**	**ε2 IL err**	**ε1-1-ε2**	**ε1 correcte**	**ε2corrected**
				BPh3 + Die	421277.3	393170.8	9.78E+08	9.86E+08	-30.4259	-29.7141	2597.818	2396.62	0.03448	0.031767	2.15E-05	2401.801	2.06E-05	0.003542	2.369392	**2.3908**	**1.3908**	**0.0108**	0.0042	**0.0042**	**0.0000**	0.0040	**0.0041**	**0.0000**	**1.3866**	**2.3953**	**0.0042**
BPH3	Et2O	0.98 GHz	PPR	BPh3 + Die	420727.6	392884.9	9.77E+08	9.86E+08	-30.4432	-29.7182	2599.664	2399.409	0.034464	0.031704	2.1E-05	2396.858	2.14E-05	0.003619	2.399166				0.0041			0.0042					
				BPh3 + Die	420975.1	392898.5	9.77E+08	9.86E+08	-30.4326	-29.7175	2599.582	2398.065	0.034467	0.031743	2.12E-05	2399.888	2.09E-05	0.003631	2.403862				0.0042			0.0041					
				16_BPh3_	421510	394387	9.77E+08	9.86E+08	-30.6034	-29.8901	2587.72	2393.277	0.033785	0.031122	1.98E-05	2382.116	2.18E-05	0.00356	2.391705	**2.3797**	**1.3797**	**0.0083**	0.0048	**0.0048**	**0.0001**	0.0053	**0.0053**	**0.0000**	**1.3750**	**2.3826**	**0.0047**
BPH3	CPME	0.98 GHz	PPR	17_BPh3_	420759	394247	9.77E+08	9.86E+08	-30.5988	-29.8905	2588.632	2397.761	0.033784	0.031138	1.9E-05	2383.419	2.15E-05	0.003489	2.363807				0.0046			0.0052					
				18_BPh3_	421668	394809	9.77E+08	9.86E+08	-30.6061	-29.8941	2584.91	2392.405	0.03377	0.031112	2E-05	2381.352	2.19E-05	0.00354	2.383734				0.0048			0.0053					
				TBME + BF	421511	393007	9.77E+08	9.86E+08	-30.5525	-29.7733	2598.018	2393.685	0.034246	0.031308	2.18E-05	2385.268	2.33E-05	0.003525	2.39997	**2.3989**	**1.3989**	**0.0161**	0.0055	**0.0057**	**0.0001**	0.0058	**0.0060**	**0.0001**	**1.3932**	**2.4046**	**0.0058**
BPH3	TBME	0.98 GHz	PPR	TBME + BF	423456.7	392507.4	9.77E+08	9.86E+08	-30.5759	-29.7677	2601.39	2382.656	0.034268	0.031223	2.38E-05	2378.631	2.45E-05	0.00345	2.370429				0.0059			0.0061					
				TBME + BF	422667.3	392294.8	9.77E+08	9.86E+08	-30.5758	-29.7622	2602.863	2386.77	0.03429	0.031224	2.3E-05	2378.663	2.45E-05	0.003591	2.426281				0.0058			0.0061					
				F1 1	424989.7	393403.1	9.77E+08	9.86E+08	-30.5655	-29.748	2595.507	2373.713	0.034342	0.031258	2.43E-05	2369.668	2.5E-05	0.003609	2.405319	**2.4118**	**1.4118**	**0.0080**	0.0058	**0.0059**	**0.0001**	0.0059	**0.0068**	**0.0009**	**1.4059**	**2.4196**	**0.0060**
BPH3	Eucalyptol	0.98 GHz	PPR	F1 3	426180.6	394004.4	9.77E+08	9.86E+08	-30.5681	-29.745	2591.574	2367.077	0.034354	0.031248	2.55E-05	2368.943	2.52E-05	0.003601	2.402242				0.0060			0.0060					
				F1 5	425483.1	393942.2	9.77E+08	9.86E+08	-30.5791	-29.7524	2591.904	2370.704	0.034325	0.031209	2.49E-05	2365.839	2.57E-05	0.003667	2.427764				0.0059			0.0061					
				Et2O 1	439790.2	394112.3	9.77E+08	9.86E+08	-30.8237	-29.7384	2590.918	2291.784	0.03438	0.030342	3.85E-05	2283.168	4.01E-05	0.00355	2.401634	**2.3995**	**1.3995**	**0.0205**	0.0097	**0.0098**	**0.0001**	0.0101	**0.0103**	**0.0001**	**1.3897**	**2.4053**	**0.0099**
246triF	Et2O	0.98 GHz	PPR	Et2O 2	441171.6	394733.5	9.77E+08	9.86E+08	-30.8538	-29.7476	2586.741	2284.174	0.034344	0.030237	3.99E-05	2275.02	4.17E-05	0.003632	2.433943				0.0101			0.0105					
				Et2O 3	439769.2	394656.2	9.77E+08	9.86E+08	-30.8277	-29.7428	2587.299	2292.086	0.034363	0.030328	3.84E-05	2282.086	4.03E-05	0.003452	2.362993				0.0097			0.0102					
				CPME 1	438268.1	394192.3	9.77E+08	9.86E+08	-30.8102	-29.738	2590.394	2299.758	0.034382	0.030389	3.7E-05	2286.826	3.94E-05	0.003592	2.418429	**2.4274**	**1.4274**	**0.0046**	0.0093	**0.0099**	**0.0003**	0.0099	**0.0103**	**0.0002**	**1.4176**	**2.4381**	**0.0101**
246triF	CPME	0.98 GHz	PPR	CPME 2	442693.4	394051.3	9.77E+08	9.86E+08	-30.8725	-29.7413	2591.285	2276.192	0.034369	0.030172	4.15E-05	2269.982	4.27E-05	0.003621	2.429867				0.0104			0.0107					
				CPME 3	440168.6	394650.1	9.77E+08	9.86E+08	-30.8358	-29.7453	2587.31	2289.527	0.034353	0.0303	3.89E-05	2279.894	4.08E-05	0.003632	2.433942				0.0098			0.0103					
				TBME 1	439280.5	394744.4	9.77E+08	9.86E+08	-30.8073	-29.7448	2586.697	2294.425	0.034355	0.0304	3.8E-05	2287.629	3.93E-05	0.003617	2.428211	**2.4309**	**1.4309**	**0.0083**	0.0096	**0.0097**	**0.0001**	0.0099	**0.0102**	**0.0002**	**1.4211**	**2.4422**	**0.0099**
246triF	TBME	0.98 GHz	PPR	TBME 2	440552.2	394666.7	9.77E+08	9.86E+08	-30.8588	-29.7444	2587.208	2287.271	0.034357	0.03022	3.93E-05	2273.663	4.2E-05	0.003663	2.446396				0.0099			0.0106					
				TBME 3	439816.2	394464.8	9.77E+08	9.86E+08	-30.827	-29.751	2588.462	2291.527	0.034331	0.030331	3.85E-05	2282.286	4.03E-05	0.003591	2.417998				0.0097			0.0102					
				Euc 1	440756.1	394157.4	9.77E+08	9.86E+08	-30.8744	-29.7415	2590.581	2286.236	0.034368	0.030166	3.95E-05	2269.481	4.28E-05	0.003598	2.42057	**2.4379**	**1.4379**	**0.0090**	0.0100	**0.0109**	**0.0005**	0.0108	**0.0115**	**0.0003**	**1.4271**	**2.4507**	**0.0111**
246triF	Eucalyptol	0.98 GHz	PPR	Euc 2	446274.1	395510.1	9.77E+08	9.86E+08	-30.9605	-29.7586	2581.532	2257.147	0.034301	0.029868	4.52E-05	2246.39	4.73E-05	0.003654	2.442826				0.0114			0.0119					
				Euc 3	445838.2	394194.2	9.77E+08	9.86E+08	-30.9426	-29.7461	2590.282	2259.454	0.03435	0.02993	4.47E-05	2251.183	4.64E-05	0.003673	2.450443				0.0113			0.0117					
				pentaF + D	987581.3	395403.9	9.77E+08	9.86E+08	-37.8812	-29.7824	2582.351	1003.018	0.03421	0.013465	0.000598	1000.291	0.000601	0.003811	2.474732	**2.4670**	**1.4670**	**0.0072**	0.1509	**0.1453**	**0.0042**	0.1516	**0.1461**	**0.0041**	**1.3217**	**2.4860**	**0.1510**
pentaF	Et2O	0.98 GHz	PPR	pentaF + D	976013	394639.5	9.77E+08	9.86E+08	-37.7803	-29.7737	2587.448	1015.071	0.034245	0.013623	0.000586	1012.142	0.000589	0.003809	2.47374				0.1479			0.1486					
				pentaF + D	934606.6	395107.6	9.77E+08	9.86E+08	-37.4124	-29.7889	2584.223	1060.734	0.034184	0.014212	0.000544	1056.563	0.000548	0.003754	2.452567				0.1372			0.1382					
				19_pentaF	691198	393701	9.77E+08	9.86E+08	-34.9027	-29.8881	2592.25	1441.13	0.033793	0.018971	0.000296	1434.12	0.000299	0.00375	2.465834	**2.4434**	**1.4434**	**0.0116**	0.0718	**0.0699**	**0.0010**	0.0726	**0.0706**	**0.0010**	**1.3735**	**2.4572**	**0.0718**
pentaF	CPME	0.98 GHz	PPR	20_pentaF	679785	394625	9.77E+08	9.86E+08	-34.7483	-29.8936	2586.12	1465.941	0.033772	0.019312	0.000284	1460.347	0.000287	0.003677	2.437537				0.0689			0.0696					
				21_pentaF	679696	394713	9.77E+08	9.86E+08	-34.7605	-29.8914	2585.566	1466.133	0.03378	0.019284	0.000284	1458.257	0.000288	0.00365	2.426777				0.0689			0.0698					
				TBME + pe	1258055	392940	9.77E+08	9.86E+08	-40.0493	-29.7715	2598.522	784.8346	0.034253	0.010491	0.000878	782.4627	0.000882	0.003902	2.549684	**2.5319**	**1.5319**	**0.0116**	0.2193	**0.2176**	**0.0019**	0.2203	**0.2184**	**0.0021**	**1.3143**	**2.5683**	**0.2329**
pentaF	TBME	0.98 GHz	PPR	TBME + pe	1236456	392573.9	9.77E+08	9.86E+08	-39.8833	-29.767	2600.998	798.7872	0.034271	0.010693	0.000856	797.718	0.000858	0.003802	2.510075				0.2137			0.2142					
				TBME + pe	1260067	393235.6	9.77E+08	9.86E+08	-40.0604	-29.7694	2596.6	783.5976	0.034261	0.010477	0.00088	781.4481	0.000884	0.003867	2.535989				0.2198			0.2207					
				euc penta	589593.2	395442.8	9.77E+08	9.86E+08	-33.3839	-29.7659	2581.904	1695.699	0.034272	0.022596	0.000191	1695.02	0.000192	0.003679	2.441014	**2.4428**	**1.4428**	**0.0036**	0.0474	**0.0475**	**0.0001**	0.0475	**0.0473**	**0.0004**	**1.3953**	**2.4565**	**0.0488**
pentaF	Eucalyptol	0.98 GHz	PPR	euc penta	590915.9	394752.7	9.77E+08	9.86E+08	-33.4006	-29.764	2586.437	1691.843	0.034279	0.022553	0.000193	1691.701	0.000193	0.003671	2.437685				0.0478			0.0478					
				euc penta	589412.3	395196.3	9.77E+08	9.86E+08	-33.3297	-29.7624	2583.55	1696.427	0.034285	0.022738	0.000191	1705.881	0.000188	0.003701	2.449717				0.0474			0.0466					

S3.3 Concentration studies (Figure 1a)

The effect of sample concentration has been demonstrated by measuring a commercially available Lewis acid BPh_3 and Lewis base collidine in CCR (2.48 GHz). The ε_2 response were compared in figure 2 without background/toluene correction. The results are shown in table below and Figure 1a(main text).

Composition	ε 1	ε 1 err	ε 2	ε 2 err
toluene	2.370	0.005	0.01192	0.00002
0.01 M Coll + BPh_3	2.373	0.008	0.01820	0.00013
0.03 M Coll + BPh_3	2.381	0.004	0.03109	0.00046
0.1 M Coll + BPh_3	2.458	0.005	0.07834	0.00135
0.3 M Coll + BPh_3	2.542	0.003	0.12733	0.00175

S3.4 Frequency study (Figure 1b)

A set of Lewis base with pentaF (LA3) in toluene solution (0.1 M) were measured in a range of microwave frequency and resonator cavity, the preparation and measurement of samples following the same procedure of standard measurement. Data of ε_2 measurement taken in CAV(2.48 GHz) are presented as it is as discussed in S2.

Acid	Base	Frequency	Frequency	Resonator	Sample	BXs	BX0	FXs	FX0	LXs	LX0	Q0	Qs	S0	S1	Δ(1/Q)	Qs IL	Δ(1/Q) IL	Δf/f	ε1	ε1 avg	ε1r	ε1 err	ε2	ε2 avg	ε2 err	ε2 IL	ε2 IL avg	ε2 IL err	ε1-1-ε2	1 correcte	2corrected
pentaF	PPh3	0.98 GHz	1	PPR	pentaF + F	572692.2	394485.8	9.77E+08	9.86E+08	-33.1668	-29.7804	2588.379	1747.125	0.034218	0.023171	0.000174	1738.365	0.000176	0.003651	2.412593	**2.3930**	**1.392954**	**0.009826**	0.043786	**0.044**	**0.000343**	0.044513	**0.044935**	**0.000342**	**1.3488**	**2.3977**	**0.0444**
					pentaF + F	576888.7	394915.5	9.77E+08	9.86E+08	-33.2308	-29.7848	2585.519	1734.243	0.034201	0.023	0.000178	1725.293	0.000181	0.003576	2.383776				0.044858			0.045613					
					pentaF + F	573099.4	395506.6	9.77E+08	9.86E+08	-33.1766	-29.7917	2581.579	1745.974	0.034174	0.023144	0.000174	1736.364	0.000177	0.003573	2.382494				0.043881			0.04468					
		1.97 GHz	1.97	PPR	pentaF + F	862652.9	601082.6	1.99E+09	2E+09	-25.0335	-21.5538	3657.634	2449.82	0.090256	0.060463	0.000125	2413.383	0.000131	0.002958	2.411589	**2.4008**	**1.400764**	**0.005499**	0.034729	**0.035**	**0.00025**	0.036444	**0.036781**	**0.000259**	**1.3657**	**2.4068**	**0.0354**
					pentaF + F	868660.6	601118.8	1.99E+09	2E+09	-25.0932	-21.5532	3657.438	2431.898	0.090261	0.060049	0.000128	2395.814	0.000134	0.002921	2.393665				0.035566			0.03729					
					pentaF + F	864310.6	601212.3	1.99E+09	2E+09	-25.0452	-21.5521	3656.911	2444.985	0.090273	0.060381	0.000126	2409.93	0.000132	0.002928	2.397038				0.034954			0.03661					
		2.48 GHz	2.48	CAV	pentaF + F	670395.8	315619.9	2.48E+09	2.5E+09	-26.3883	-19.7112	0.052209	0.113055	3908.726	8927.026	0.000136	0.002719	3850.386	0.00014	2.411866	**2.4170**	**1.417033**	**0.002777**	0.035324	**0.035**	**2.13E-05**	0.03633	**0.03637**	**2.13E-05**	**1.3818**	**2.4258**	**0.0358**
					pentaF + F	669742.3	315680.3	2.48E+09	2.5E+09	-26.3816	-19.7118	0.052249	0.112919	3912.634	8923.933	0.000136	0.002738	3853.517	0.00014	2.421381				0.035257			0.036275					
					pentaF + F	669678.2	315679.5	2.48E+09	2.5E+09	-26.4096	-19.7029	0.052081	0.112875	3912.341	8923.504	0.000136	0.002731	3840.42	0.000141	2.417851				0.035262			0.036505					
		2.97 GHz	2.97	PPR	pentaF + F	1094417	762636.8	2.97E+09	2.99E+09	-22.1175	-19.0184	4476.968	2971.857	0.12293	0.086041	0.000105	3000.441	0.000101	0.003012	2.394633	**2.3820**	**1.382029**	**0.006453**	0.031929	**0.032**	**0.000222**	0.03095	**0.031333**	**0.000248**	**1.3498**	**2.3852**	**0.0323**
					pentaF + F	1101747	762703.7	2.97E+09	2.99E+09	-22.1834	-19.0211	4476.384	2950.092	0.122892	0.085391	0.000107	2975.652	0.000104	0.002977	2.378129				0.032687			0.031798					
					pentaF + F	1096973	762866.4	2.97E+09	2.99E+09	-22.1411	-19.0242	4475.201	2964.312	0.122848	0.085807	0.000105	2991.545	0.000102	0.002966	2.373326				0.03219			0.031253					
		3.98 GHz	3.98	PPR	pentaF + F	1339246	927707.5	3.98E+09	4E+09	-19.9785	-16.2853	5203.953	3342.138	0.170869	0.111686	9.92E-05	3263.358	0.000106	0.002791	2.383988	**2.3777**	**1.377658**	**0.004088**	0.031829	**0.032**	**0.000238**	0.034146	**0.034621**	**0.000281**	**1.3454**	**2.3802**	**0.0322**
					pentaF + F	1349265	927697.2	3.98E+09	4E+09	-20.0546	-16.2849	5204.056	3313.721	0.170876	0.110712	0.000102	3231.346	0.000109	0.002781	2.378973				0.032652			0.03512					
					pentaF + F	1343512	927842	3.98E+09	4E+09	-20.0138	-16.2912	5202.462	3329.925	0.170752	0.111233	0.0001	3248.475	0.000108	0.002763	2.370011				0.032181			0.034597					
pentaF	P(o-tol)3	0.98 GHz	1	PPR	13_pentaF	524102	394141	9.77E+08	9.86E+08	-32.5059	-29.8903	2589.33	1912.507	0.033785	0.025	0.000125	1901.527	0.000128	0.003667	2.433552	**2.4248**	**1.424784**	**0.013393**	0.030283	**0.029**	**0.000554**	0.031016	**0.029974**	**0.000594**	**1.3954**	**2.4349**	**0.0299**
					14_pentaF	516426	394479	9.77E+08	9.86E+08	-32.3682	-29.8928	2587.088	1941.904	0.033775	0.025399	0.000117	1932.705	0.000119	0.003577	2.39848				0.028363			0.028958					
					15_pentaF	520401	395345	9.77E+08	9.86E+08	-32.4348	-29.8933	2581.413	1926.471	0.033773	0.025205	0.000121	1917.561	0.000123	0.00369	2.44232				0.029364			0.029949					
		1.97 GHz	1.97	PPR	13_pentaF	804328	595982	1.97E+09	1.99E+09	-25.1629	-22.0632	3648.226	2608.218	0.085084	0.059548	9.92E-05	2558.096	0.000107	0.003359	2.423676	**2.4144**	**1.414399**	**0.013782**	0.027457	**0.027**	**0.000408**	0.029537	**0.028884**	**0.000413**	**1.3876**	**2.4227**	**0.0272**
					14_pentaF	794405	595666	1.97E+09	1.99E+09	-25.0553	-22.0613	3650.235	2643.111	0.085103	0.06029	9.41E-05	2592.029	0.000102	0.003273	2.387282				0.026056			0.02812					
					15_pentaF	800523	595847	1.97E+09	1.99E+09	-25.122	-22.0611	3649.134	2621.345	0.085105	0.059829	9.73E-05	2570.939	0.000105	0.003379	2.43224				0.026925			0.028996					
		2.48 GHz	2.48	CAV	13_pentaF	588968	309354	2.48E+09	2.5E+09	-50.0299	-31.6813	8315.684	4231.649	0.028378	0.003432	0.000108	1004.994	0.000866	0.002778	2.460885	**2.4709**	**1.470891**	**0.005176**	0.028099	**0.028**	**0.000235**	0.225904	**0.227601**	**0.00087**	**1.4432**	**2.4908**	**0.0288**
					14_pentaF	581318	309418	2.48E+09	2.5E+09	-50.1254	-31.7104	8313.148	4287.034	0.028282	0.003394	0.000105	993.9691	0.000878	0.002811	2.47819				0.027303			0.228781					
					15_pentaF	583660	309468	2.48E+09	2.5E+09	-50.1035	-31.7053	8311.947	4269.906	0.028299	0.003403	0.000106	996.4907	0.000875	0.002802	2.473598				0.027547			0.228117					
		2.97 GHz	2.97	PPR	13_pentaF	1049000	768368	2.97E+09	2.99E+09	-21.5334	-18.3589	4493.278	3120.857	0.132609	0.092013	8.91E-05	3047.994	9.68E-05	0.00301	2.41722	**2.4093**	**1.409323**	**0.013799**	0.027467	**0.027**	**0.000359**	0.029828	**0.029302**	**0.000357**	**1.3824**	**2.4167**	**0.0273**
					14_pentaF	1037310	768542	2.97E+09	2.99E+09	-21.4387	-18.3605	4492.134	3159.771	0.132584	0.093021	8.52E-05	3084.835	9.29E-05	0.002936	2.382474				0.026251			0.028621					
					15_pentaF	1045200	768696	2.97E+09	2.99E+09	-21.5044	-18.3546	4491.7	3133.192	0.132674	0.09232	8.79E-05	3059.225	9.56E-05	0.003033	2.428275				0.027079			0.029457					
		3.98 GHz	3.98	PPR	13_pentaF	1304810	929132	3.98E+09	4E+09	-19.604	-16.0776	5221.993	3449.309	0.174976	0.116589	9.01E-05	3345.426	9.91E-05	0.002788	2.409902	**2.4027**	**1.402715**	**0.014015**	0.0294	**0.029**	**0.000367**	0.032337	**0.031819**	**0.000362**	**1.3738**	**2.4090**	**0.0291**
					14_pentaF	1289740	928781	3.98E+09	4E+09	-19.5079	-16.078	5223.903	3494.981	0.174968	0.117887	8.63E-05	3387.619	9.54E-05	0.00272	2.375659				0.028164			0.031122					
					15_pentaF	1300380	929074	3.98E+09	4E+09	-19.5773	-16.0751	5222.625	3462.38	0.175027	0.116948	8.9E-05	3357.09	9.81E-05	0.002813	2.422585				0.029043			0.031998					
pentaF	P(xyl)3	0.98 GHz	1	PPR	PXyl3 pent	819196	393824.2	9.77E+08	9.86E+08	-36.2164	-29.7488	2592.716	1212.66	0.034339	0.016308	0.000428	1217.569	0.000424	0.003676	2.431266	**2.4389**	**1.438899**	**0.003816**	0.101328	**0.100**	**0.001544**	0.10054	**0.099515**	**0.001591**	**1.3389**	**2.4518**	**0.1026**
					PXyl3 pent	821071.2	394484.5	9.77E+08	9.86E+08	-36.2633	-29.7481	2588.378	1209.747	0.034342	0.016221	0.00043	1210.898	0.000429	0.003705	2.442738				0.101798			0.101612					
					PXyl3 pent	801091.3	393681	9.77E+08	9.86E+08	-36.0323	-29.7396	2593.749	1240.47	0.034376	0.016658	0.000409	1244.085	0.000407	0.003705	2.442692				0.096947			0.096392					
		1.97 GHz	1.97	PPR	PXyl3 pent	1165465	600960	1.97E+09	1.99E+09	-27.6036	-21.5103	3640.219	1772.581	0.090676	0.04496	0.000279	1754.425	0.000285	0.003319	2.401565	**2.4123**	**1.4123**	**0.005371**	0.077399	**0.076**	**0.001138**	0.079017	**0.078323**	**0.001204**	**1.3358**	**2.4202**	**0.0775**
					PXyl3 pent	1168042	601107.2	1.97E+09	1.99E+09	-27.6536	-21.501	3639.713	1768.123	0.090774	0.044702	0.000281	1743.882	0.000289	0.003358	2.41803				0.077793			0.079972					
					PXyl3 pent	1142576	601296.7	1.97E+09	1.99E+09	-27.4426	-21.4984	3638.669	1809.617	0.090801	0.045801	0.000268	1788.815	0.000274	0.003357	2.417303				0.074199			0.07598					
		2.48 GHz	2.48	CAV	PXyl3 pent	945596.7	248687.4	2.48E+09	2.5E+09	-43.5842	-31.684	10343.99	2645.872	0.028368	0.007208	0.000272	2594.385	0.00028	0.002707	2.422312	**2.4222**	**1.422154**	**0.001612**	0.069923	**0.069**	**0.000636**	0.071848	**0.071039**	**0.000716**	**1.3529**	**2.4318**	**0.0706**
					PXyl3 pent	945915.3	248779.8	2.48E+09	2.5E+09	-43.5678	-31.6834	10340.17	2645.032	0.02837	0.007222	0.000272	2599.341	0.000279	0.002702	2.419286				0.069953			0.071659					
					Pxyl3 pent	927313.1	248714.2	2.48E+09	2.5E+09	-43.387	-31.6842	10342.87	2698.477	0.028368	0.007374	0.000265	2654.398	0.000271	0.002712	2.424865				0.068031			0.069611					
		2.97 GHz	2.97	PPR	PXyl3 pent	1405447	762105.7	2.97E+09	2.99E+09	-24.2714	-19.0285	4479.401	2267.346	0.12277	0.067134	0.000209	2298.254	0.000203	0.00297	2.391878	**2.4061**	**1.406069**	**0.007153**	0.064561	**0.064**	**0.000856**	0.062728	**0.062317**	**0.000885**	**1.3420**	**2.4129**	**0.0647**
					PXyl3 pent	1411217	762362.5	2.97E+09	2.99E+09	-24.3239	-19.0473	4476.529	2256.985	0.122504	0.06673	0.000211	2283.413	0.000206	0.003019	2.414728				0.065187			0.063602					
					PXyl3 pent	1384216	762624.7	2.97E+09	2.99E+09	-24.1436	-19.0388	4475.6	2304.485	0.122625	0.06813	0.000202	2334.831	0.000196	0.003012	2.411601				0.062364			0.06062					
		3.98 GHz	3.98	PPR	PXyl3 pent	1651843	928568.3	3.98E+09	4E+09	-21.9151	-16.3015	5197.118	2643.293	0.170523	0.089352	0.000177	2570.2	0.000188	0.002755	2.385785	**2.4042**	**1.404231**	**0.009372**	0.058574	**0.058**	**0.000686**	0.062125	**0.061994**	**0.000761**	**1.3459**	**2.4108**	**0.0590**
					PXyl3 pent	1661866	928228.1	3.98E+09	4E+09	-21.9836	-16.349	5193.194	2625.17	0.169593	0.088651	0.00018	2548.073	0.000192	0.002816	2.416341				0.059436			0.06324					
					PXyl3 pent	1633837	928016	3.98E+09	4E+09	-21.8216	-16.3383	5195.682	2675.134	0.169802	0.090319	0.000173	2600.789	0.000184	0.002804	2.410567				0.057088			0.060615					
pentaF	P(mes)3	0.98 GHz	1	PPR	PMes3 + p	643534.6	392123.6	9.77E+08	9.86E+08	-34.2003	-29.7546	2603.97	1550.571	0.03432	0.020571	0.000249	1550.091	0.000249	0.003583	2.423082	**2.4397**	**1.43966**	**0.008497**	0.062177	**0.061**	**0.000446**	0.062227	**0.061607**	**0.000434**	**1.3782**	**2.4527**	**0.0631**
					PMes3 + p	637496.1	391837.1	9.77E+08	9.86E+08	-34.1231	-29.7584	2605.84	1565.466	0.034305	0.020755	0.000243	1564.219	0.000243	0.003637	2.444709				0.060645			0.060772					
					PMes3 + p	641145.1	392238	9.77E+08	9.86E+08	-34.1789	-29.7585	2603.187	1556.32	0.034304	0.020622	0.000247	1554.004	0.000248	0.003654	2.451189				0.061582			0.061821					
		1.97 GHz	1.97	PPR	PMes3 + p	942400.9	596870.7	1.99E+09	2E+09	-25.8999	-21.5946	3681.698	2228.757	0.089833	0.054723	0.000167	2203.647	0.000172	0.00302	2.440869	**2.4278**	**1.427801**	**0.011694**	0.046029	**0.046**	**0.000308**	0.04744	**0.047106**	**0.000294**	**1.3822**	**2.4385**	**0.0466**
					PMes3 + p	935225.4	596859	1.99E+09	2E+09	-25.8394	-21.5949	3681.751	2246.939	0.089829	0.055105	0.000163	2219.963	0.000169	0.002944	2.40447				0.045027			0.046519					
					PMes3 + p	941075.1	596804.3	1.99E+09	2E+09	-25.8946	-21.5963	3682.052	2231.989	0.089814	0.054756	0.000166	2205.08	0.000172	0.003014	2.438063				0.04585			0.047358					
		2.48 GHz	2.48	CAV	PMes3 + p	760734.2	314975.3	2.48E+09	2.5E+09	-27.3968	-19.6551	0.046485	0.11359	3423.795	8951.012	0.000172	0.002769	3424.004	0.000172	2.447464	**2.4448**	**1.444786**	**0.001821**	0.044895	**0.045**	**5.37E-05**	0.04489	**0.044952**	**5.37E-05**	**1.3999**	**2.4589**	**0.0461**
					PMes3 + p	759559.4	314873.5	2.48E+09	2.5E+09	-27.3961	-19.6708	0.046489	0.113294	3429.145	8950.875	0.000171	0.002758	3424.295	0.000172	2.44131				0.044776			0.044884					
					PMes3 + p	761288.8	315194	2.48E+09	2.5E+09	-27.4174	-19.67	0.046375	0.113467	3420.917	8943.5	0.000172	0.002766	3415.479	0.000173	2.445585				0.044959			0.045081					
		2.97 GHz	2.97	PPR	PMes3 + p	1179323	759543.3	2.97E+09	2.99E+09	-22.5933	-18.884	4505.126	2744.274	0.124847	0.081454	0.000133	2780.876	0.000128	0.002961	2.415278	**2.4327**	**1.432696**	**0.008806**	0.040138	**0.040**	**0.000207**	0.038687	**0.038432**	**0.000194**	**1.3928**	**2.4444**	**0.0408**
					PMes3 + p	1172550	759668.2	2.97E+09	2.99E+09	-22.5466	-18.8836	4504.419	2761.309	0.124853	0.081894	0.00013	2797.211	0.000126	0.003011	2.439149				0.039458			0.038052					
					PMes3 + p	1177797	759707.5	2.97E+09	2.99E+09	-22.5836	-18.8836	4504.188	2747.937	0.124853	0.081545	0.000132	2784.234	0.000127	0.003021	2.44366				0.039991			0.038556					
		3.98 GHz	3.98	PPR	PMes3 + p	1422942	916972.3	3.98E+09	4E+09	-20.9649	-16.5367	5234.231	3103.825	0.165992	0.099696	0.000123	3015.753	0.000133	0.002751	2.414838	**2.4313**	**1.431333**	**0.008262**	0.039274	**0.039**	**0.000131**	0.042277	**0.042139**	**0.000117**	**1.3922**	**2.4427**	**0.0400**
					PMes3 + p	1417352	916965.3	3.98E+09	4E+09	-20.9375	-16.5374	5234.194	3117.015	0.165981	0.100012	0.000122	3026.362	0.000131	0.002798	2.438723				0.038839			0.041906					
					PMes3 + p	1421419	916842.1	3.98E+09	4E+09	-20.9618	-16.5373	5234.902	3107.12	0.165981	0.099732	0.000123	3016.967	0.000132	0.002801	2.440437				0.039165			0.042234					
None	Toluene	0.98 GHz	1	PPR	Tol	421602.2	396713.7	9.78E+08	9.86E+08	-30.4976	-29.7931	2573.761	2394.001	0.034168	0.031506	1.69E-05	2381.829	1.9E-05	0.003541	2.389783	**2.3800**	**1.38**	**0.005992**	0.004267	**0.004**	**0.000136**	0.004806	**0.004793**	**1.9E-05**	**1.3756**	**2.3829**	**0.0044**
					Tol	421726.3	396465.1	9.78E+08	9.86E+08	-30.4984	-29.7931	2575.376	2393.343	0.034168	0.031503	1.7E-05	2381.589	1.91E-05	0.003519	2.381101				0.004296			0.004817					
					Tol	423321.9	370470.6	9.78E+08	9.86E+08	-30.4936	-29.5096	2759.317	2384.438	0.035302	0.031521	1.86E-05	2382.973	1.88E-05	0.003488	2.369115				0.00469			0.004755					
		1.97 GHz	1.97	PPR	Tol	679572.5	602443.2	1.99E+09	2E+09	-23.0716	-21.662	3644.85	3161.575	0.089138	0.075785	3.15E-05	3111.704	3.65E-05	0.002894	2.380911	**2.3800**	**1.38**	**0.009069**	0.008923	**0.009**	**2.17E-05**	0.01036	**0.010334**	**5.93E-05**	**1.3710**	**2.3829**	**0.0090**
					Tol	679854.6	602351.1	1.99E+09	2E+09	-23.0769	-21.6685	3645.129	3160.218	0.089071	0.075738	3.16E-05	3109.633	3.67E-05	0.002858	2.363856				0.008961			0.010421					
					Tol	680200.1	602457.9	1.99E+09	2E+09	-23.0593	-21.6622	3644.748	3158.931	0.089136	0.075892	3.17E-05	3116.48	3.6E-05	0.002924	2.395233				0.008998			0.010221					
		2.48 GHz	2.48	CAV	Tol	436498	314281.3	2.48E+09	2.5E+09	-22.7796	-19.7776	0.0791	0.111659	6178.791	8950.896	4.08E-05	0.002536	6120.916	4.23E-05	2.377587	**2.3800**	**1.38**	**0.004842**	0.011075	**0.011**	**9.94E-05**	0.011491	**0.011468**	**9.94E-05**	**1.3688**	**2.3829**	**0.0112**
					Tol	436445.9	314465.6	2.48E+09	2.5E+09	-22.7534	-19.7692	0.079339	0.111508	6181.187	8944.077	4.07E-05	0.002527	6141.034	4.18E-05	2.373084				0.011058			0.011345					
					Tol	439300.8	314632	2.48E+09	2.5E+09	-22.7936	-19.7831	0.078973	0.111578	6138.391	8940.015	4.18E-05	0.002557	6110.251	4.26E-05	2.389329				0.011364			0.011568					
		2.97 GHz	2.97	PPR	Tol	915554.8	764718.9	2.97E+09	2.99E+09	-20.5886	-19.0393	4463.222	3618.124	0.122634	0.1026	4.33E-05	3642.356	4.14E-05	0.002946	2.38926	**2.3800**	**1.38**	**0.007348**	0.013421	**0.013**	**3.14E-05**	0.012851	**0.012831**	**5.45E-05**	**1.3666**	**2.3829**	**0.0134**
					Tol	915983.8	764722.7	2.97E+09	2.99E+09	-20.5944	-19.0442	4462.849	3616.182	0.122566	0.102531	4.34E-05	3639.627	4.16E-05	0.002937	2.385253				0.013467			0.012914					
					Tol	915069.9	764767	2.97E+09	2.99E+09	-20.5774	-19.0424	4462.715	3620.756	0.12259	0.102732	4.31E-05	3647.576	4.1E-05	0.002895	2.365487				0.013359			0.012729					
		3.98 GHz	3.98	PPR	Tol	1166541	930199	3.98E+09	4E+09	-18.7987	-16.3319	5184.221	3908.492	0.169953	0.127935	5.47E-05	3805.083	6.17E-05	0.002744	2.388355	**2.3800**	**1.38**	**0.0083**	0.017953	**0.018**	**5.99E-05**	0.020234	**0.020224**	**0.000108**	**1.3621**	**2.3829**	**0.0179**
					Tol	1167239	930196.4	3.98E+09	4E+09	-18.8138	-16.3424	5182.95	3905.161	0.169747	0.127712	5.49E-05	3797.499	6.22E-05	0.002743	2.388245				0.018024			0.020406					
					Tol	1165105	930200.9	3.98E+09	4E+09	-18.7809	-16.3339	5183.961	3914.682	0.169914	0.128197	5.43E-05	3814.041	6.11E-05	0.002694	2.3634				0.01782			0.020032					

S3.5 Lewis acidity comparison with $PMes_3$ (Figure 2)

S3.5.1 MDS measurement

A set of Lewis Acid with trimesitylphosphine (**LA3**) in toluene solution (0.1 M) were measured in a range of microwave frequencies and resonator cavity, the preparation and measurement of samples following the same procedure of standard measurement. Data of ε_2 measurement taken in PPR(1 GHz) are presented as it is as discussed in S3.5.1.

Acid	Acid	Frequency	Resonator	Sample	BXs	BX0	FXs	FX0	LXs	LX0	Q0	Qs	S0	S1	Δ(1/Q)
				PMes3 + B	453194.8	391421.5	9.77E+08	9.86E+08	-31.1556	-29.7589	2608.524	2221.376	0.034303	0.029207	5.42E-05
BPh3	P(mes)3	0.98 GHz	PPR	PMes3 + B	452282	392046.7	9.77E+08	9.86E+08	-31.1654	-29.7608	2604.352	2225.707	0.034295	0.029174	5.34E-05
				PMes3 + B	452059.2	391699.5	9.77E+08	9.86E+08	-31.1489	-29.7565	2606.727	2227.028	0.034312	0.02923	5.31E-05
				11F4	450114.8	396909.8	9.77E+08	9.86E+08	-30.1041	-28.7158	2584.83	2245.531	0.038676	0.032963	4.6E-05
4-F	P(mes)3	0.98 GHz	PPR	12	450990.9	394913.9	9.77E+08	9.86E+08	-30.1054	-28.7103	2597.959	2241.153	0.038701	0.032958	4.69E-05
				13	451395.4	396199	9.77E+08	9.86E+08	-30.1199	-28.7159	2589.464	2238.831	0.038676	0.032903	4.74E-05
				21F26	451718.8	396174.4	9.77E+08	9.86E+08	-30.1248	-28.7186	2589.591	2237.404	0.038664	0.032885	4.76E-05
26diF	P(mes)3	0.98 GHz	PPR	22	450985.3	396622.4	9.78E+08	9.86E+08	-30.1159	-28.7177	2586.677	2241.373	0.038668	0.032919	4.69E-05
				23	451022.9	395945.1	9.78E+08	9.86E+08	-30.112	-28.7132	2591.154	2241.401	0.038688	0.032933	4.69E-05
				Pmes3 1	501197.9	394722.5	9.77E+08	9.86E+08	-31.965	-29.7408	2586.888	2003.122	0.034371	0.026606	0.000101
246triF	P(mes)3	0.98 GHz	PPR	Pmes3 2	505948.7	393913.1	9.77E+08	9.86E+08	-32.0387	-29.741	2592.201	1983.852	0.03437	0.026381	0.000106
				Pmes3 3	500184.2	394359.2	9.77E+08	9.86E+08	-31.9592	-29.7408	2589.27	2007.213	0.034371	0.026624	0.0001
				31F345	628213	396808.9	9.77E+08	9.86E+08	-32.9771	-28.7185	2585.45	1593.524	0.038664	0.02368	0.000228
345triF	P(mes)3	0.98 GHz	PPR	32R	622610.8	395958.9	9.78E+08	9.86E+08	-32.9007	-28.7152	2591.039	1608.475	0.038679	0.023889	0.000222
				33R	624607.1	396545.7	9.77E+08	9.86E+08	-32.9363	-28.7211	2587.13	1603.011	0.038653	0.023792	0.000225
				les3 + BCF	643534.6	392123.6	9.77E+08	9.86E+08	-34.2003	-29.7546	2603.97	1550.571	0.03432	0.020571	0.000249
pentaF	P(mes)3	0.98 GHz	PPR	les3 + BCF	637496.1	391837.1	9.77E+08	9.86E+08	-34.1231	-29.7584	2605.84	1565.466	0.034305	0.020755	0.000243
				les3 + BCF	641145.1	392238	9.77E+08	9.86E+08	-34.1789	-29.7585	2603.187	1556.32	0.034304	0.020622	0.000247

Sample	Qs IL	Δ(1/Q) IL	Δf/f	ε1	ε1 avg	ε1 r	ε1 err	ε2	ε2 avg	ε2 err	ε2 IL	ε2 IL avg	ε2 IL err	ε1-1-ε2	1 correcte	2corrected
PMes3 + B	2220.435	5.44E-05	0.003589	2.425697	**2.4291**	**1.4291**	**0.0052**	0.013545	**0.0134**	**0.0001**	0.013593	**0.013606**	**6.42E-05**	**1.41572**	**2.4401**	**0.0137**
PMes3 + B	2217.855	5.5E-05	0.003624	2.439298				0.013326			0.013723					
PMes3 + B	2222.217	5.41E-05	0.003581	2.422288				0.01326			0.013502					
11F4	2218.436	5.15E-05	0.003865	2.413537	**2.4241**	**1.4241**	**0.0102**	0.011648	**0.0118**	**0.0001**	0.013024	**0.013101**	**6.89E-05**	**1.41231**	**2.4342**	**0.0121**
12	2218.1	5.15E-05	0.003867	2.414343				0.011868			0.013041					
13	2214.268	5.23E-05	0.00395	2.44454				0.011985			0.013239					
21F26	2212.968	5.26E-05	0.003853	2.408893	**2.3719**	**1.3719**	**0.0204**	0.012057	**0.0119**	**0.0001**	0.013306	**0.013207**	**5.16E-05**	**0.35997**	**1.7878**	**0.0092**
22	2215.33	5.21E-05	0.003741	2.368096				0.011857			0.013184					
23	2216.348	5.19E-05	0.003661	2.338703				0.011855			0.013132					
Pmes3 1	1994.355	0.000104	0.003625	2.431209	**2.4317**	**1.4317**	**0.0003**	0.025532	**0.0259**	**0.0005**	0.026085	**0.026425**	**0.000384**	**1.40585**	**2.4432**	**0.0264**
Pmes3 2	1977.047	0.000108	0.003626	2.431648				0.026754			0.027191					
Pmes3 3	1995.72	0.000103	0.003627	2.432258				0.025276			0.025999					
31F345	1578.502	0.000234	0.003928	2.436612	**2.4085**	**1.4085**	**0.0176**	0.057753	**0.0569**	**0.0004**	0.059265	**0.058528**	**0.000416**	**1.35160**	**2.4158**	**0.0576**
32R	1592.802	0.000229	0.003763	2.376053				0.056278			0.057825					
33R	1586.133	0.000231	0.003864	2.412983				0.056814			0.058493					
les3 + BCF	1550.091	0.000249	0.003583	2.423082	**2.4397**	**1.4397**	**0.0085**	0.062177	**0.0615**	**0.0004**	0.062227	**0.061607**	**0.000434**	**1.37819**	**2.4527**	**0.0631**
les3 + BCF	1564.219	0.000243	0.003637	2.444709				0.060645			0.060772					
les3 + BCF	1554.004	0.000248	0.003654	2.451189				0.061582			0.061821					

S3.5.2 NMR measurement and water influence

Under glove box nitrogen atmosphere, in a glass vial, $PMes_3$, and a range of Lewis acid (0.02 mmol) were dissolved in $CDCl_3$ (0.7 ml). The solution was transferred into an air tight NMR tube equipped with Young valve. ^{31}P NMR of the Lewis pairs are recorded. No significant ^{31}P NMR shift were observed as discussed.

A control experiment was made with the (**pentaF**, **LA3**) with intended addition of trace of water to compare with MDS measurement and check the water contains of synthesised lewis acid. The presence of trace water in fluorinated triphenyl boranes shows a significant downfield chemical shift in ^{31}P NMR.

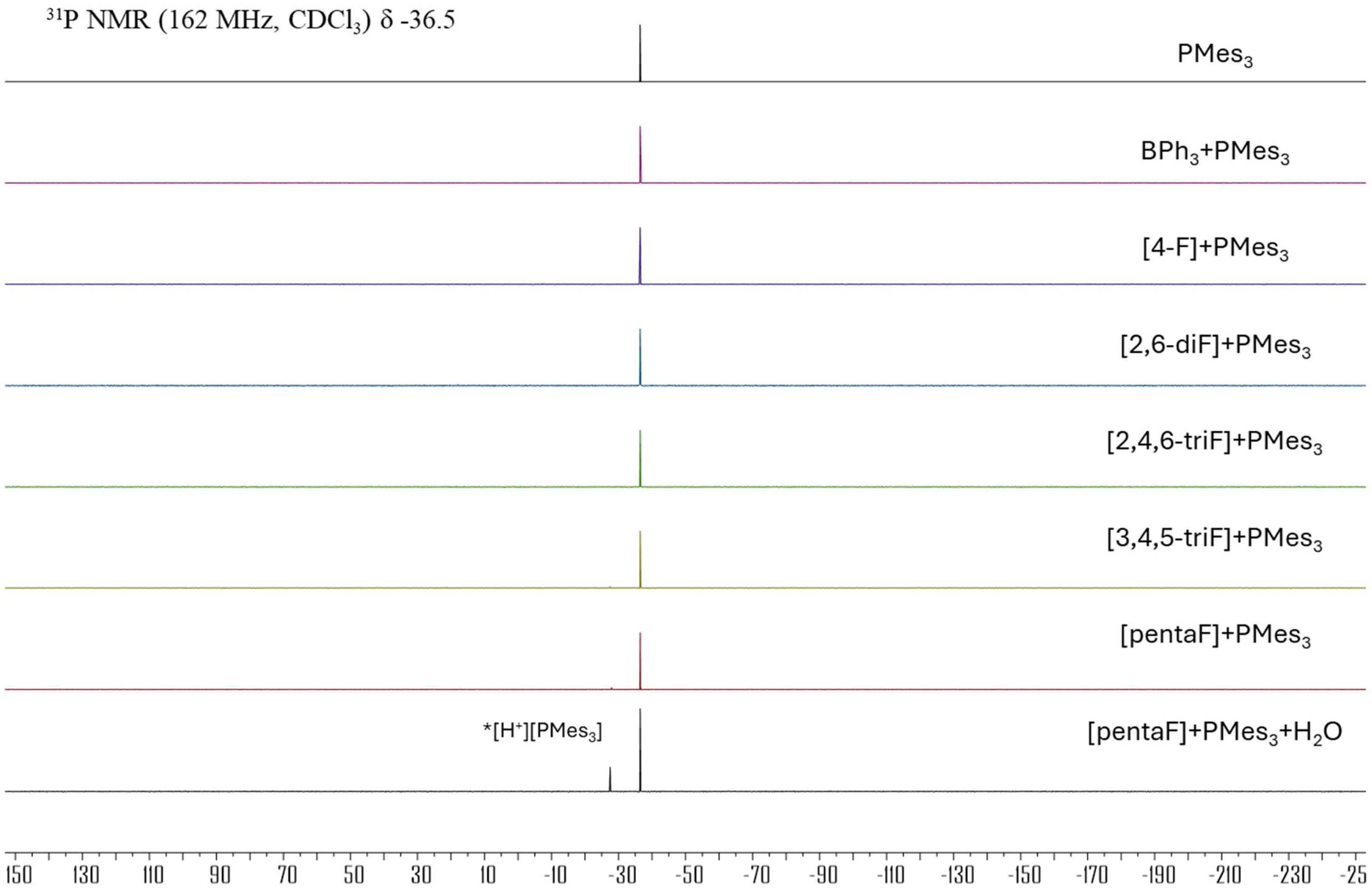

Figure S3.5.2 ^{31}P NMR of $PMes_3$ with a range of fluorinated triaryl boranes Lewis acid

S3.6 Microwave measurement of Lewis pairs hydrogen activation (Table 1B)

Under nitrogen atmosphere in glovebox, Lewis acid (0.15 mmol, 1 eq.), Lewis base (0.15 mmol, 1 eq.), were dissolved in a schlenk tube with dry toluene (1.5 ml). The Schlenk tube were sealed and connected to a hydrogen/vacuum Schlenk line. The tube was freeze-pump-thaw 3 times with liquid nitrogen temperature and r.t. three times and saturated with hydrogen gas. The Lewis pairs were allowed to be stirred under 1 bar hydrogen atmosphere 30 minutes under room temperature. The resulting 0.1 M Lewis pair solution was transferred into a FEP tube (OD 5.00+/-0.10mm, >7 cm long) and sealed with PTFE stopper in a Schlenk tube under nitrogen. The tubes were taken to out and the microwave dielectric spectrum were measured under atmosphere.

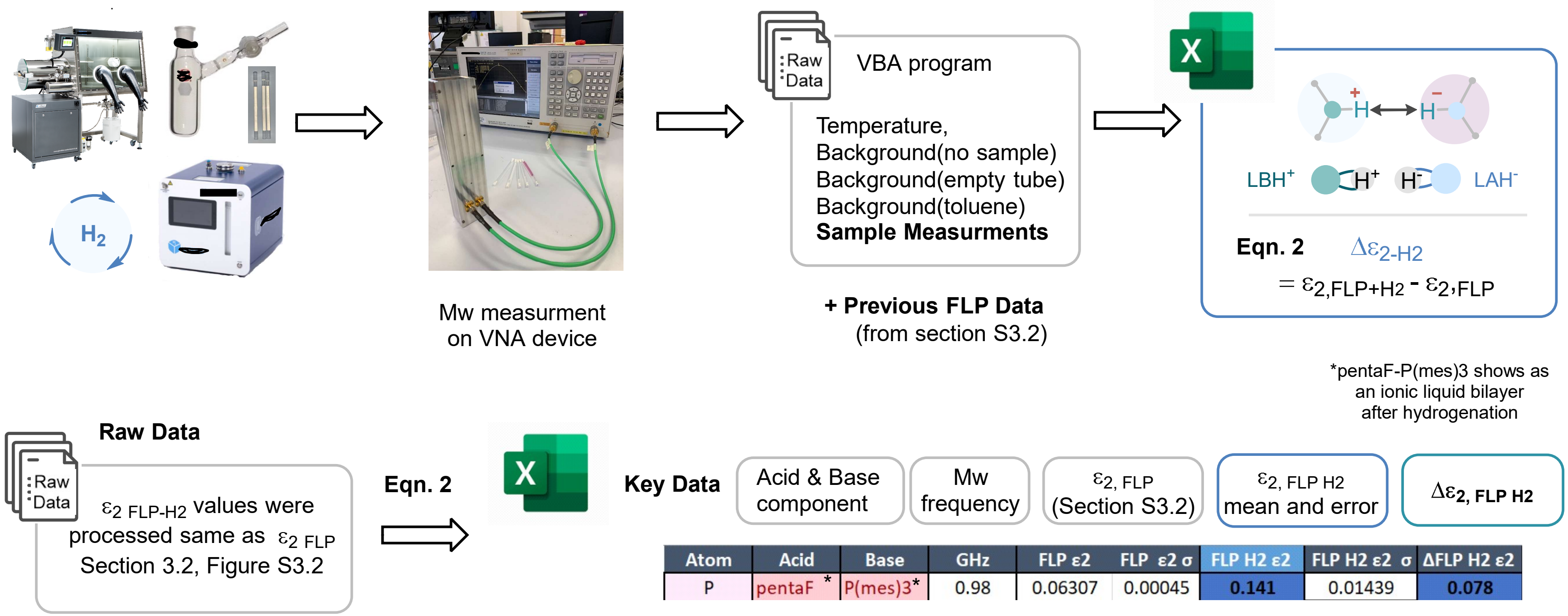

Atom	Acid	Base	GHz	FLP ε2	FLP ε2 σ	FLP H2 ε2	FLP H2 ε2 σ	ΔFLP H2 ε2
P	pentaF *	P(mes)3*	0.98	0.06307	0.00045	**0.141**	0.01439	**0.078**

Figure S3.6 Flow chart of $\Delta\varepsilon_{2\text{-}H2}$ measurement and annotated example of FLP-H_2 measurement

Key result of Lewis pairs hydrogen activation (Table 1B)

Atom	Acid	Base	GHz	FLP ε2	FLP ε2 σ	FLP H2 ε2	FLP H2 ε2 σ	ΔFLP H2 ε2
P	BPh3	PPh3	0.98	0.00699	0.00005	**0.015**	0.00025	**0.008**
P	BPh3	P(o-tol)3	0.98	0.00564	0.00008	**0.007**	0.00017	**0.001**
P	BPh3	P(xyl)3	0.98	0.00934	0.00020	**0.012**	0.00074	**0.003**
P	BPh3	P(mes)3	0.98	0.01366	0.00009	**0.018**	0.00077	**0.004**
P	246triF	PPh3	0.98	0.01361	0.00019	**0.015**	0.00063	**0.001**
P	246triF	P(o-tol)3	0.98	0.01078	0.00020	**0.013**	0.00072	**0.002**
P	246triF	P(xyl)3	0.98	0.01847	0.00017	**0.050**	0.00307	**0.032**
P	246triF	P(mes)3	0.98	0.02643	0.00046	**0.060**	0.00151	**0.033**
P	pentaF	PPh3	0.98	0.04440	0.00034	**0.056**	0.00183	**0.012**
P	pentaF	P(o-tol)3	0.98	0.02990	0.00055	**0.086**	0.00805	**0.056**
P	pentaF	P(xyl)3	0.98	0.10259	0.00154	**0.123**	0.00212	**0.020**
P	pentaF	P(mes)3	0.98	0.06307	0.00045	**0.141**	0.01439	**0.078**
O	BPh3	Et2O	0.98	0.00418	0.00003	**0.007**	0.00012	**0.003**
O	BPh3	CPME	0.98	0.00475	0.00007	**0.006**	0.00012	**0.001**
O	BPh3	TBME	0.98	0.00576	0.00014	**0.009**	0.00021	**0.003**
O	BPh3	Eucalypto	0.98	0.00598	0.00008	**0.008**	0.00007	**0.002**
O	246triF	Et2O	0.98	0.00989	0.00012	**0.016**	0.00013	**0.006**
O	246triF	CPME	0.98	0.01005	0.00033	**0.027**	0.00052	**0.017**
O	246triF	TBME	0.98	0.00994	0.00010	**0.032**	0.00090	**0.022**
O	246triF	Eucalypto	0.98	0.01114	0.00046	**0.029**	0.00022	**0.018**
O	pentaF	Et2O	0.98	0.15096	0.00415	**0.255**	0.00185	**0.104**
O	pentaF	CPME	0.98	0.07180	0.00095	**0.193**	0.00112	**0.121**
O	pentaF	TBME	0.98	0.23285	0.00194	**0.258**	0.00063	**0.025**
O	pentaF	Eucalypto	0.98	0.04884	0.00012	**0.153**	0.00218	**0.104**
						*	Ionic liquid	precipitatior

Raw data of Lewis pairs hydrogen activation (Table 1B)

FLP H2

Acid	Base	Frequency	Resonator	Sample	BXs	BX0	FXs	FX0	LXs	LX0	Q0	Qs	S0	S1	Δ(1/Q)	Qs IL	Δ(1/Q) IL	Δf/f	ε1	ε1 avg	ε1r	ε1 err	ε2	ε2 avg	ε2 err	ε2 IL	ε2 IL avg	ε2 IL err	ε1-1-ε2	ε1 correcte	ε2correcte
BPH3	PPh3	0.98 GHz	PPR	10-PPh3_E	458167.6	393566.1	9.77E+08	9.86E+08	-30.9991	-29.4901	2597.641	2198.804	0.035378	0.029736	5.72E-05	2183.492	6.03E-05	0.003628	2.452577	**2.4179**	**1.4179**	**0.0175**	0.0152	**0.0149**	**0.0003**	0.0161	**0.0158**	**0.0003**	**1.4029**	**2.4268**	**0.0152**
				11-PPh3_E	457588.8	393147.6	9.78E+08	9.86E+08	-30.9926	-29.4851	2600.46	2201.905	0.035398	0.029758	5.65E-05	2185.183	6E-05	0.003507	2.403923				0.0151			0.0160					
				12-PPh3_E	455209.8	393543.6	9.78E+08	9.86E+08	-30.9352	-29.4895	2597.796	2213.9	0.03538	0.029955	5.41E-05	2200.116	5.69E-05	0.00349	2.397067				0.0144			0.0152					
BPH3	P(o-tol)3	0.98 GHz	PPR	13-P(o-tol	425493.3	393682	9.78E+08	9.86E+08	-30.3795	-29.4909	2596.866	2373.163	0.035374	0.031935	2.37E-05	2350.284	2.78E-05	0.003573	2.430505	**2.4032**	**1.4032**	**0.0143**	0.0063	**0.0066**	**0.0002**	0.0074	**0.0075**	**0.0001**	**1.3965**	**2.4095**	**0.0067**
				14-P(o-tol	427786.8	393706.5	9.78E+08	9.86E+08	-30.3992	-29.4888	2596.727	2360.55	0.035383	0.031862	2.6E-05	2344.786	2.88E-05	0.003452	2.382146				0.0069			0.0077					
				15-P(o-tol	426763.3	393210.8	9.78E+08	9.86E+08	-30.3824	-29.4857	2600.036	2366.274	0.035396	0.031924	2.5E-05	2349.457	2.8E-05	0.003489	2.396811				0.0067			0.0075					
BPH3	P(xyl)3	0.98 GHz	PPR	16-P(xyl)3	443873.9	393522.3	9.78E+08	9.86E+08	-30.7143	-29.4894	2597.936	2272.338	0.03538	0.030727	4.24E-05	2258.592	4.51E-05	0.00345	2.381233	**2.4098**	**1.4098**	**0.0324**	0.0113	**0.0120**	**0.0007**	0.0120	**0.0128**	**0.0008**	**1.3978**	**2.4173**	**0.0121**
				17-P(xyl)3	443213.5	393486.4	9.77E+08	9.86E+08	-30.7178	-29.4902	2598.164	2275.163	0.035377	0.030715	4.19E-05	2257.65	4.53E-05	0.003683	2.474528				0.0112			0.0121					
				18-P(mes)	451717.9	393575.3	9.78E+08	9.86E+08	-30.8802	-29.4888	2597.594	2231.584	0.035383	0.030146	5.05E-05	2214.539	5.39E-05	0.003431	2.373661				0.0135			0.0144					
BPH3	P(mes)3	0.98 GHz	PPR	19-P(mes)	469977.9	393472.6	9.77E+08	9.86E+08	-31.2025	-29.4864	2598.298	2142.083	0.035393	0.029047	6.92E-05	2131.441	7.15E-05	0.003604	2.442813	**2.4441**	**1.4441**	**0.0150**	0.0185	**0.0171**	**0.0008**	0.0191	**0.0181**	**0.0005**	**1.4270**	**2.4581**	**0.0175**
				20-P(mes)	460159.1	393877.7	9.78E+08	9.86E+08	-31.0776	-29.4906	2595.58	2188.87	0.035376	0.029468	5.92E-05	2163.248	6.46E-05	0.003544	2.418797				0.0158			0.0172					
				21-P(mes)	464325.9	393536.2	9.77E+08	9.86E+08	-31.1191	-29.488	2597.86	2168.632	0.035386	0.029328	6.35E-05	2152.639	6.69E-05	0.003673	2.470662				0.0169			0.0178					
246triF	PPh3	0.98 GHz	PPR	10-PPh3_E	467253.8	394903.3	9.78E+08	9.86E+08	-31.1598	-29.5128	2588.587	2155.022	0.035285	0.029191	6.79E-05	2155.321	6.78E-05	0.003528	2.404133	**2.3568**	**1.3568**	**0.0468**	0.0158	**0.0145**	**0.0006**	0.0157	**0.0147**	**0.0006**	**1.3423**	**2.3568**	**0.0143**
				11-PPh3_E	460119.2	394682	9.78E+08	9.86E+08	-31.0735	-29.5119	2590.048	2189.103	0.035289	0.029482	6.07E-05	2177.509	6.31E-05	0.003525	2.402883				0.0141			0.0146					
				12-PPh3_E	458706.5	395112.2	9.78E+08	9.86E+08	-30.9965	-29.5131	2587.215	2197.211	0.035284	0.029745	5.9E-05	2197.487	5.89E-05	0.003174	2.263252				0.0137			0.0137					
246triF	P(o-tol)3	0.98 GHz	PPR	13-P(o-tol	448677.3	394769.6	9.78E+08	9.86E+08	-30.8034	-29.5111	2589.482	2247.272	0.035292	0.030414	4.88E-05	2248.441	4.86E-05	0.003441	2.369578	**2.4114**	**1.4114**	**0.0209**	0.0113	**0.0126**	**0.0007**	0.0113	**0.0129**	**0.0008**	**1.3989**	**2.4192**	**0.0127**
				14-P(o-tol	459090.4	394981.9	9.77E+08	9.86E+08	-30.9974	-29.5128	2588.072	2194.435	0.035286	0.029742	5.96E-05	2197.242	5.9E-05	0.003598	2.431693				0.0138			0.0137					
				15-P(o-tol	453744.2	394798.8	9.77E+08	9.86E+08	-31.0049	-29.512	2589.282	2220.224	0.035289	0.029716	5.43E-05	2195.285	5.94E-05	0.003601	2.433057				0.0126			0.0138					
246triF	P(xyl)3	0.98 GHz	PPR	16-P(xyl)3	610384.3	394843.8	9.77E+08	9.86E+08	-33.4749	-29.5133	2588.972	1638.07	0.035283	0.022361	0.000214	1639.497	0.000214	0.003583	2.426052	**2.4384**	**1.4384**	**0.0110**	0.0497	**0.0490**	**0.0031**	0.0496	**0.0490**	**0.0030**	**1.3894**	**2.4513**	**0.0503**
				17-P(-xyl)3	583647.3	394974.7	9.77E+08	9.86E+08	-33.0945	-29.5142	2588.105	1714.855	0.03528	0.023362	0.000187	1714.663	0.000187	0.00359	2.428896				0.0434			0.0434					
				18-P(xyl)3	628084.1	394631.4	9.77E+08	9.86E+08	-33.7277	-29.5104	2590.398	1590.727	0.035295	0.021719	0.000232	1591.422	0.000232	0.00367	2.460376				0.0539			0.0539					
246triF	P(mes)3	0.98 GHz	PPR	19-P(mes)	639065.5	394828.8	9.77E+08	9.86E+08	-33.8857	-29.5144	2589.059	1562.824	0.035279	0.021328	0.000244	1562.114	0.000244	0.003634	2.446033	**2.4030**	**1.4030**	**0.0341**	0.0565	**0.0593**	**0.0015**	0.0566	**0.0593**	**0.0015**	**1.3437**	**2.4094**	**0.0598**
				20-P(mes)	651583.3	394876.6	9.78E+08	9.86E+08	-34.0514	-29.5153	2588.736	1532.595	0.035275	0.020925	0.000256	1531.968	0.000257	0.003356	2.335652				0.0595			0.0595					
				21-P(mes)	661135.5	395074.4	9.77E+08	9.86E+08	-34.1714	-29.5161	2587.431	1509.66	0.035272	0.020638	0.000266	1510.506	0.000266	0.003587	2.427353				0.0618			0.0617					
pentaF	PPh3	0.98 GHz	PPR	10-PPh3_p	616831.9	394095.3	9.77E+08	9.86E+08	-33.5822	-29.4989	2594.055	1620.462	0.035342	0.022086	0.000221	1615.717	0.000223	0.003603	2.471535	**2.4489**	**1.4489**	**0.0202**	0.0523	**0.0544**	**0.0018**	0.0528	**0.0548**	**0.0018**	**1.3945**	**2.4639**	**0.0560**
				11-PPh3_p	640391.8	393759.8	9.77E+08	9.86E+08	-33.9015	-29.499	2596.264	1559.593	0.035342	0.021289	0.000245	1556.139	0.000246	0.003591	2.466611				0.0580			0.0584					
				12-PPh3_p	618991.1	393835.1	9.78E+08	9.86E+08	-33.6116	-29.4973	2595.788	1614.936	0.035349	0.022012	0.000223	1610.134	0.000225	0.003448	2.408526				0.0528			0.0533					
pentaF	P(o-tol)3	0.98 GHz	PPR	12-P(o-tol	677431.2	394017.9	9.77E+08	9.86E+08	-34.3976	-29.4988	2594.567	1472.47	0.035343	0.020107	0.000283	1467.968	0.000285	0.003639	2.486521	**2.5048**	**1.5048**	**0.0107**	0.0670	**0.0815**	**0.0081**	0.0675	**0.0820**	**0.0081**	**1.4233**	**2.5334**	**0.0861**
				14-P(o-tol	741481	393902.6	9.77E+08	9.86E+08	-35.1797	-29.4962	2595.355	1342.782	0.035353	0.018376	0.000348	1339.199	0.00035	0.00373	2.523418				0.0826			0.0830					
				15-P(o-tol	792162.7	393683.7	9.77E+08	9.86E+08	-35.761	-29.4942	2596.82	1255.41	0.035361	0.017187	0.0004	1251.003	0.000403	0.003684	2.50453				0.0949			0.0955					
pentaF	P(xyl)3	0.98 GHz	PPR	16-P(xyl)3	877761.8	393898.1	9.77E+08	9.86E+08	-36.6232	-29.4977	2595.369	1131.143	0.035347	0.015562	0.000488	1130.908	0.000488	0.003657	2.493786	**2.4834**	**1.4834**	**0.0104**	0.1156	**0.1176**	**0.0021**	0.1156	**0.1177**	**0.0021**	**1.3659**	**2.5064**	**0.1230**
				17-P(xyl)3	903484.7	393899.5	9.77E+08	9.86E+08	-36.8791	-29.4973	2595.365	1098.519	0.035349	0.015111	0.000514	1097.572	0.000515	0.003581	2.462602				0.1218			0.1220					
				18-P(xyl)3	876613.5	394109	9.77E+08	9.86E+08	-36.6186	-29.5001	2593.955	1132.634	0.035337	0.015571	0.000487	1131.525	0.000487	0.003657	2.493908				0.1153			0.1155					
pentaF	P(mes)3	0.98 GHz	PPR	19-P(mes)	900409.6	394201.1	9.77E+08	9.86E+08	-36.8745	-29.5001	2593.349	1102.216	0.035337	0.015119	0.000511	1098.171	0.000514	0.003638	2.485917	**2.4331**	**1.4331**	**0.0286**	0.1211	**0.1375**	**0.0144**	0.1219	**0.1382**	**0.0143**	**1.2955**	**2.4448**	**0.1407**
				20-P(mes)	1086688	394161.3	9.78E+08	9.86E+08	-38.4935	-29.5015	2593.596	911.1176	0.035332	0.012548	0.000701	909.0483	0.000704	0.003398	2.387717				0.1662			0.1668					
				21-P(mes)	917689.3	393998.5	9.78E+08	9.86E+08	-37.0367	-29.501	2594.672	1081.315	0.035334	0.014839	0.000528	1077.542	0.000532	0.00349	2.425543				0.1253			0.1260					

FLP H2

Acid	Base	Frequency	Resonator	Sample	BXs	BX0	FXs	FX0	LXs	LX0	Q0	Qs	S0	S1	Δ(1/Q)	Qs IL	Δ(1/Q) IL	Δf/f	ε1	ε1avg	ε1 -1	ε1 err	ε2	ε2 avg	ε2 err	ε2 IL	ε2 IL avg	ε2 IL err	ε1-1-ε2	ε1 corrected	ε2corrected
BPH3	Et2O	0.98 GHz	PPR	51	427456.2	393694.2	9.78E+08	9.86E+08	-29.6159	-28.635	2606.945	2369.882	0.039038	0.034869	2.62E-05	2348.753	3E-05	0.00334	2.286127	**2.3523**	**1.3523**	**0.0339**	0.0066	**0.0068**	**0.0001**	0.0076	0.0079	0.0002	**1.3454**	**2.3518**	**0.0068**
				52	428576.4	393704.4	9.77E+08	9.86E+08	-29.6537	-28.6339	2606.896	2362.627	0.039043	0.034717	2.75E-05	2338.184	3.19E-05	0.003631	2.398141				0.0069			0.0080					
				53	428866.1	393957.1	9.77E+08	9.86E+08	-29.6488	-28.6331	2605.236	2361.236	0.039046	0.034737	2.77E-05	2339.569	3.16E-05	0.003564	2.372552				0.0070			0.0080					
BPH3	CPME	0.98 GHz	PPR	C1	424101.4	392084.2	9.78E+08	9.86E+08	-29.6249	-28.6626	2617.479	2388.339	0.038914	0.034833	2.4E-05	2359.211	2.92E-05	0.003616	2.425514	**2.4109**	**1.4109**	**0.0085**	0.0058	**0.0060**	**0.0001**	0.0071	0.0070	0.0000	**1.4048**	**2.4185**	**0.0061**
				C2	425020.2	392307	9.78E+08	9.86E+08	-29.6124	-28.6632	2615.991	2383.386	0.038912	0.034883	2.49E-05	2362.712	2.85E-05	0.003579	2.411161				0.0060			0.0069					
				C3	425896.3	391755.4	9.78E+08	9.86E+08	-29.6227	-28.6589	2619.731	2378.474	0.038931	0.034842	2.57E-05	2359.828	2.91E-05	0.003541	2.395904				0.0062			0.0071					
BPH3	TBME	0.98 GHz	PPR	D1	434303.7	392193.3	9.78E+08	9.86E+08	-29.8226	-28.6591	2616.807	2330.856	0.03893	0.034049	3.43E-05	2304.238	3.93E-05	0.003395	2.338572	**2.3920**	**1.3920**	**0.0299**	0.0083	**0.0088**	**0.0002**	0.0095	0.0098	0.0001	**1.3832**	**2.3966**	**0.0088**
				D2	436638.1	392490.6	9.78E+08	9.86E+08	-29.8589	-28.663	2614.781	2317.444	0.038912	0.033907	3.68E-05	2294.28	4.12E-05	0.003658	2.441987				0.0089			0.0100					
				D3	436952.7	392491.7	9.78E+08	9.86E+08	-29.8546	-28.6612	2614.8	2316.09	0.038921	0.033924	3.71E-05	2295.477	4.09E-05	0.003539	2.395448				0.0090			0.0099					
BPH3	Eucalyptol	0.98 GHz	PPR	61	434093.7	393533.6	9.77E+08	9.86E+08	-29.7497	-28.6317	2608.056	2331.812	0.039052	0.034336	3.31E-05	2311.592	3.68E-05	0.003573	2.375829	**2.3802**	**1.3802**	**0.0178**	0.0083	**0.0083**	**0.0001**	0.0093	0.0093	0.0001	**1.3719**	**2.3831**	**0.0083**
				62	433990.8	393295.7	9.77E+08	9.86E+08	-29.7615	-28.6299	2609.658	2332.026	0.039061	0.034289	3.3E-05	2308.324	3.74E-05	0.00367	2.413053				0.0083			0.0094					
				63	433300.8	393406.1	9.78E+08	9.86E+08	-29.7361	-28.6291	2608.937	2336.356	0.039064	0.03439	3.22E-05	2315.334	3.61E-05	0.00351	2.351705				0.0081			0.0091					
246triF	Et2O	0.98 GHz	PPR	51	465701.1	392874.2	9.77E+08	9.86E+08	-30.3887	-28.6279	2612.356	2167.866	0.03907	0.031901	6.77E-05	2147.907	7.2E-05	0.003726	2.408959	**2.3959**	**1.3959**	**0.0111**	0.0154	**0.0155**	**0.0001**	0.0164	0.0164	0.0001	**1.3804**	**2.4011**	**0.0156**
				52	464973.1	392023	9.77E+08	9.86E+08	-30.3701	-28.6196	2618.133	2171.438	0.039107	0.031969	6.7E-05	2152.666	7.1E-05	0.003714	2.404726				0.0153			0.0162					
				53	466926.1	392623.8	9.77E+08	9.86E+08	-30.3943	-28.6288	2614.018	2162.334	0.039066	0.03188	6.89E-05	2146.478	7.23E-05	0.003633	2.373875				0.0157			0.0165					
246triF	CPME	0.98 GHz	PPR	61	514389.9	393137.6	9.77E+08	9.86E+08	-31.2335	-28.6338	2610.543	1956.808	0.039043	0.028944	0.000117	1942.903	0.000121	0.003668	2.387171	**2.3916**	**1.3916**	**0.0060**	0.0268	**0.0268**	**0.0005**	0.0276	0.0278	0.0006	**1.3648**	**2.3962**	**0.0269**
				62	510854.6	393175.9	9.77E+08	9.86E+08	-31.1843	-28.6301	2610.337	1970.699	0.03906	0.029108	0.000114	1954.27	0.000118	0.00366	2.384055				0.0260			0.0269					
				63	518552.3	391967	9.77E+08	9.86E+08	-31.327	-28.6229	2618.48	1940.397	0.039092	0.028634	0.000122	1921.494	0.000127	0.003711	2.403558				0.0278			0.0289					
246triF	TBME	0.98 GHz	PPR	71	539617.2	393807	9.77E+08	9.86E+08	-31.6671	-28.6371	2606.076	1862.486	0.039028	0.027534	0.000143	1845.611	0.000148	0.003741	2.414794	**2.4201**	**1.4201**	**0.0109**	0.0327	**0.0313**	**0.0009**	0.0338	0.0324	0.0009	**1.3888**	**2.4293**	**0.0318**
				72	526466.4	393210.3	9.77E+08	9.86E+08	-31.4573	-28.6364	2610.043	1910.384	0.039032	0.028208	0.00013	1892.043	0.000135	0.003714	2.404435				0.0296			0.0308					
				73	534872	393092.9	9.77E+08	9.86E+08	-31.5803	-28.6332	2610.863	1879.413	0.039046	0.027811	0.000139	1864.675	0.000143	0.00381	2.440924				0.0316			0.0325					
246triF	Eucalyptol	0.98 GHz	PPR	81	523940.9	392500.5	9.77E+08	9.86E+08	-31.404	-28.6287	2614.861	1920.022	0.039066	0.028381	0.000127	1904.039	0.000132	0.003669	2.387616	**2.4049**	**1.4049**	**0.0087**	0.0290	**0.0287**	**0.0002**	0.0300	0.0297	0.0002	**1.3762**	**2.4116**	**0.0290**
				82	522972.7	392660	9.77E+08	9.86E+08	-31.384	-28.6305	2613.78	1923.563	0.039058	0.028447	0.000126	1908.55	0.00013	0.003743	2.415679				0.0288			0.0297					
				83	520743.3	392643.8	9.77E+08	9.86E+08	-31.3572	-28.6365	2613.82	1931.994	0.039031	0.028535	0.000124	1914.63	0.000129	0.003732	2.411502				0.0283			0.0293					
pentaF	Et2O	0.98 GHz	PPR	21	1313502	383244.6	9.77E+08	9.86E+08	-39.379	-28.6076	2678.302	752.2512	0.039161	0.011331	0.000934	745.1081	0.000946	0.004166	2.604366	**2.5840**	**1.5840**	**0.0126**	0.2356	**0.2325**	**0.0018**	0.2388	0.2359	0.0018	**1.3515**	**2.6376**	**0.2549**
				22	1288900	392868.7	9.77E+08	9.86E+08	-39.2225	-28.6254	2612.494	766.8562	0.039081	0.011537	0.000908	758.8144	0.000922	0.004054	2.560996				0.2292			0.2327					
				23	1302131	393644	9.77E+08	9.86E+08	-39.3073	-28.6298	2607.302	758.9276	0.039061	0.011425	0.000922	751.3553	0.000935	0.00412	2.586615				0.2327			0.2360					
pentaF	CPME	0.98 GHz	PPR	A1	1123405	391526.7	9.77E+08	9.86E+08	-38.0747	-28.651	2621.32	881.5181	0.038966	0.013167	0.00074	872.2343	0.000752	0.003976	2.56737	**2.5542**	**1.5542**	**0.0186**	0.1796	**0.1787**	**0.0011**	0.1825	0.1815	0.0014	**1.3755**	**2.5975**	**0.1932**
				A2	1125199	392642.8	9.77E+08	9.86E+08	-38.102	-28.6603	2613.76	880.0524	0.038925	0.013126	0.000742	869.4652	0.000755	0.004002	2.577638				0.1800			0.1834					
				A3	1111051	392280.7	9.77E+08	9.86E+08	-37.9569	-28.6607	2616.173	891.596	0.038923	0.013347	0.000727	884.309	0.000736	0.003849	2.517458				0.1764			0.1787					
pentaF	TBME	0.98 GHz	PPR	B1	1337527	392429.2	9.77E+08	9.86E+08	-39.5963	-28.6599	2615.197	738.767	0.038926	0.011052	0.000959	730.5075	0.000974	0.004038	2.591935	**2.5970**	**1.5970**	**0.0043**	0.2328	**0.2339**	**0.0006**	0.2365	0.2375	0.0006	**1.3631**	**2.6554**	**0.2580**
				B2	1346316	392596.6	9.77E+08	9.86E+08	-39.6535	-28.6624	2614.057	733.8652	0.038915	0.010979	0.000968	725.6608	0.000983	0.004072	2.60545				0.2350			0.2387					
				B3	1341913	392294.6	9.77E+08	9.86E+08	-39.617	-28.6616	2616.084	736.3302	0.038919	0.011025	0.000963	728.7506	0.000978	0.004042	2.593607				0.2339			0.2373					
pentaF	Eucalyptol	0.98 GHz	PPR	EU1	951623.1	398169.2	9.77E+08	9.86E+08	-36.5781	-28.7308	2576.317	1043.022	0.038609	0.015643	0.000558	1032.263	0.000568	0.004008	2.545917	**2.5301**	**1.5301**	**0.0138**	0.1445	**0.1430**	**0.0022**	0.1471	0.1455	0.0023	**1.3871**	**2.5659**	**0.1528**
				EU2	929613.7	397503.6	9.77E+08	9.86E+08	-36.3647	-28.7231	2580.724	1068.26	0.038644	0.016032	0.000535	1058.363	0.000544	0.003895	2.502502				0.1387			0.1409					
				EU3	956116.4	398146.7	9.77E+08	9.86E+08	-36.6229	-28.7253	2576.531	1038.047	0.038634	0.015563	0.000563	1026.868	0.000573	0.003997	2.541768				0.1457			0.1484					

NMR of hydrogenation reaction

To a 0.1 M solution of $PMes_3$ PentaF(LA3LB3) in dry toluene in a schlenk flask, the flask was bubbled with 1 bar hydrogen gas. A fraction of the mixture of partially hydrogenated (LA3LB3) were taken out and a crude NMR were monitored. We make the following key correlations between our crude sample and a purified sample reported in the literature (Ref 2b, main text, in a different solvent):

Reported pure [$(C_6H_2Me\text{-}2,4,6)_3PH$][$HB(C_6F_5$] in ($C_6D_5Br$) ^{1}HNMR ($C_6D_5Br$, 300 MHz, 300K) δ 7.91 (d,^{1}H, $^1J_{H\text{-}P}$ = 480 Hz, P*H.*).- Found δ 7.99 (d, ^{1}H, $^1J_{H\text{-}P}$ = 479.7 Hz, P*H*). Reported δ 4.10(q,1H, $^1J_{H\text{-}B}$=112 Hz, B*H*) found, δ 4.28(br). Aromatic region is overlapped with toluene solvent residue.

Reported $^{31}P\{^1H\}$ (C_6D_5Br, 121Mhz), δ -27.53 (s). Found ^{31}P NMR with ^{1}H coupling (162 MHz, Tol) δ -28.08(d, *J*= 478.7 Hz), -36.57.(s, remaining starting material $PMes_3$).Reported $^{11}B\{^1H\}$ NMR (C_6D_6Br, 96 MHz) δ-25.47 (s), Found^{11}B NMR with ^{1}H coupling δ (128MHz dtol)-24.94 (br), ^{19}F and ^{13}C NMR was not recorded.

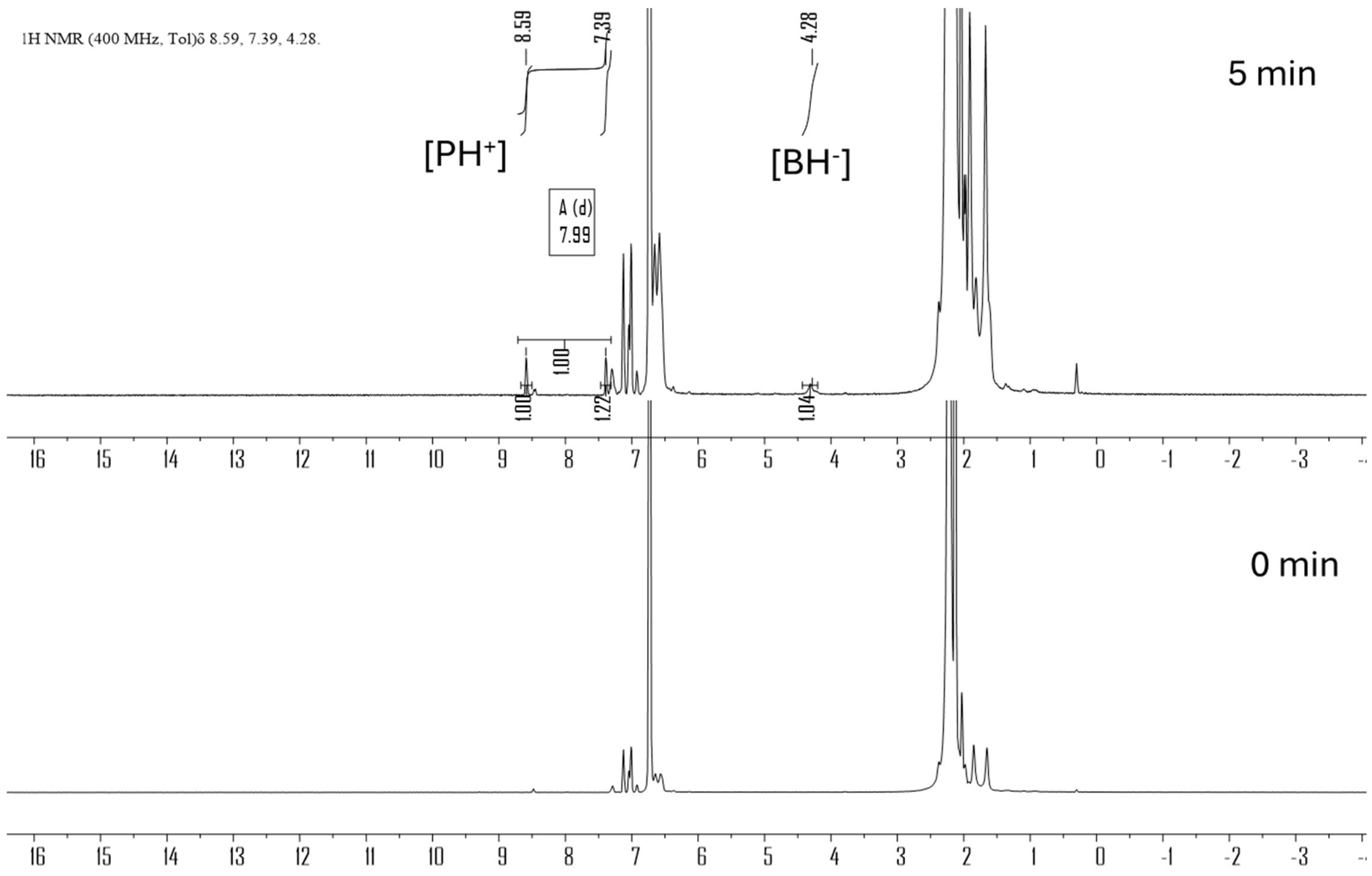
1H NMR (400 MHz, Tol)δ 8.59, 7.39, 4.28.
8.59
7.39
4.28
[PH+]
[BH-]
5 min
A (d)
7.99
1.00
1.00
1.22
1.04
0 min

31P NMR (162 MHz, Tol)δ -36.57.

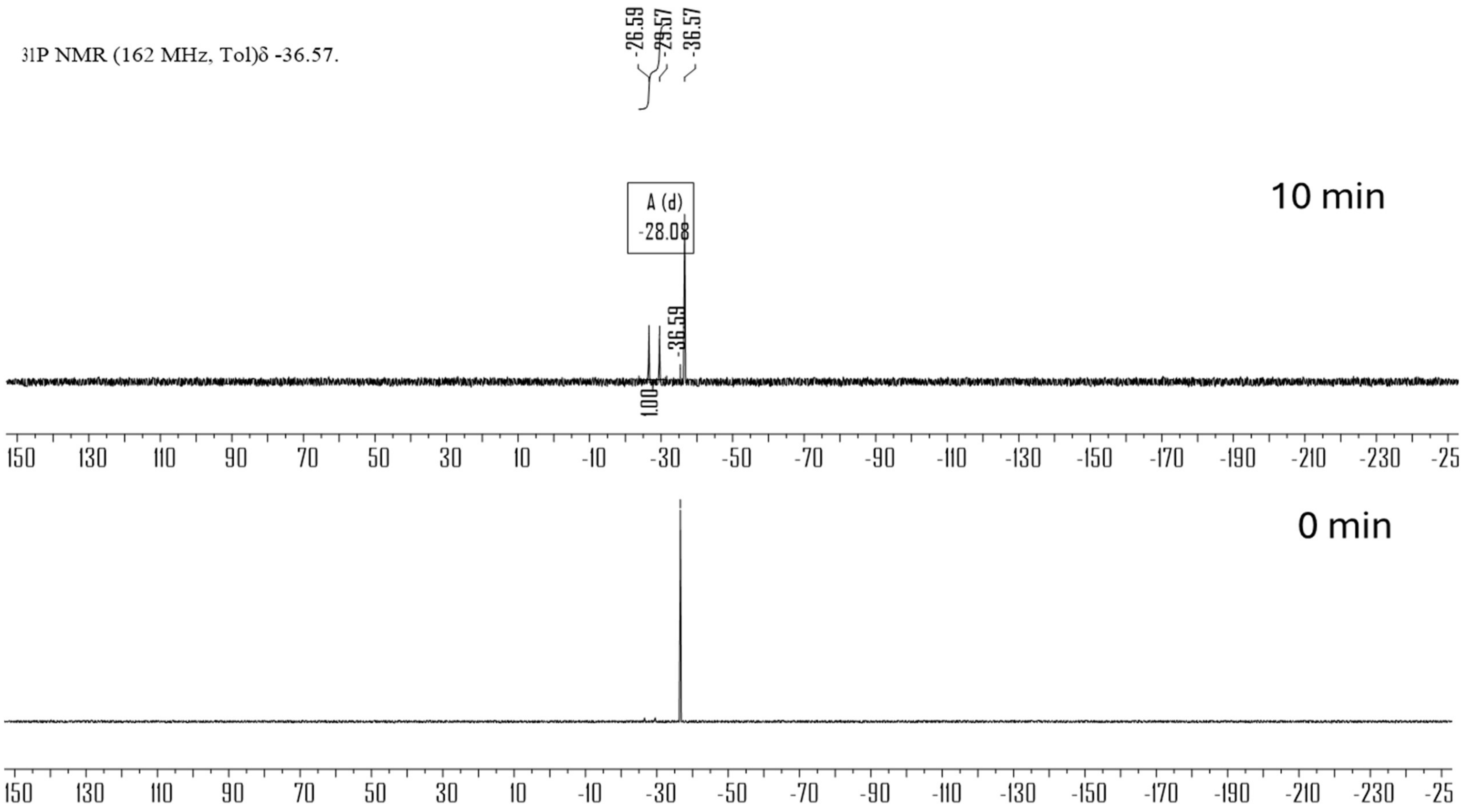

^{11}B NMR (128 MHz, Tol) δ 60.73.

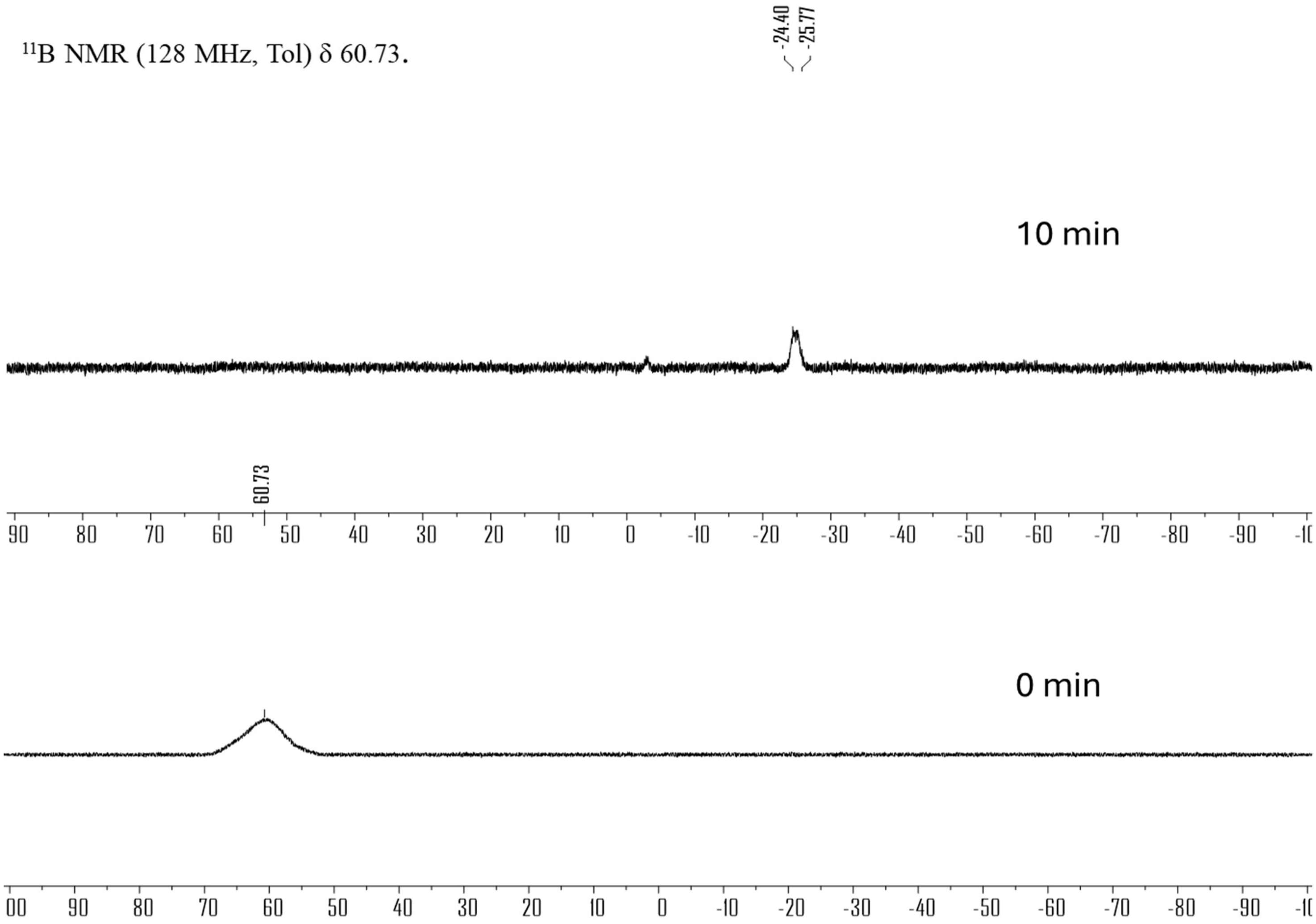

S3.7 Control experiment - leaving the FLP sample open to air and the resultant MDS response

Three samples were prepared: 0.1M tris(pentafluorophenyl)borane (**LA3**), 0.1M trimesitylphosphine (**LA2**), and a 0.1M FLP mixture of tris(pentafluorophenyl)borane and trimesitylphosphine in an FEP tube. The samples were measured with MDS (t_0). Following this initial measurement, the cap of the tube was removed and the sample was left open to air atmosphere. The MDS measurements of the sample were re-taken after one and two days of the cap being off.

As can be seen in Figure 3.7 the response of $P(Mes)_3$ remains relatively unchanged over the 2 days left open to air atmosphere whereas that of tris(pentafluorophenyl)borane and the FLP mixture show a significant increase in their MDS response (giving ε_2 values beyond anything else seen in the context of this project).

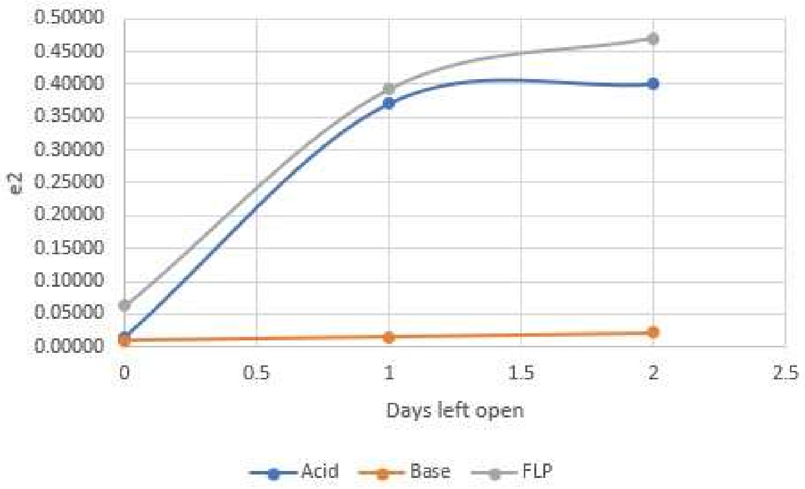

Figure 3.7 Control experiment of FLP air sensitivity

This data suggests that interaction between water in the atmosphere and the borane will lead to drastically increased ε_2 values measured by MDS; observable in both the LA and FLP samples. This observation serves to help highlight any 'wet' borane samples.

S4. Hydrogen extraction of terpinene by Lewis pairs (Table 1c)

Under nitrogen atmosphere in glove box, the Lewis acid 0.05 mmol, the Lewis base, and tyripiene were dissolved in dry deuterated toluene (0.6 ml) in a sealed microwave tube equipped with stirring bar. The reaction tube was heated to 80 °C for 2 hours, after which the reaction was quenched by open to air. The resulting mixture were characterised by ^{1}H NMR(400 MHz).

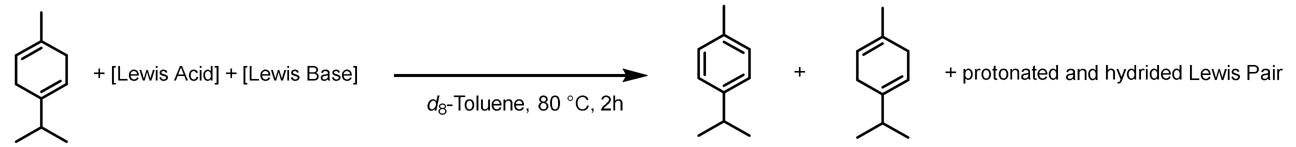

NMR Yield	BPh3	246triF	pentaF
PPh_3	2 %	3 %	3 %
$P(o\text{-}tol)_3$	4 %	3 %	87 %
$P(xyl)_3$	4 %	3 %	72 %
$P(mes)_3$	9 %	3 %	75 %
Pyridine	2 %	3 %	2 %
Picoline	4 %	2 %	7 %
Lutidine	5 %	3 %	60 %
Collidine	6 %	3 %	62 %
Et2O	6 %	4 %	77 %
CPME	6 %	5 %	84 %
TBME	4 %	4 %	38 %
Eucalyptol	7 %	9 %	78 %

- Only pentaF gives a 30 % NMR yield without addition of Lewis base.

S5 NMR Spectra

Figure S5.1: ^{1}H NMR (400 MHz, $CDCl_3$, 298 K) spectrum of tris(2,4,6-trifluorophenyl)borane **(LA3)(pentaF))**

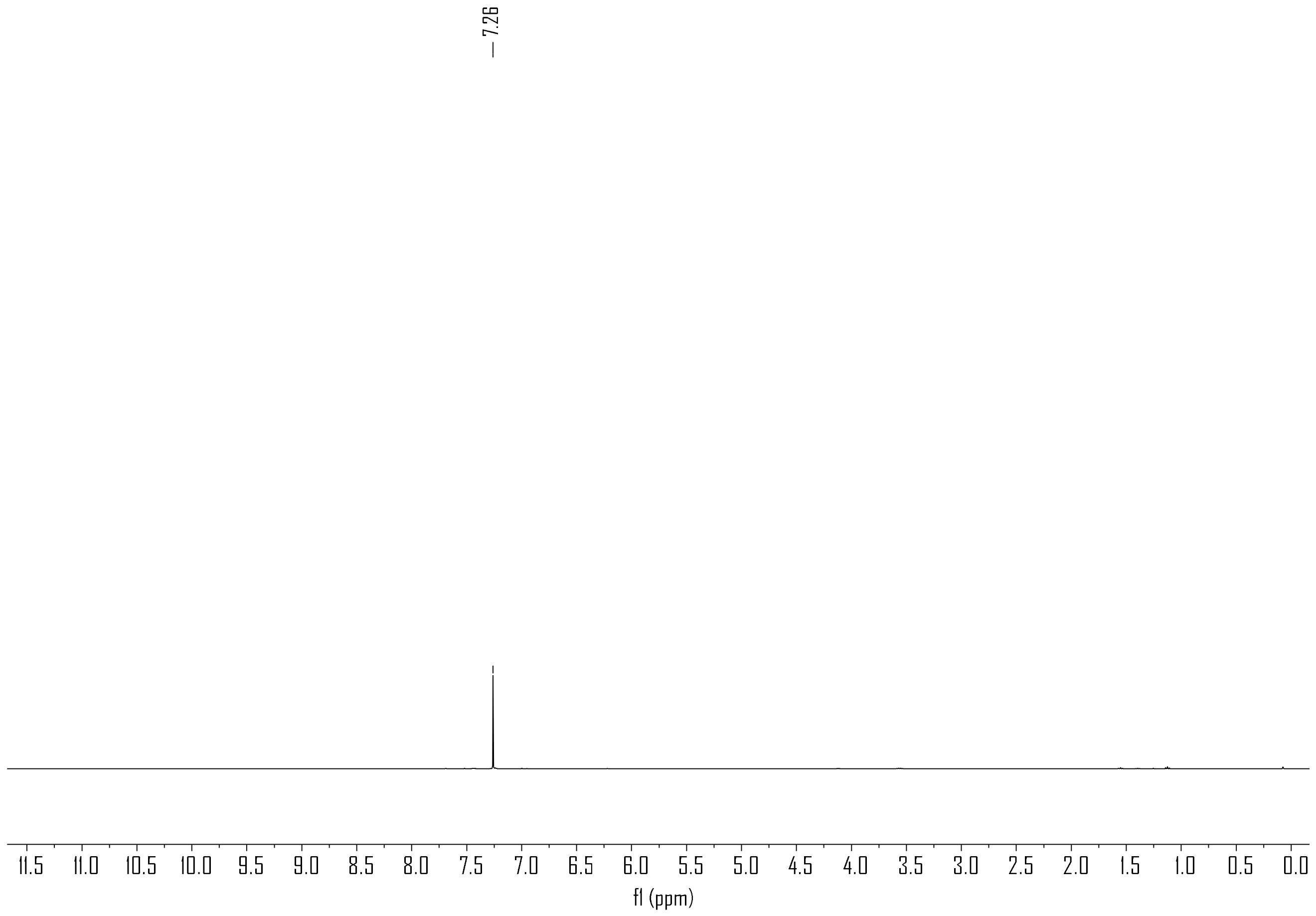

Figure S5.2: ^{11}**B NMR** (128 MHz, $CDCl_3$, 298 K) spectrum of tris(pentafluorophenyl)borane **(LA3)**

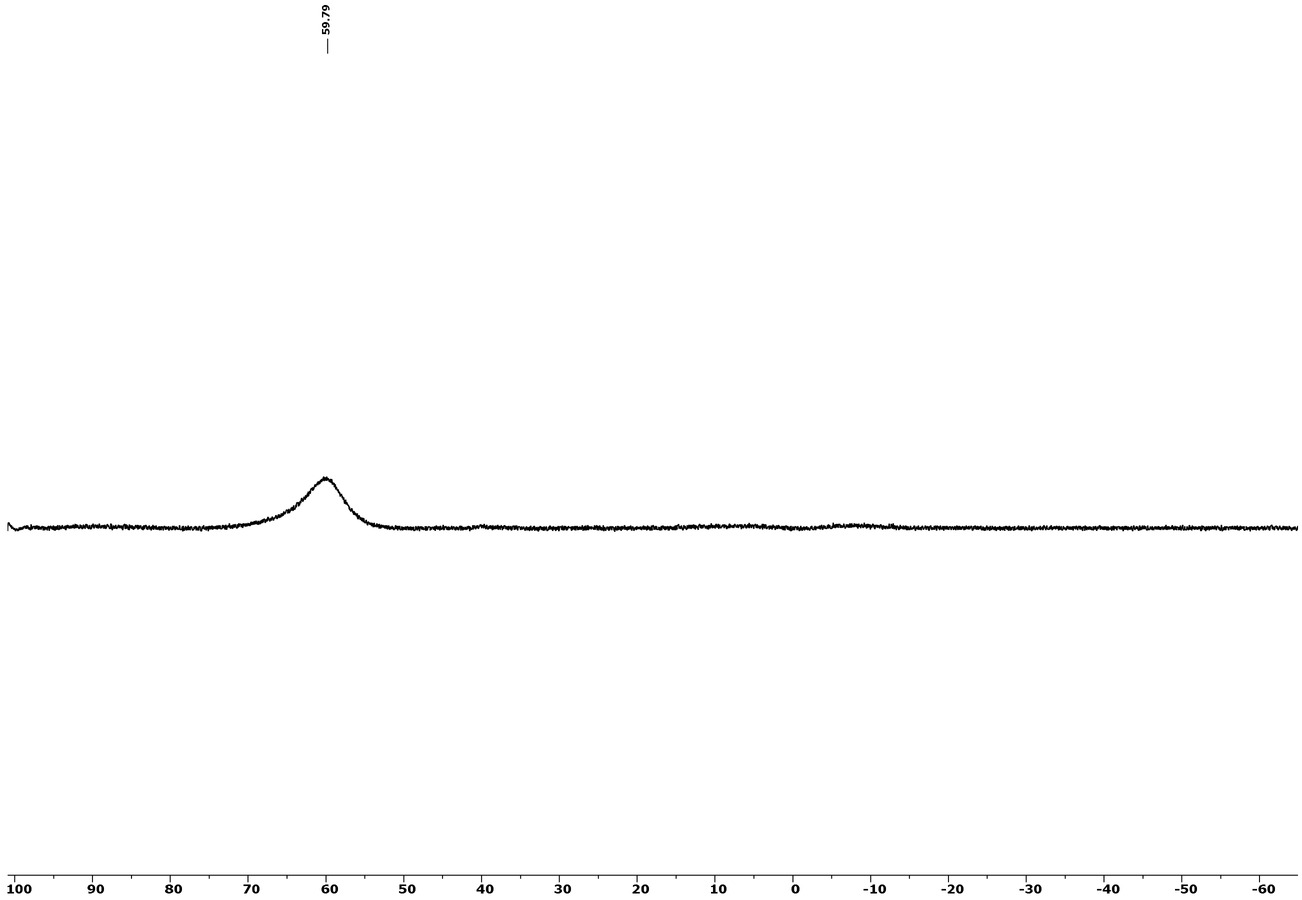

Figure S5.3: **^{19}F NMR** (376 MHz, $CDCl_3$, 298K) spectrum of tris(pentafluorophenyl)borane **(LA3)**

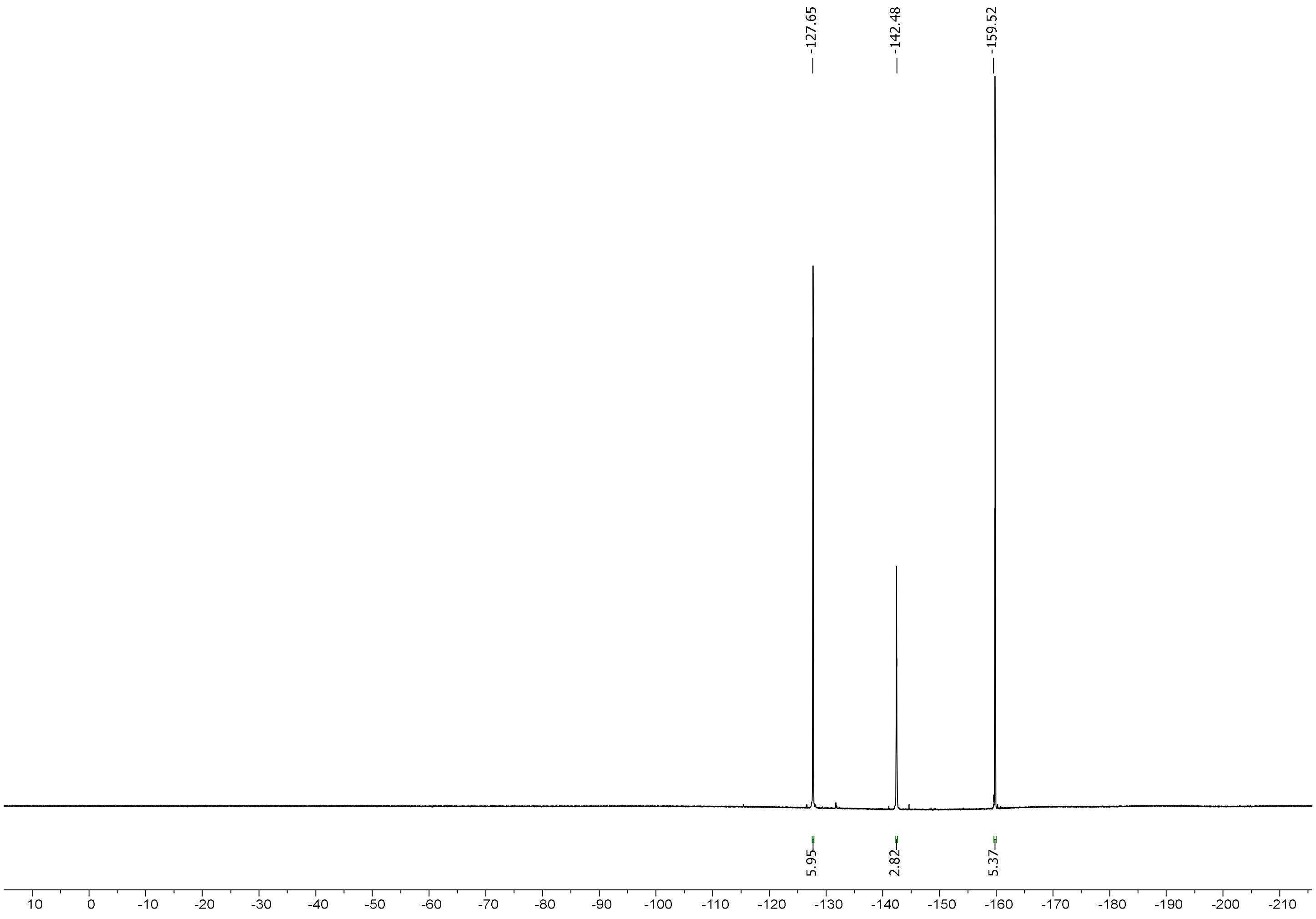

Figure S5.4: ^{1}**H NMR** (400 MHz, $CDCl_3$, 298 K) spectrum of tris(2,4,6-trifluorophenyl)borane **(LA2)(2,4,6-triF)**

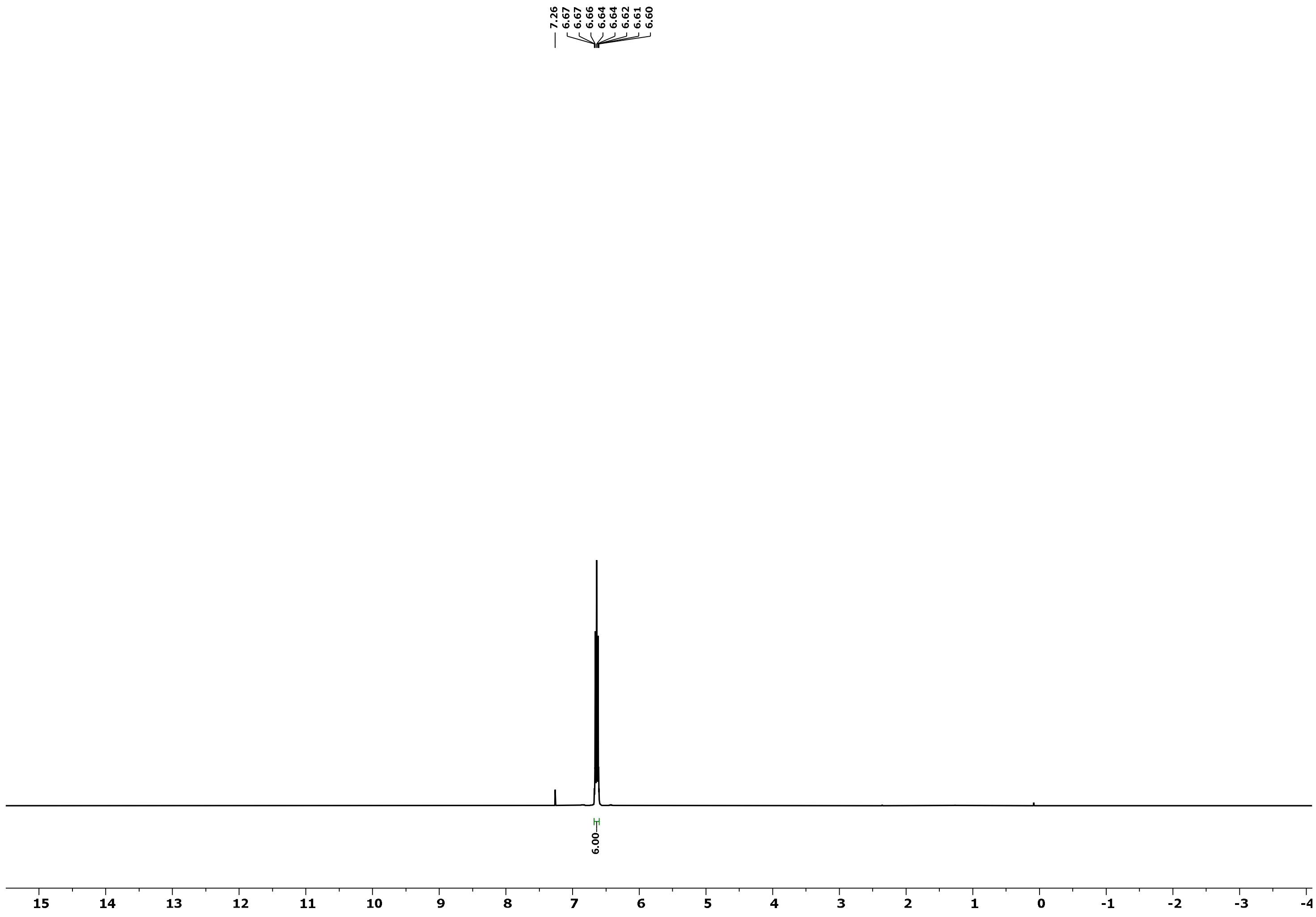

Figure S5.5:[11]**B NMR** (128 MHz, $CDCl_3$, 298 K) spectrum of tris(2,4,6-trifluorophenyl)borane **((LA2)(2,4,6-triF)**

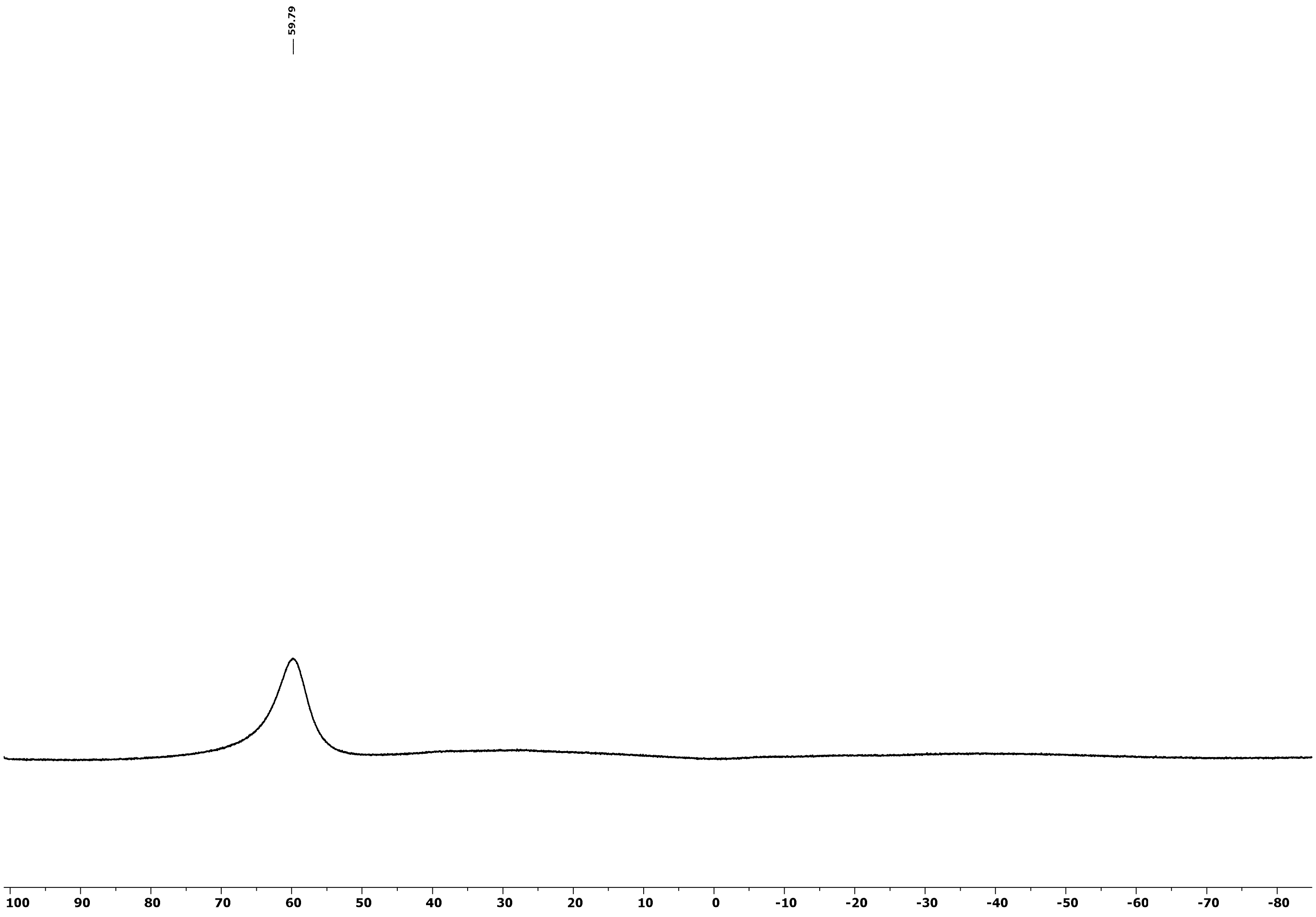

Figure S5.6: ^{19}**F NMR** (376 MHz, $CDCl_3$, 298K) spectrum of tris(2,4,6-trifluorophenyl)borane **(LA2)(2,4,6-triF)**

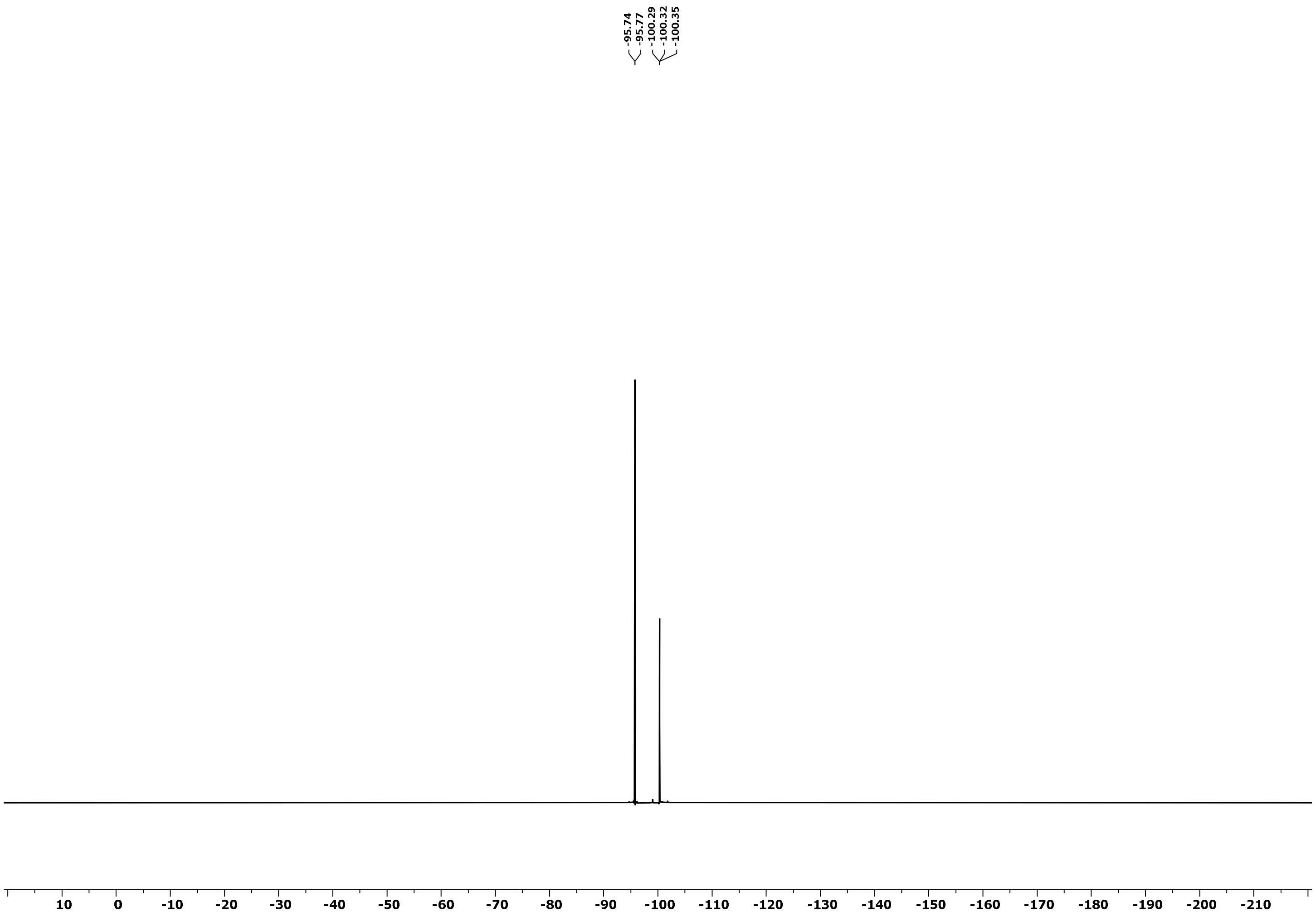

Figure S5.7: ^{1}**H NMR** (400 MHz, $CDCl_3$, 298 K) spectrum of tris(3,4,5-trifluorophenyl)borane **(3,4,5-triF)**

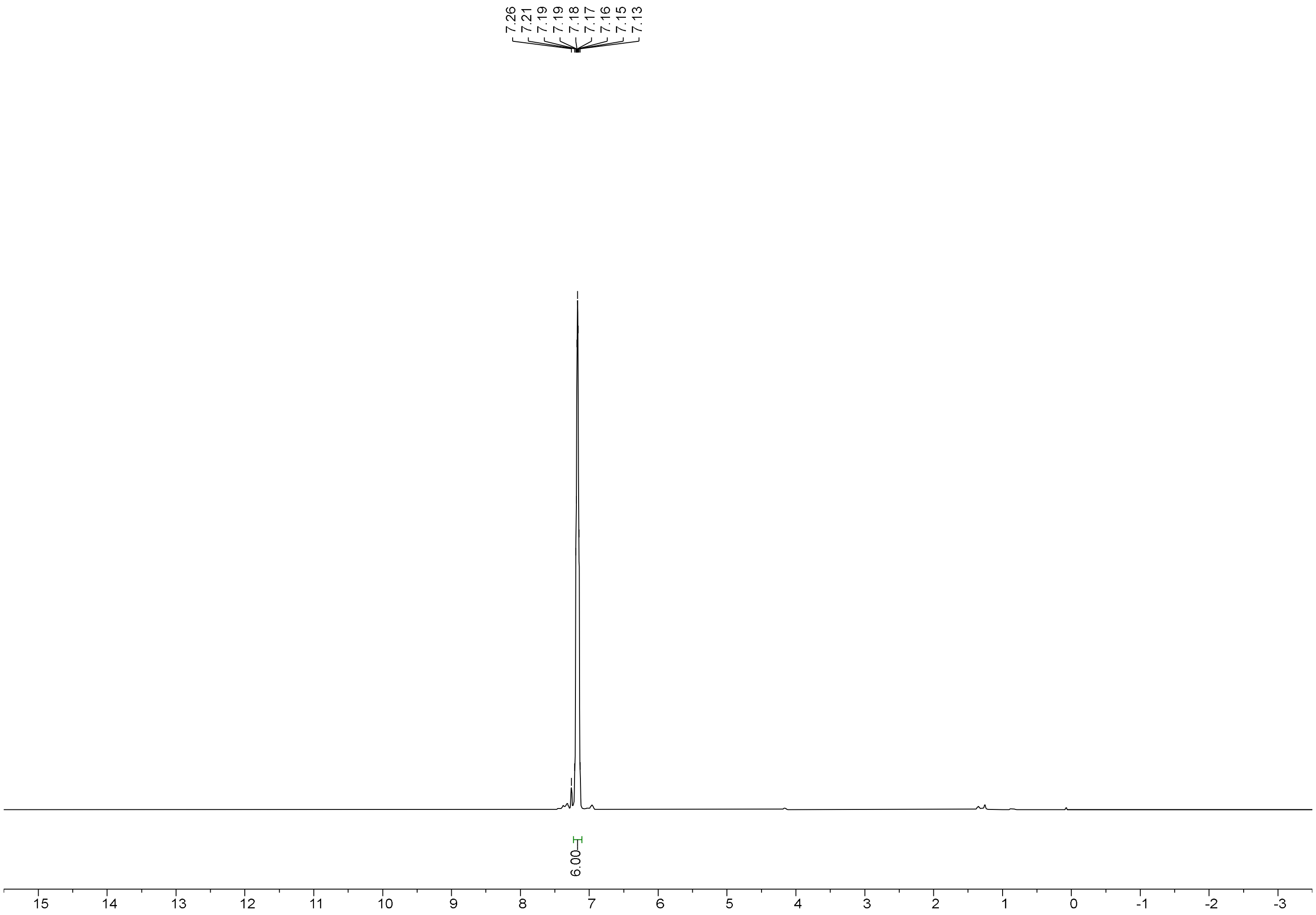

Figure S5.8: ^{11}**B NMR** (128 MHz, $CDCl_3$, 298 K) spectrum of tris(3,4,5-trifluorophenyl)borane **(3,4,5-triF)**

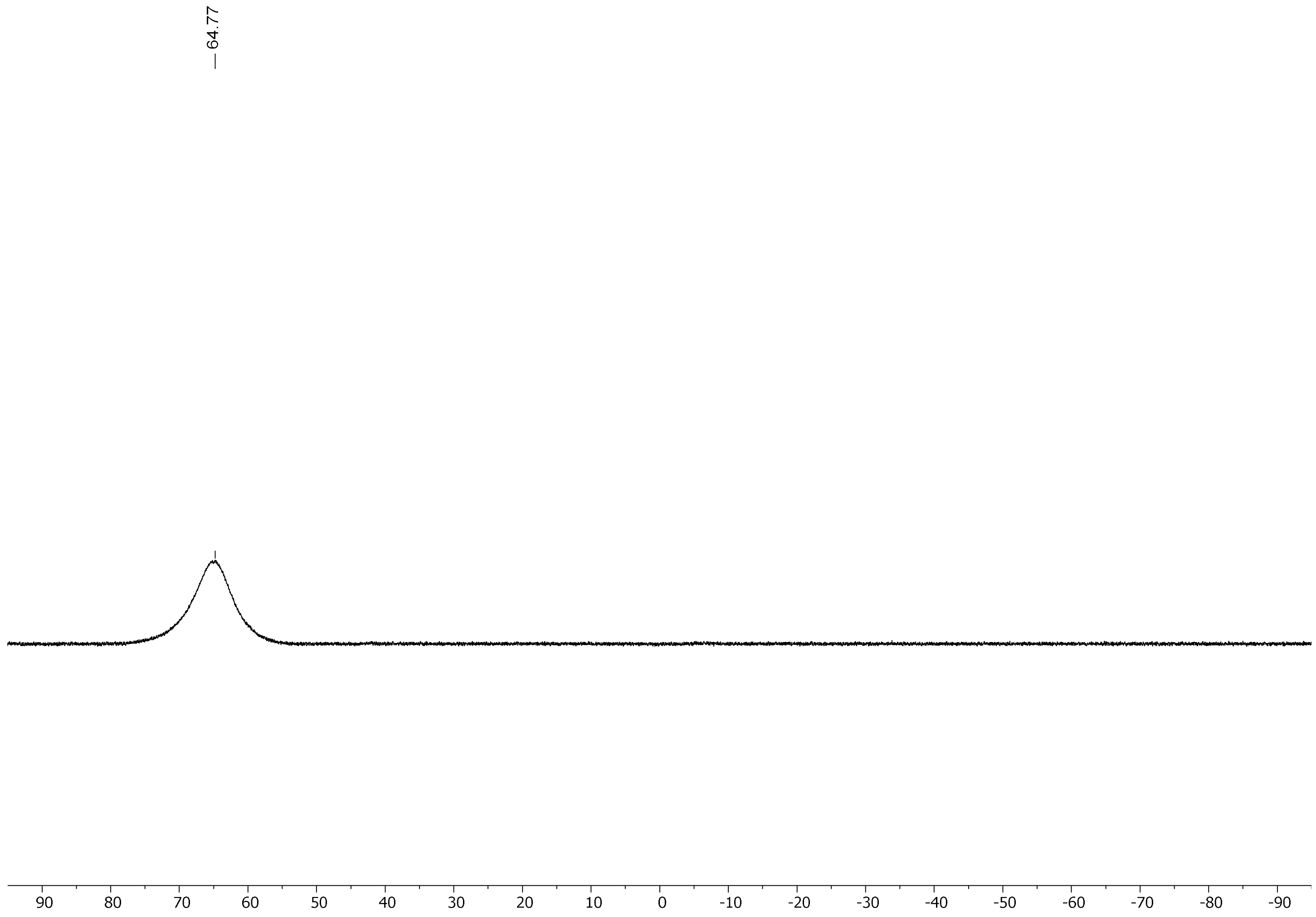

Figure S5.9: ^{19}**F NMR** (376 MHz, $CDCl_3$, 298 K) spectrum of tris(3,4,5-trifluorophenyl)borane **(3,4,5-triF)**

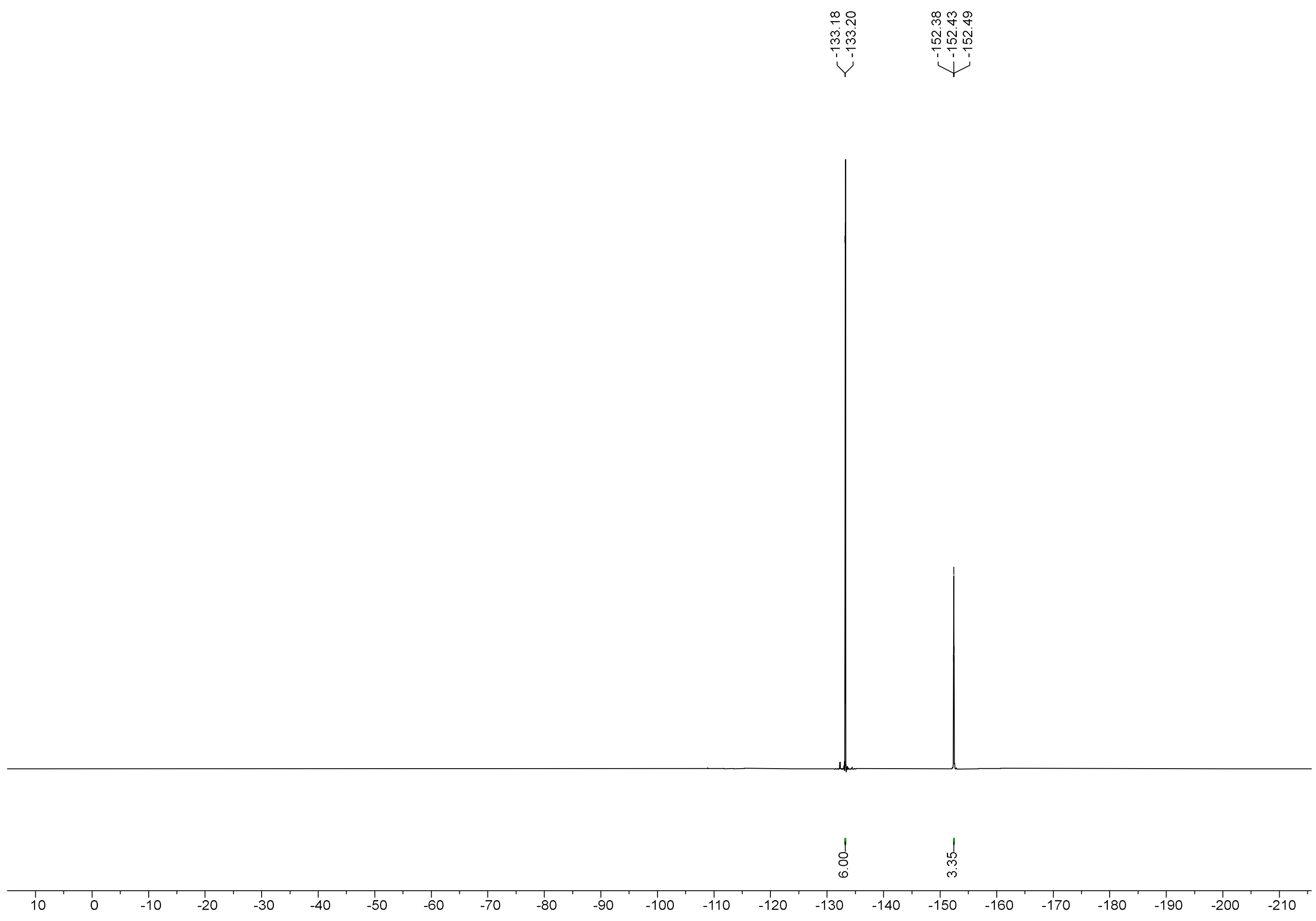

Figure S5.10: **^{1}H NMR** (400 MHz, $CDCl_3$, 298 K) spectrum of tris(4-fluorophenyl)borane **(4-F)**

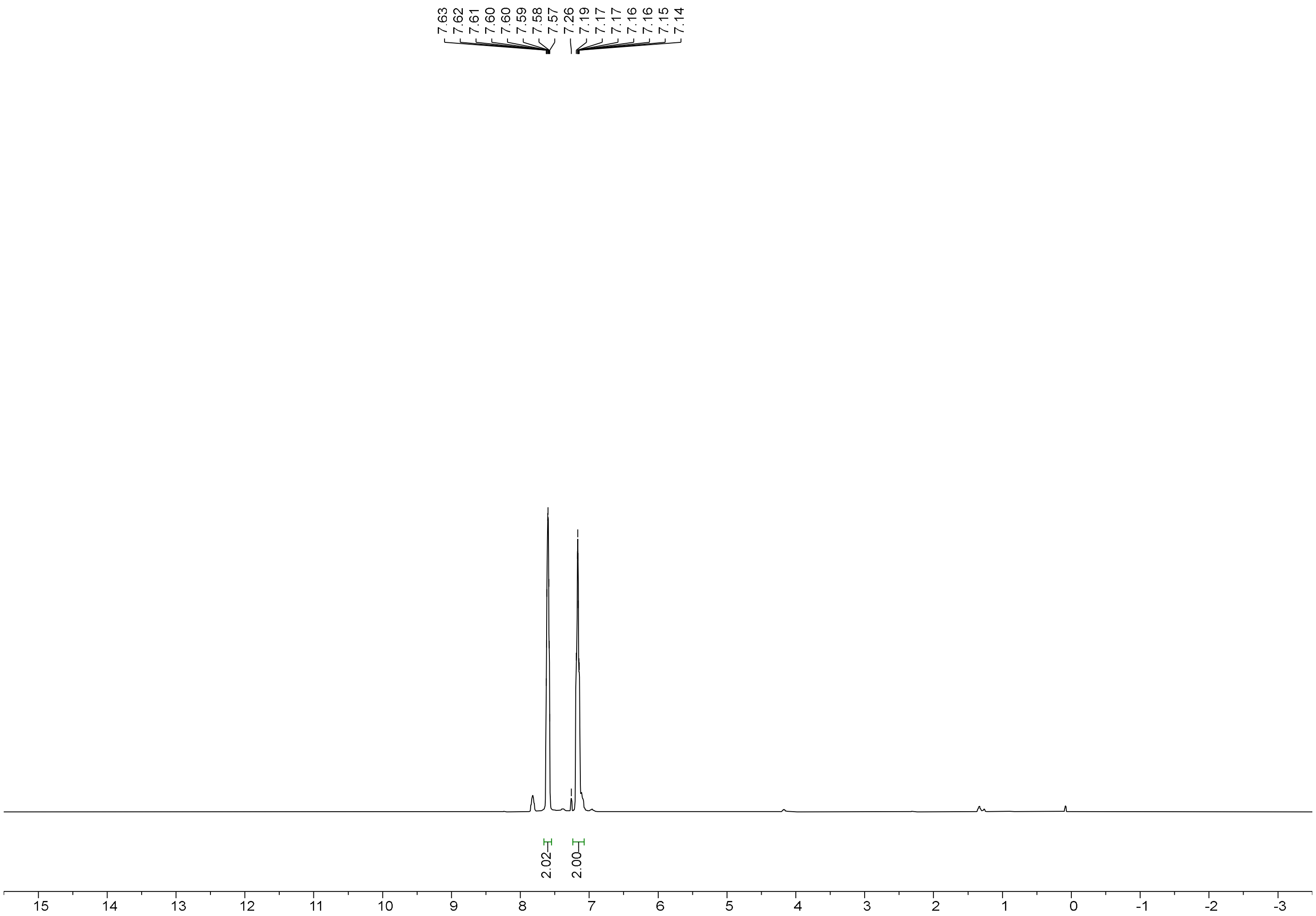

Figure S5.11: ^{11}**B NMR** (128 MHz, $CDCl_3$, 298 K) spectrum of tris(4-fluorophenyl)borane **(4-F)**

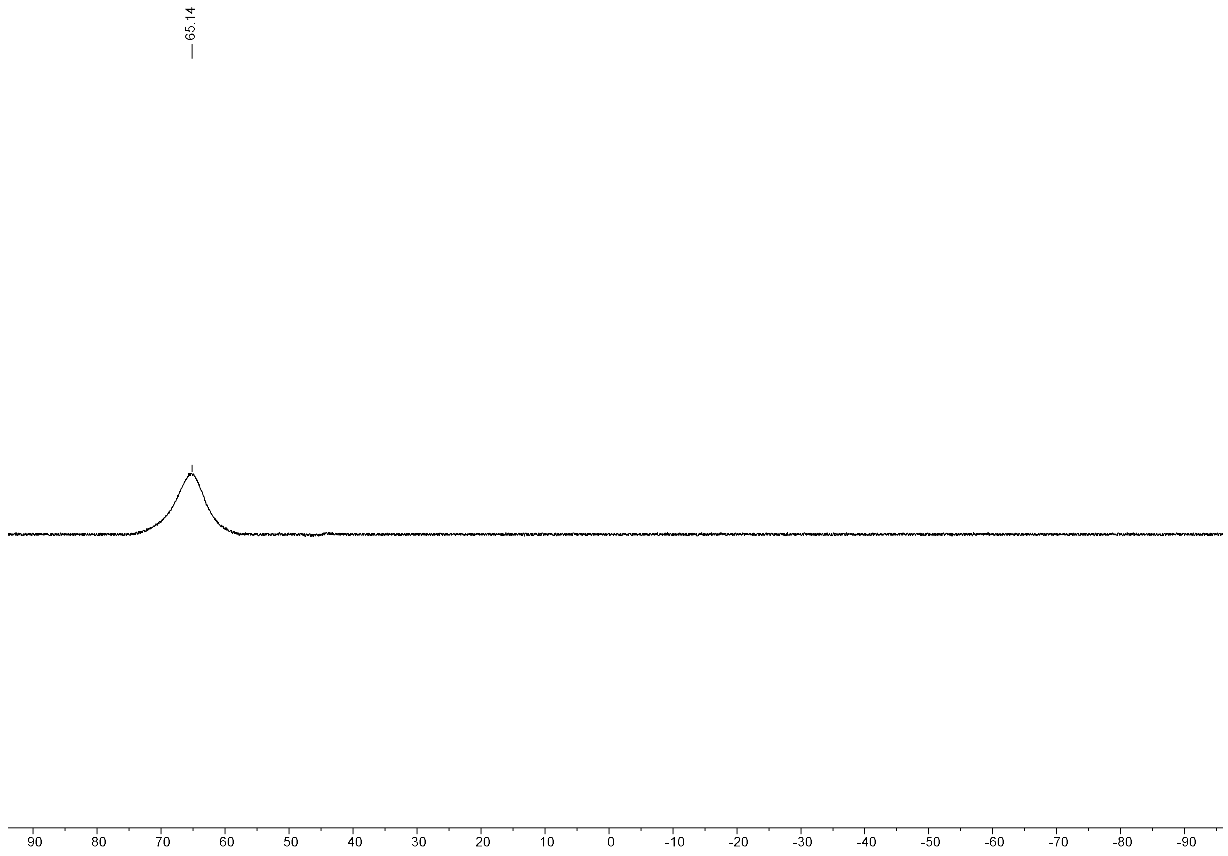

Figure S5.12:^{19}**F NMR** (376 MHz, $CDCl_3$, 298 K) spectrum of tris(4-fluorophenyl)borane **(4-F)**

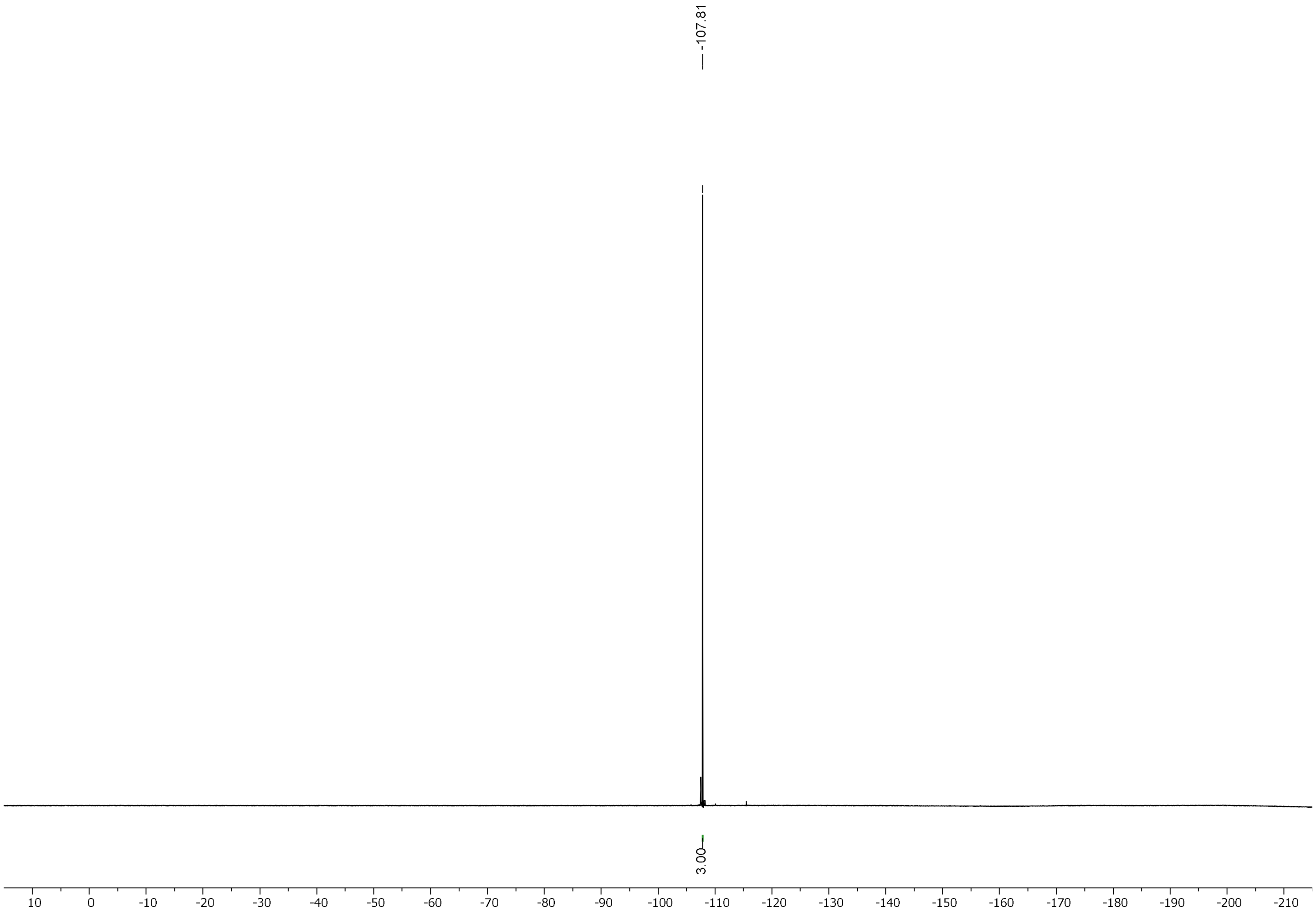

Figure S5.13:**^{1}H NMR** (400 MHz, $CDCl_3$, 298 K) spectrum of tri(o-toyl)phosphine (LB1)

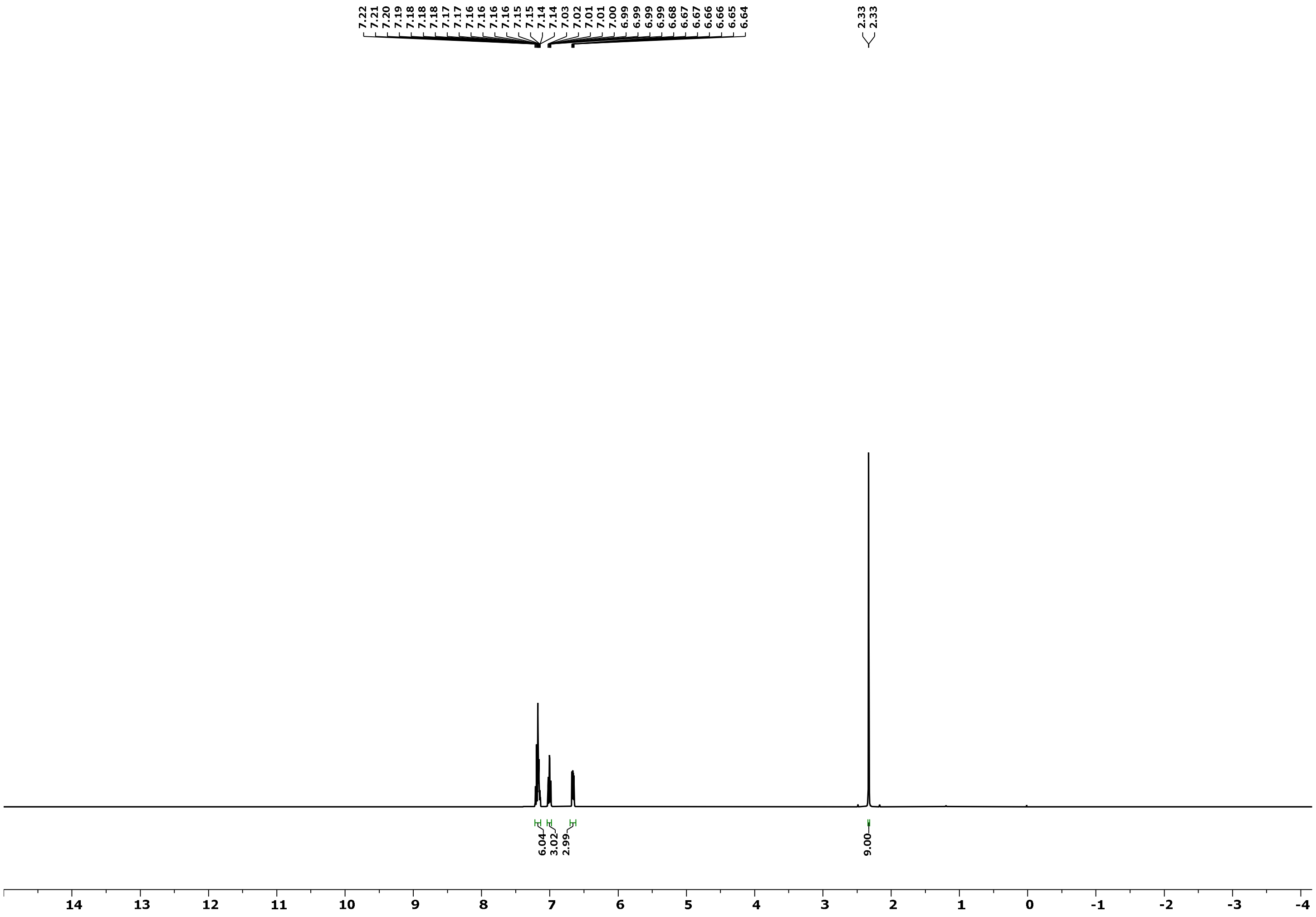

Figure S5.14:13C NMR (101 MHz, CDCl3, 298 K) spectrum of tri(o-toyl)phosphine (LB1)

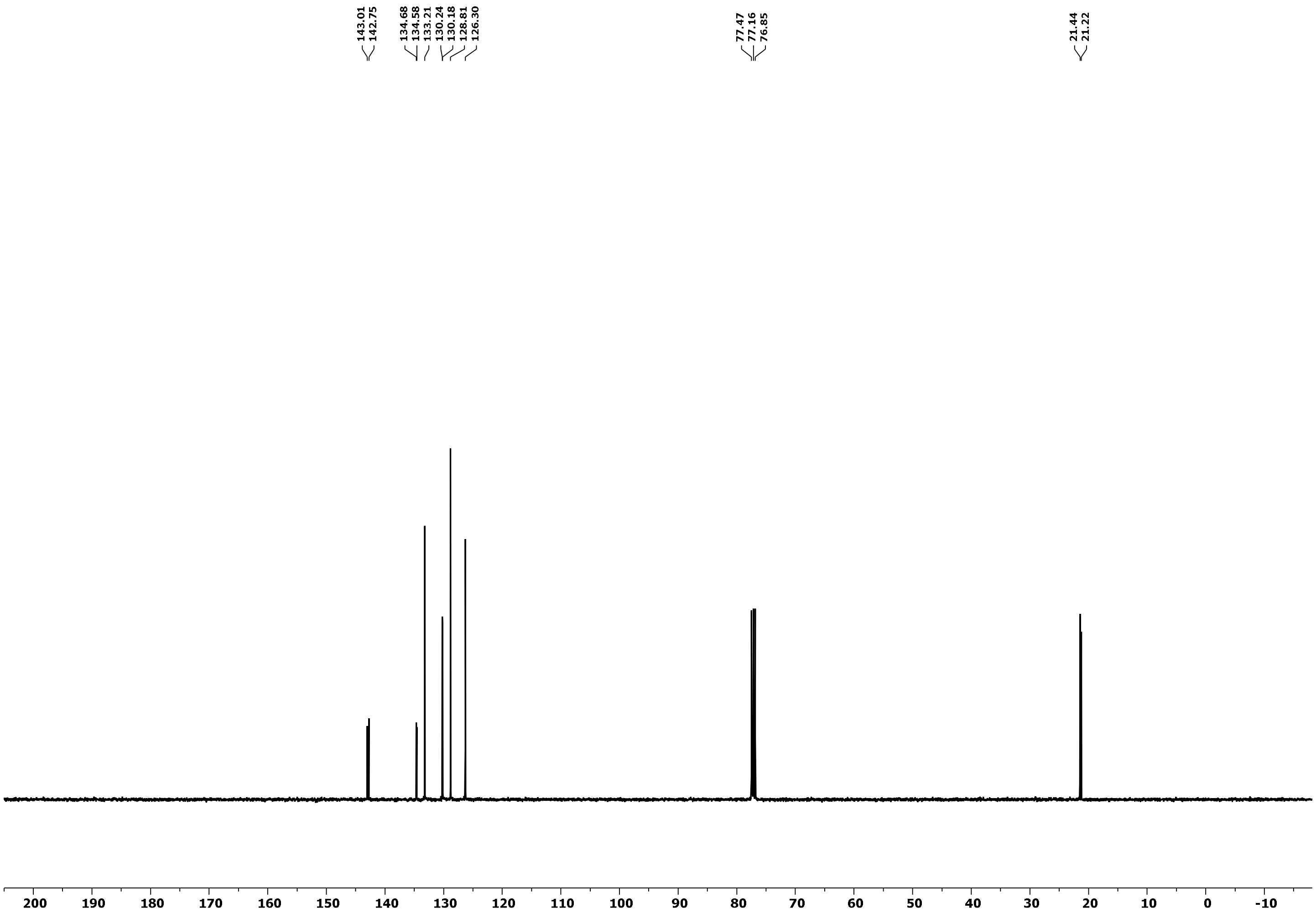

Figure S5.15: ^{31}**P NMR** (162MHZ, $CDCl_3$, 298 K) spectrum of tri(o-toyl)phosphine (LB1)

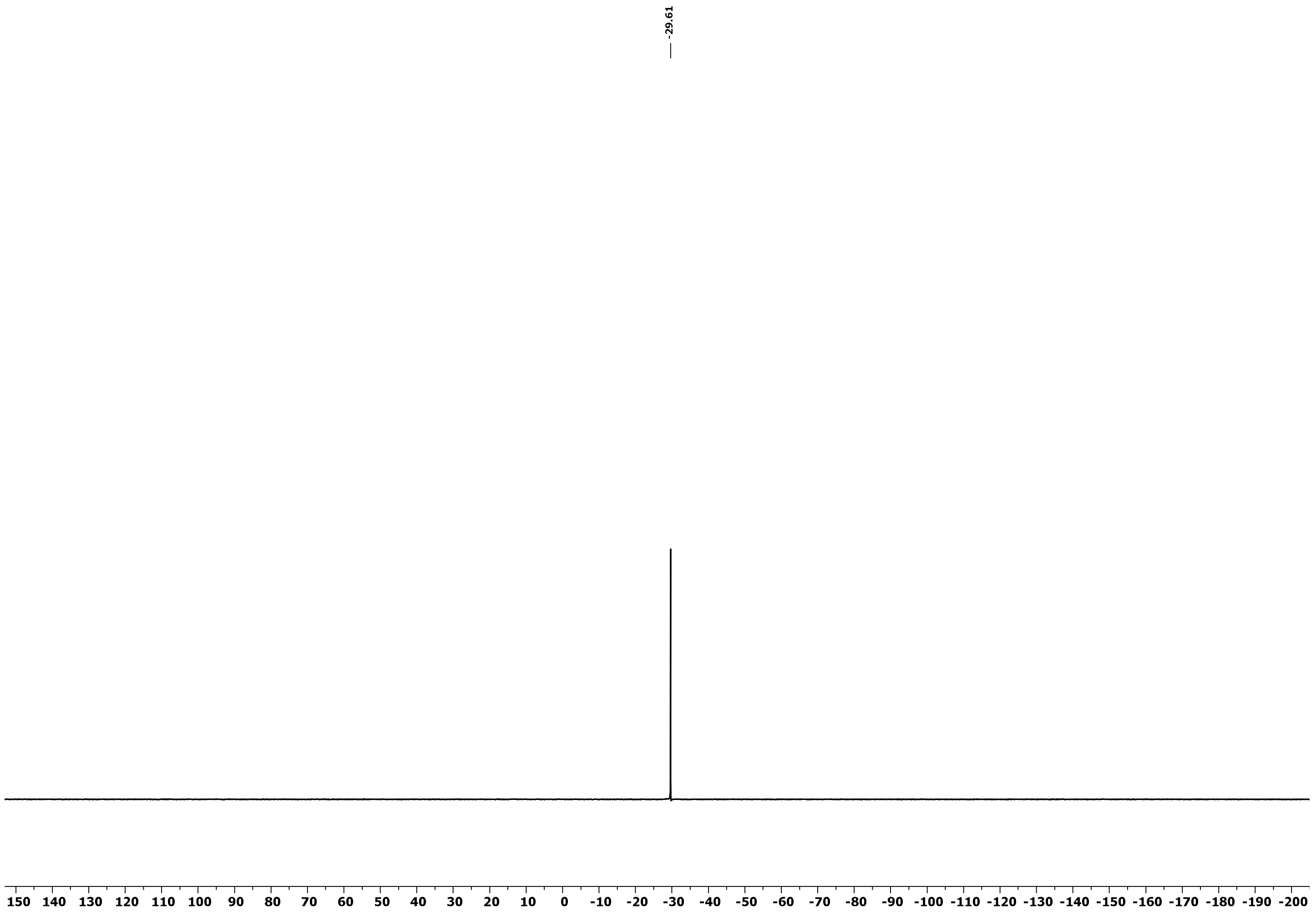

Figure S5.16: ^{1}**H NMR** (400 MHz, $CDCl_3$, 298 K) spectrum of tri(2,6-xyl)phosphine (LB2)

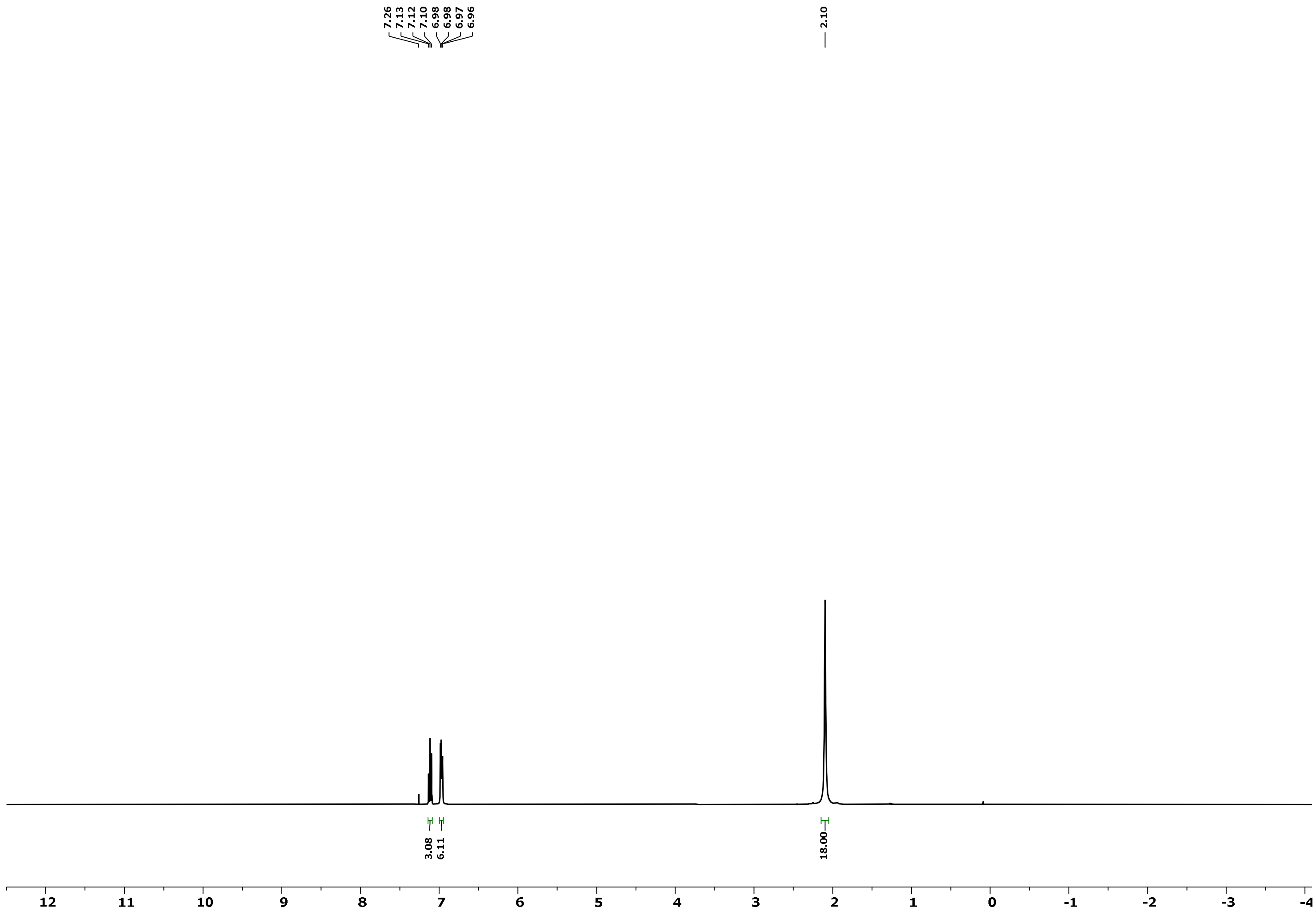

Figure S5.17: ^{13}**C NMR** (101 MHz, $CDCl_3$, 298 K) spectrum of tri(2,6-xyl)phosphine (LB2)

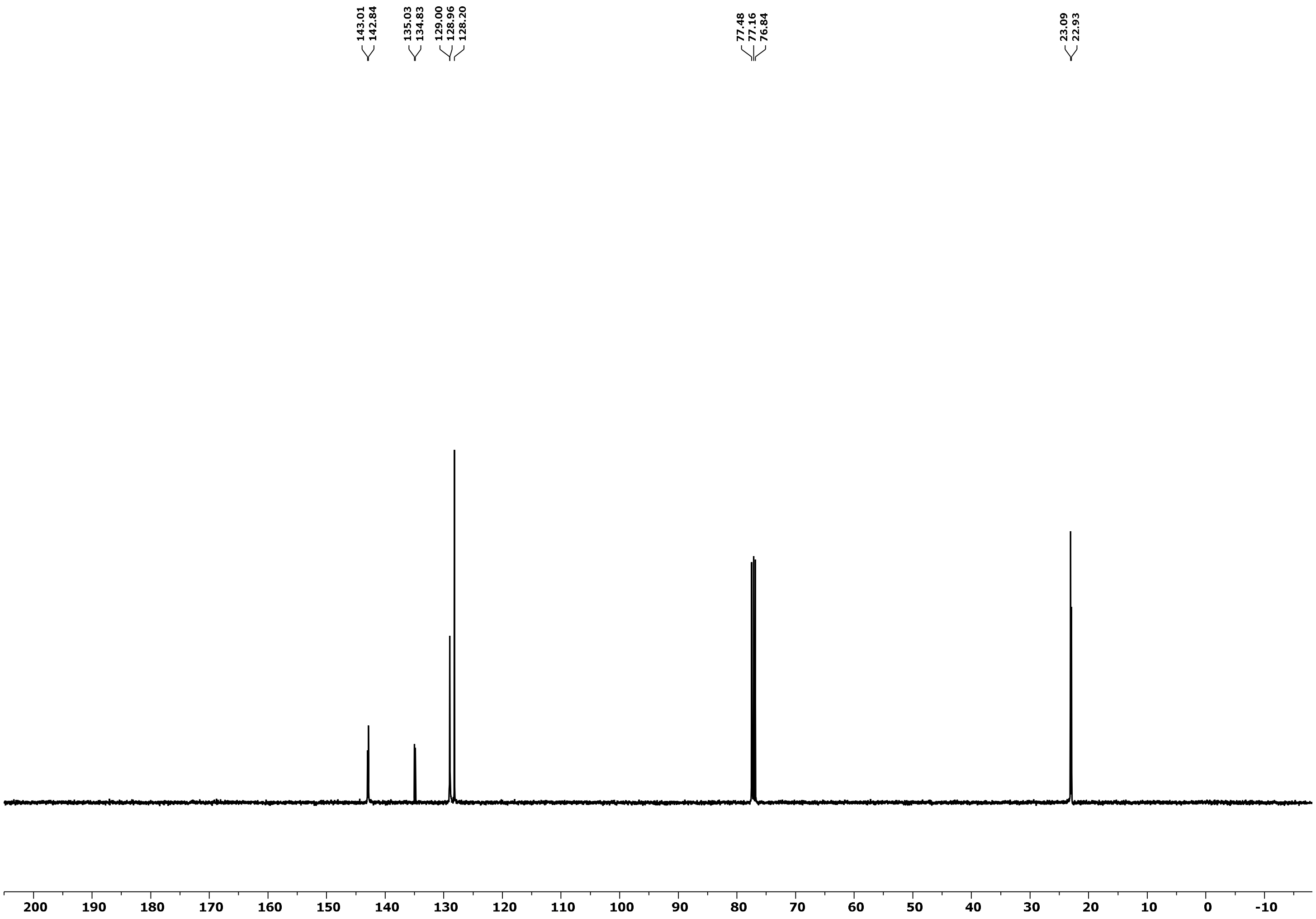

Figure S5.18:**^{31}P NMR** (162MHZ, $CDCl_3$, 298 K) spectrum of tri(o-toyl)phosphine (LB2)

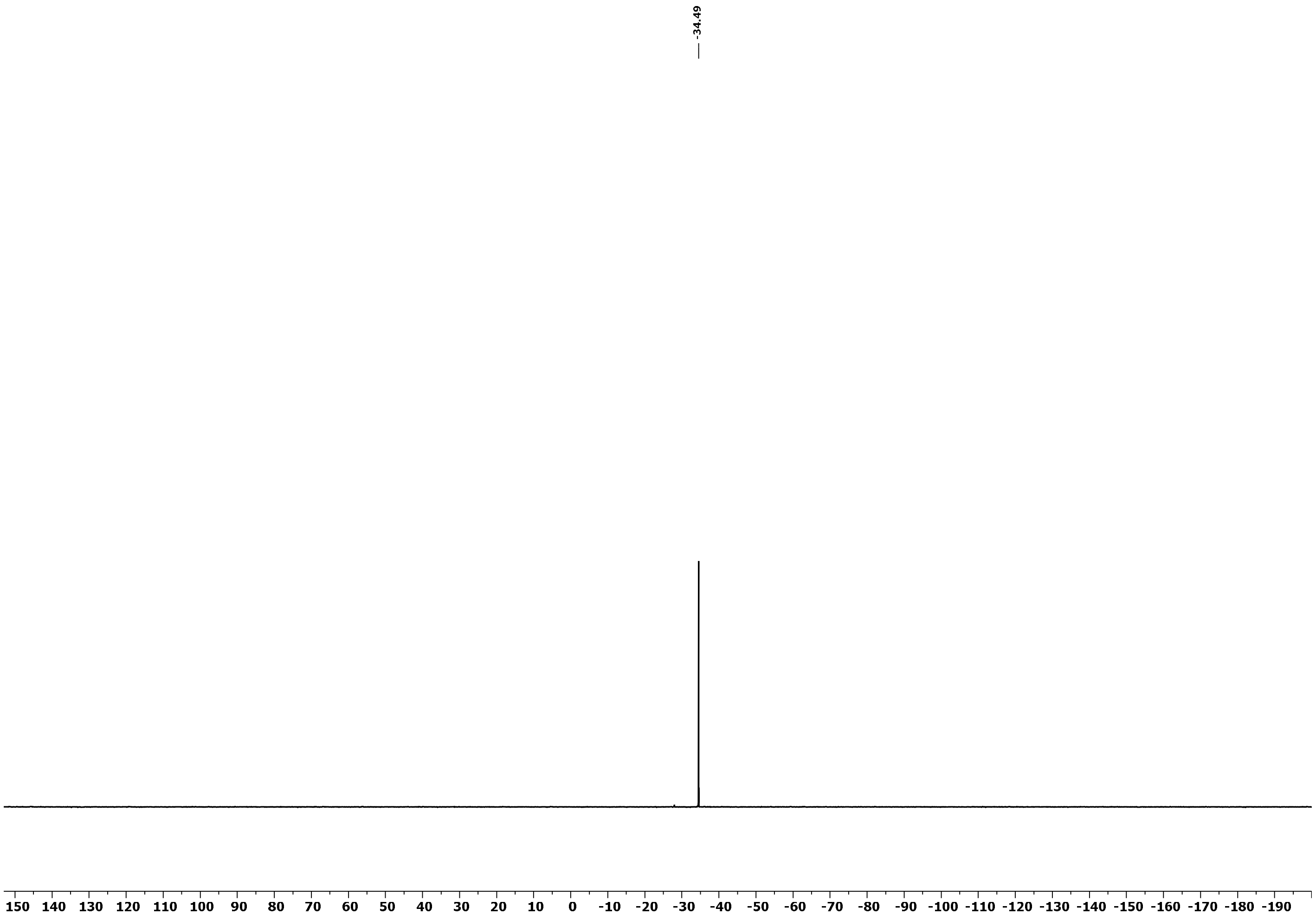

Figure S5.19: ^{1}**H NMR** (400 MHz, $CDCl_3$, 298 K) spectrum of Trimesitylphosphine (LB3)

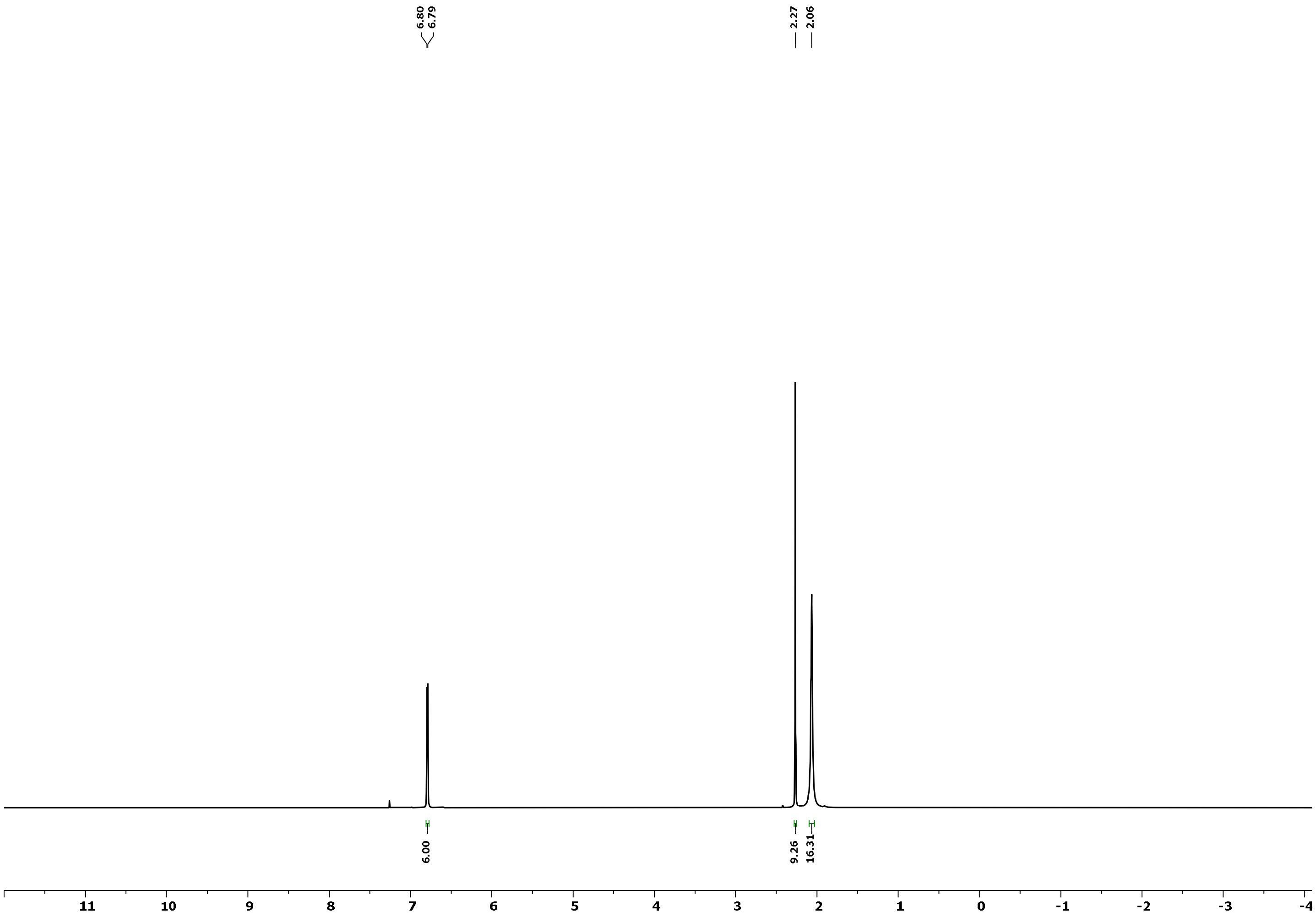

Figure S5.20: ^{13}**C NMR** (101 MHz, $CDCl_3$, 298 K) spectrum of Trimesitylphosphine (LB3)

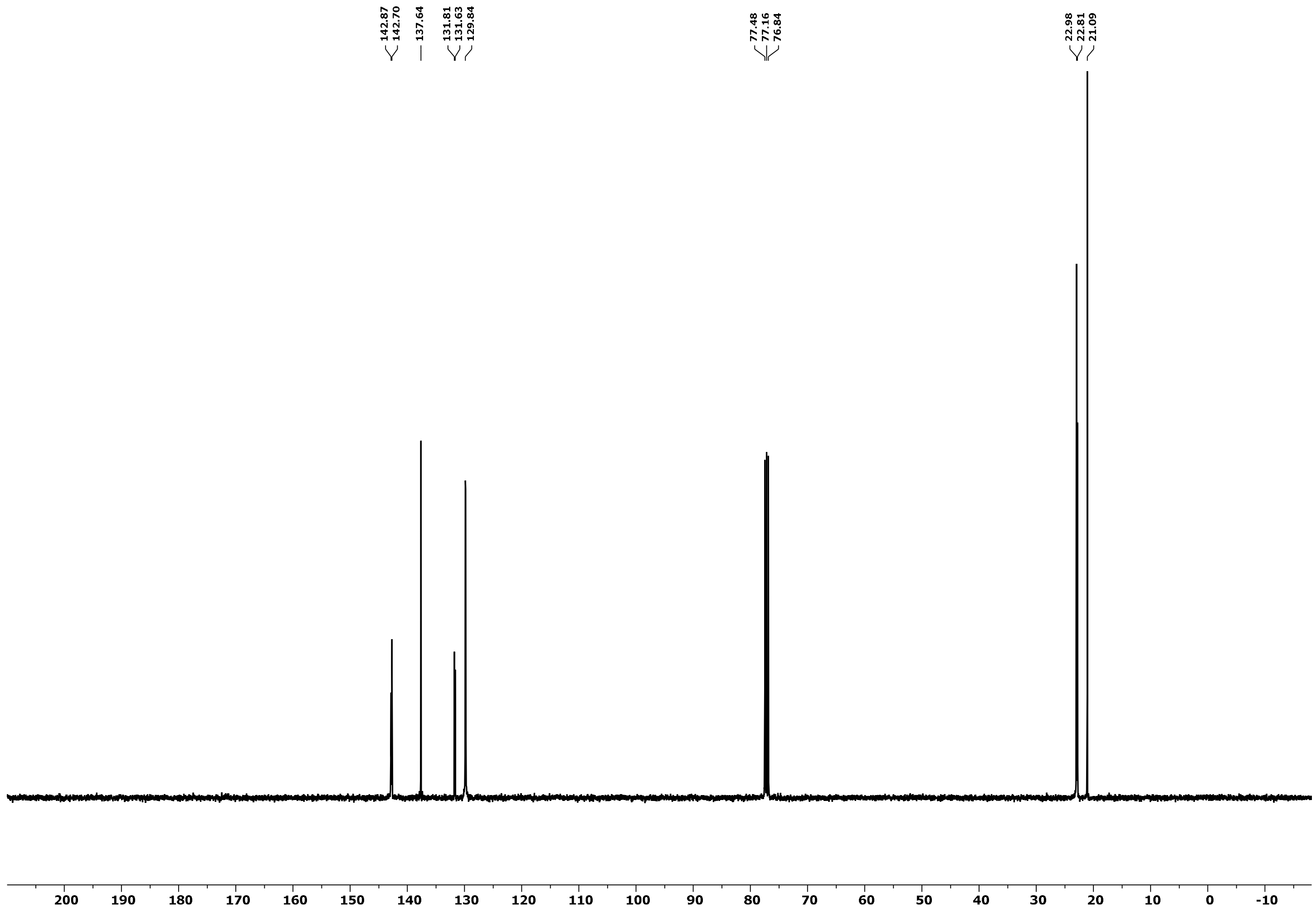

Figure S21: ^{31}**P NMR** (162 MHz, CDCl3, 298 K) spectrum of Trimesitylphosphine(LB3)

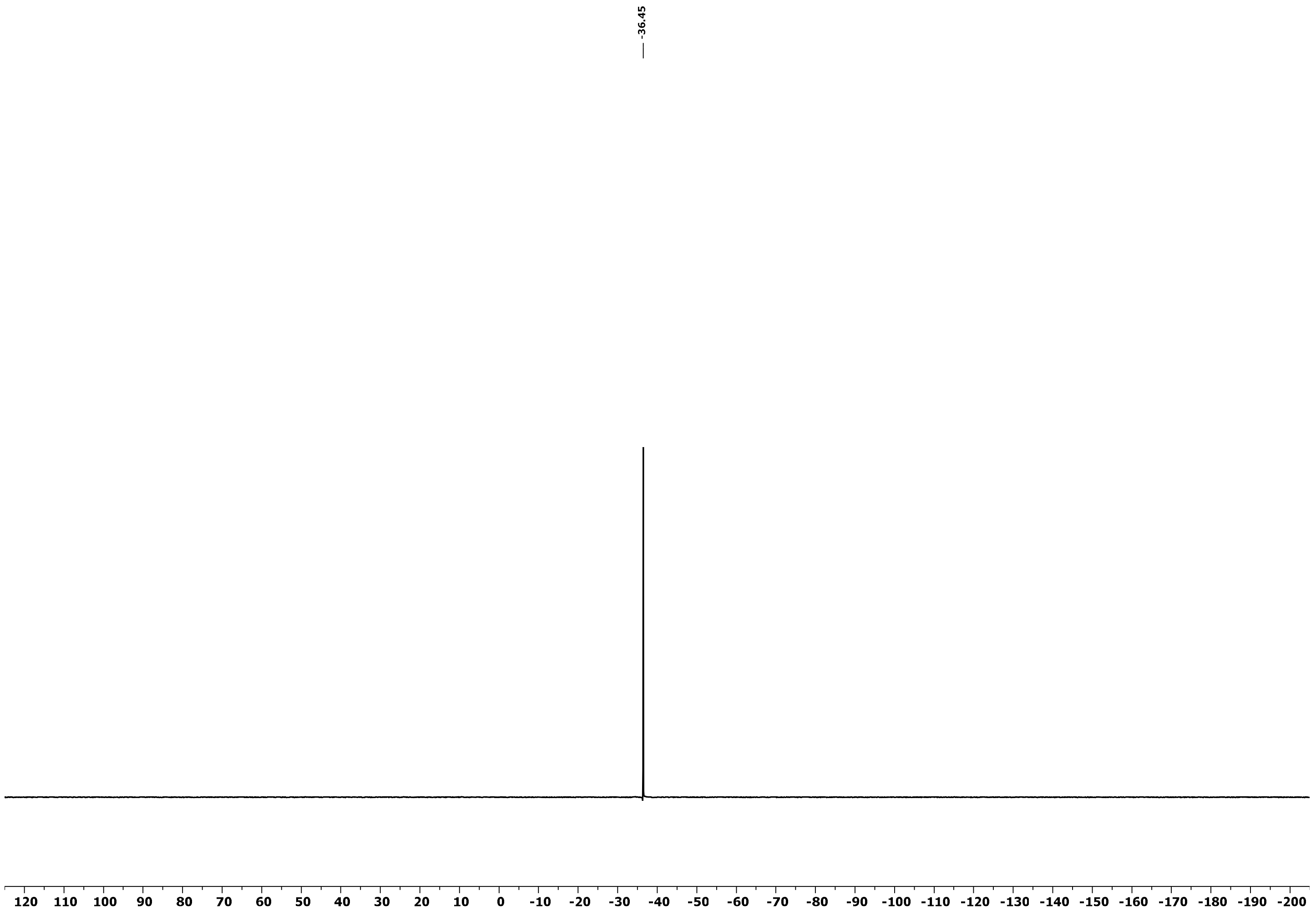

Figure S5.22:[1]**H NMR** (400 MHz, $CDCl_3$, 298 K) spectrum of diethyl ether (LB4)

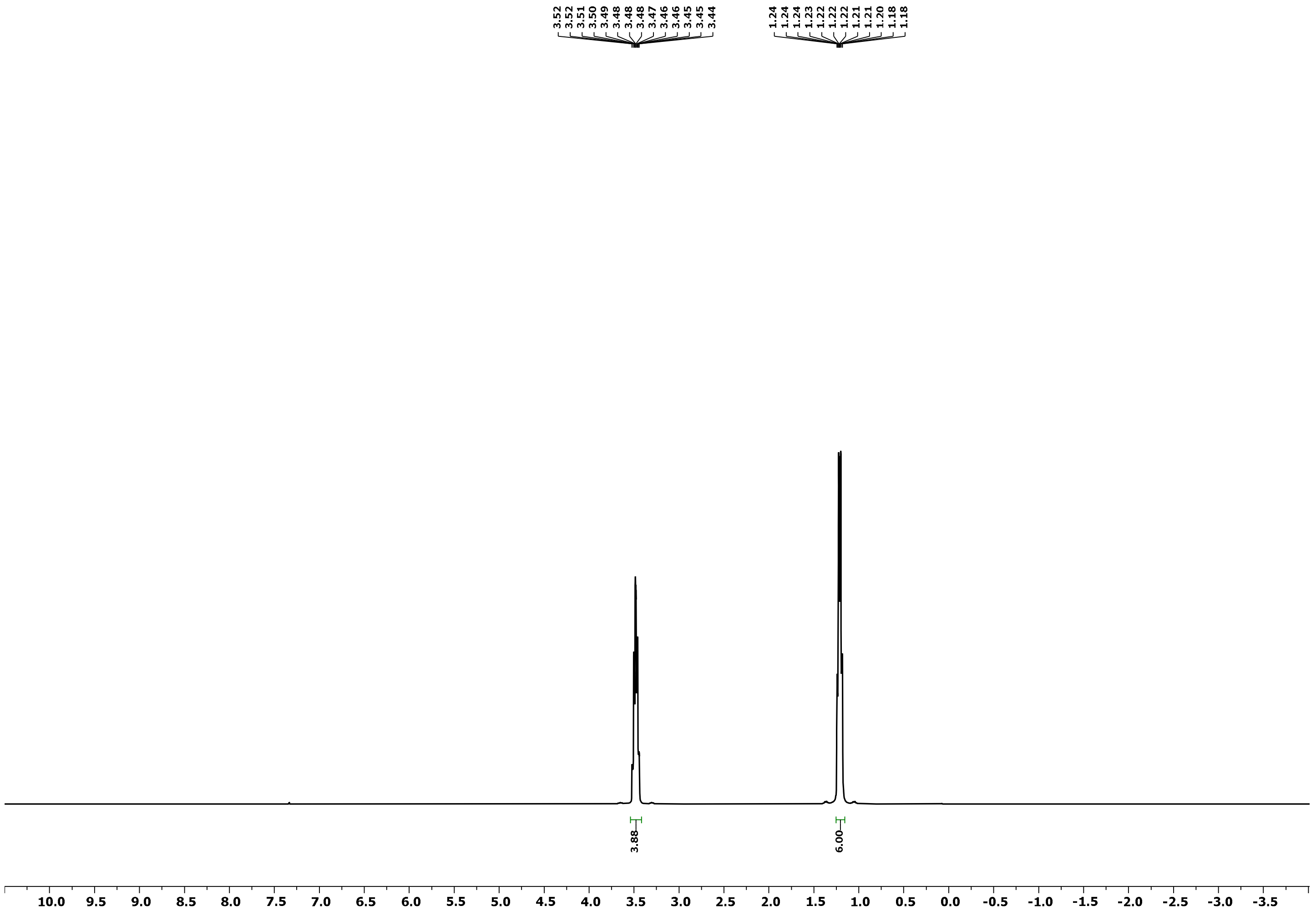

Figure S5.23: ^{13}C **NMR** (101 MHz, $CDCl_3$, 298 K) spectrum of diethyl ether (LB4)

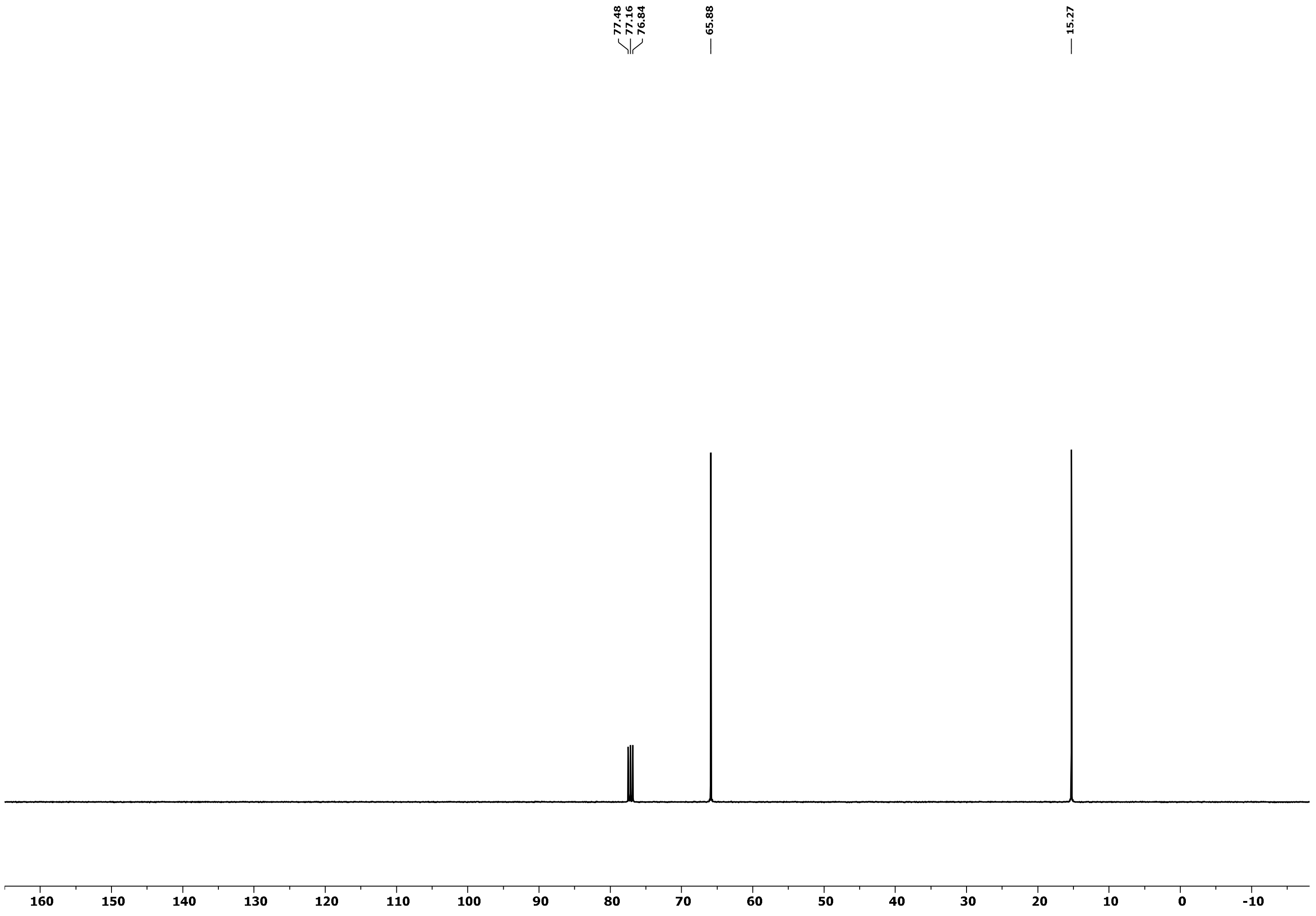

Figure S5.24: ^{1}**H NMR** (400 MHz, $CDCl_3$, 298 K) spectrum of Cyclopentyl methyl ether (LB5)

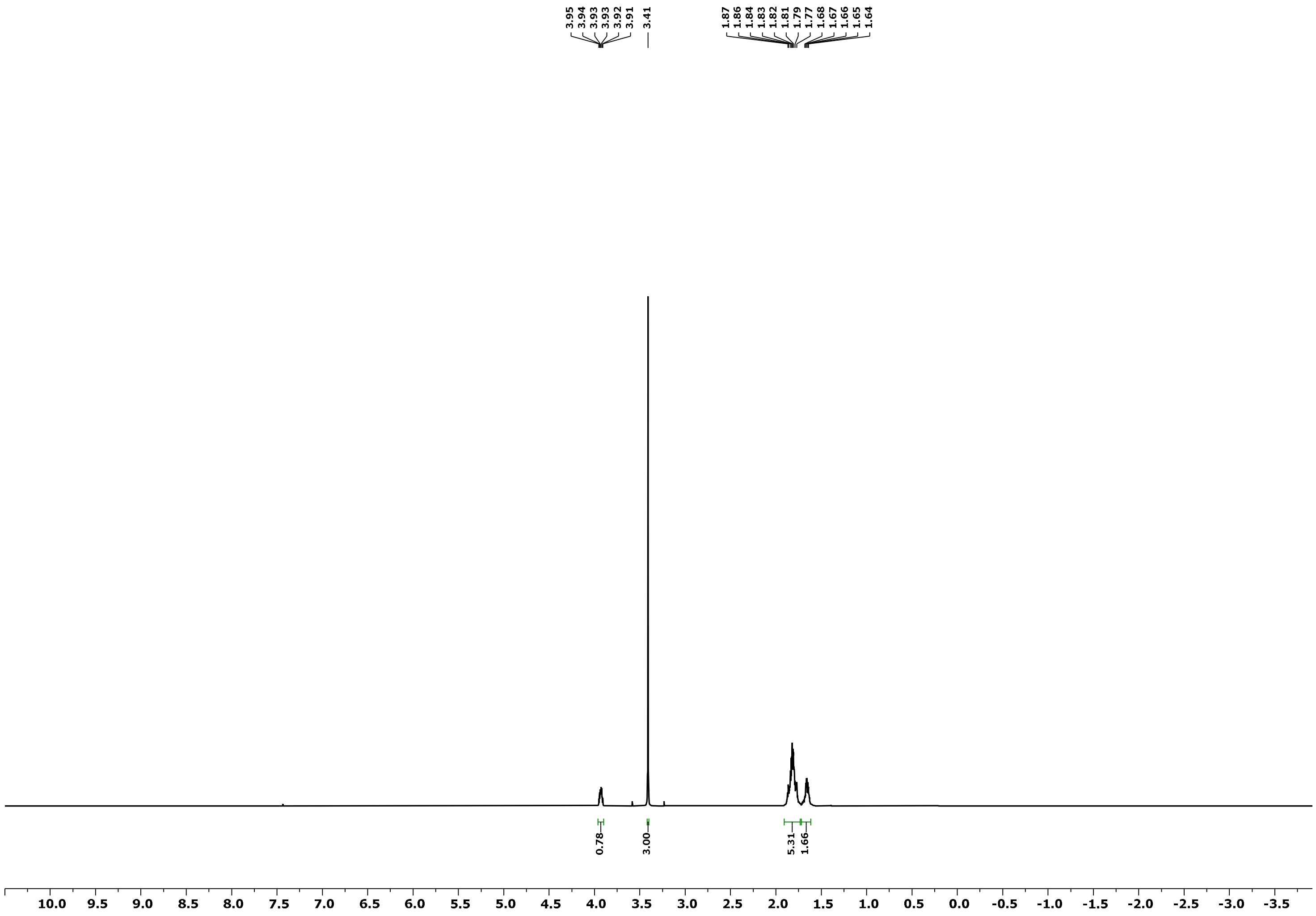

Figure S5.25: ^{13}**C NMR** (101 MHz, $CDCl_3$, 298 K) spectrum of Cyclopentyl methyl ether (LB5)

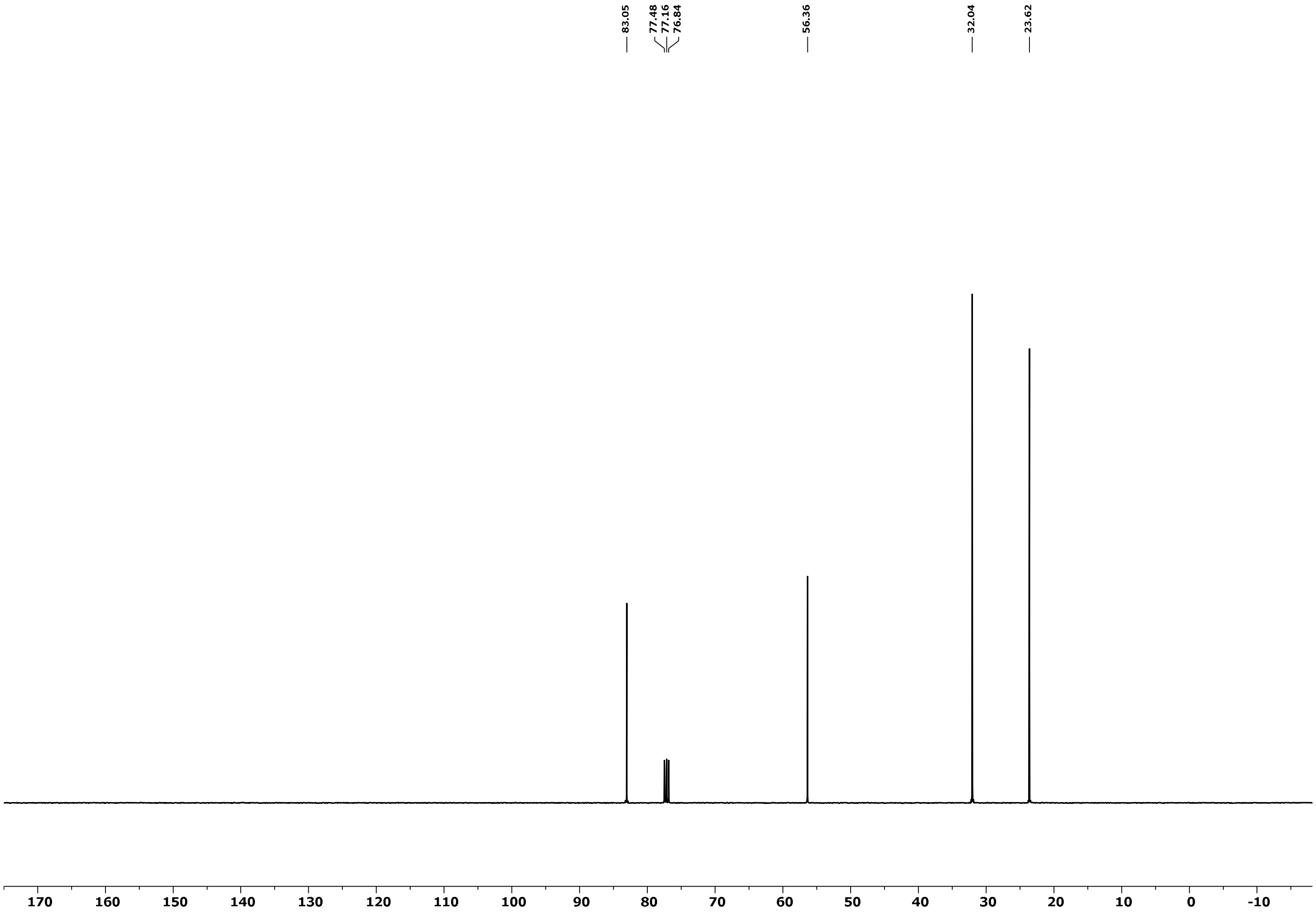

Figure S5.26:**^{1}H NMR** (400 MHz, $CDCl_3$, 298 K) spectrum of methyl tert-butyl ether (LB6)

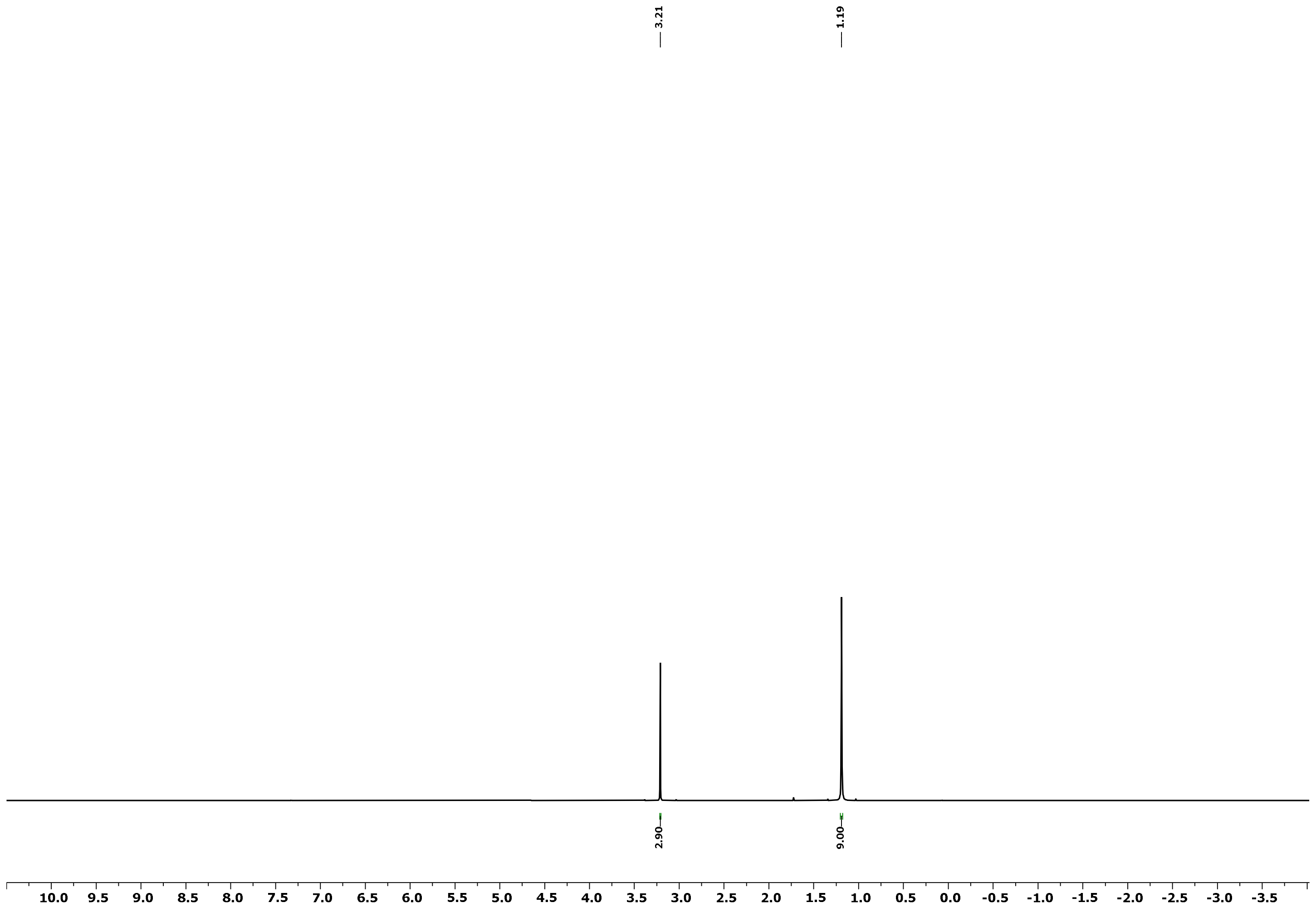

Figure S5.27: ^{13}C NMR (101 MHz, $CDCl_3$, 298 K) spectrum of methyl tert-butyl ether (LB6)

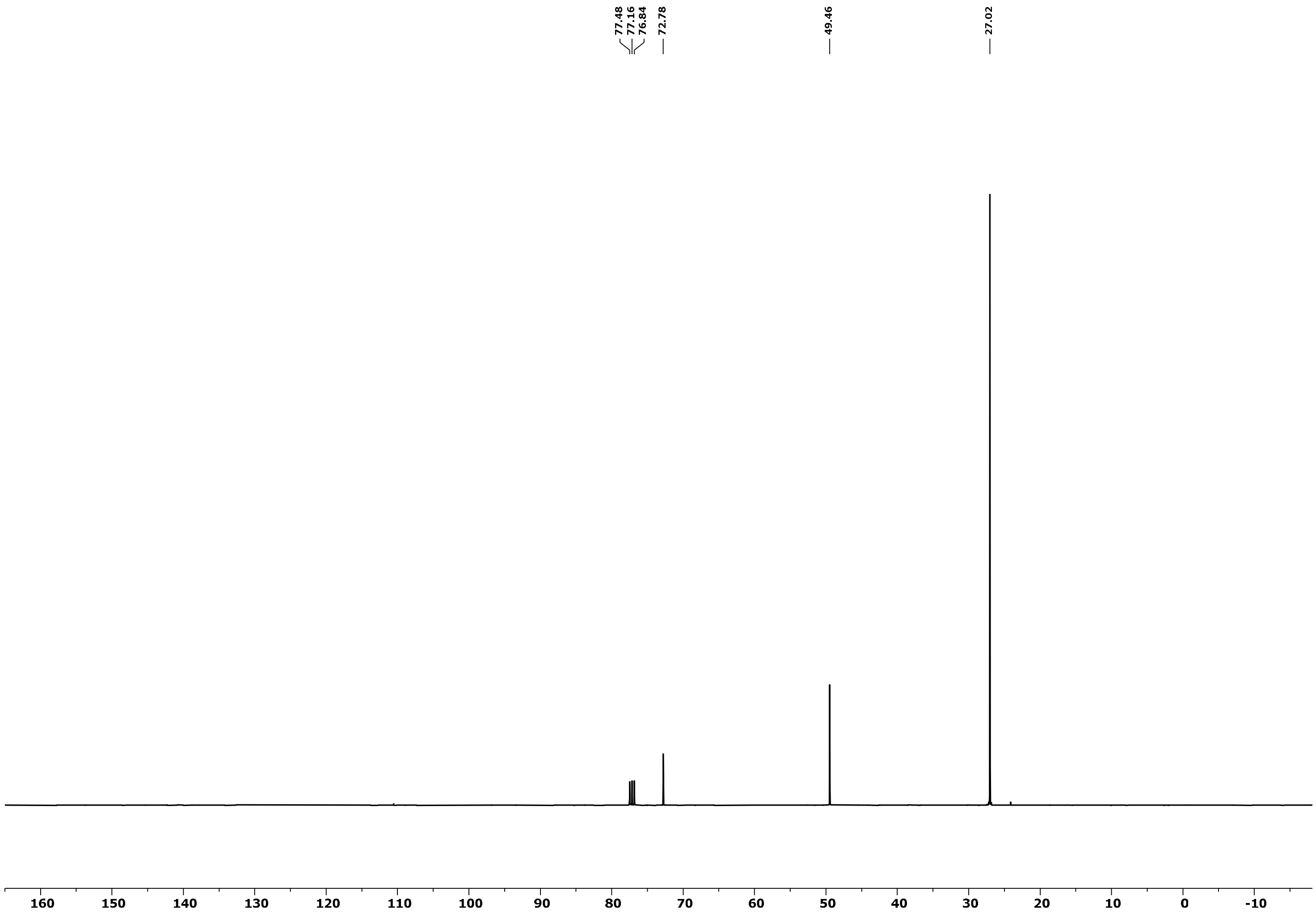

Figure S5.28:^{1}**H NMR** (400 MHz, CDCl3, 298 K) spectrum of eucalyptol **(LB7)**

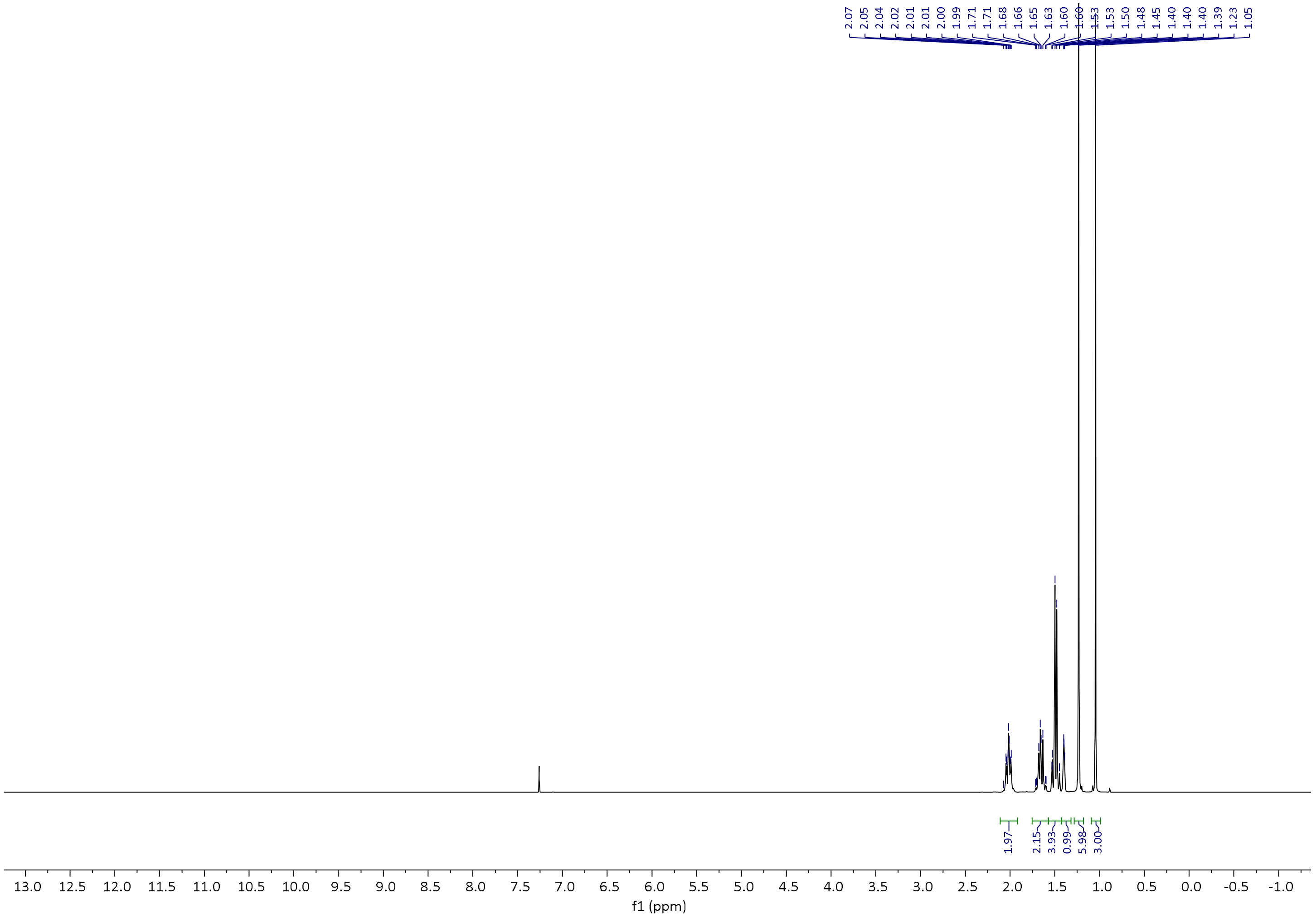

Figure S5.29: ^{13}**C NMR** (101 MHz, $CDCl_3$, 298 K) spectrum of tri(2,6-xyl)phosphine (LB7)

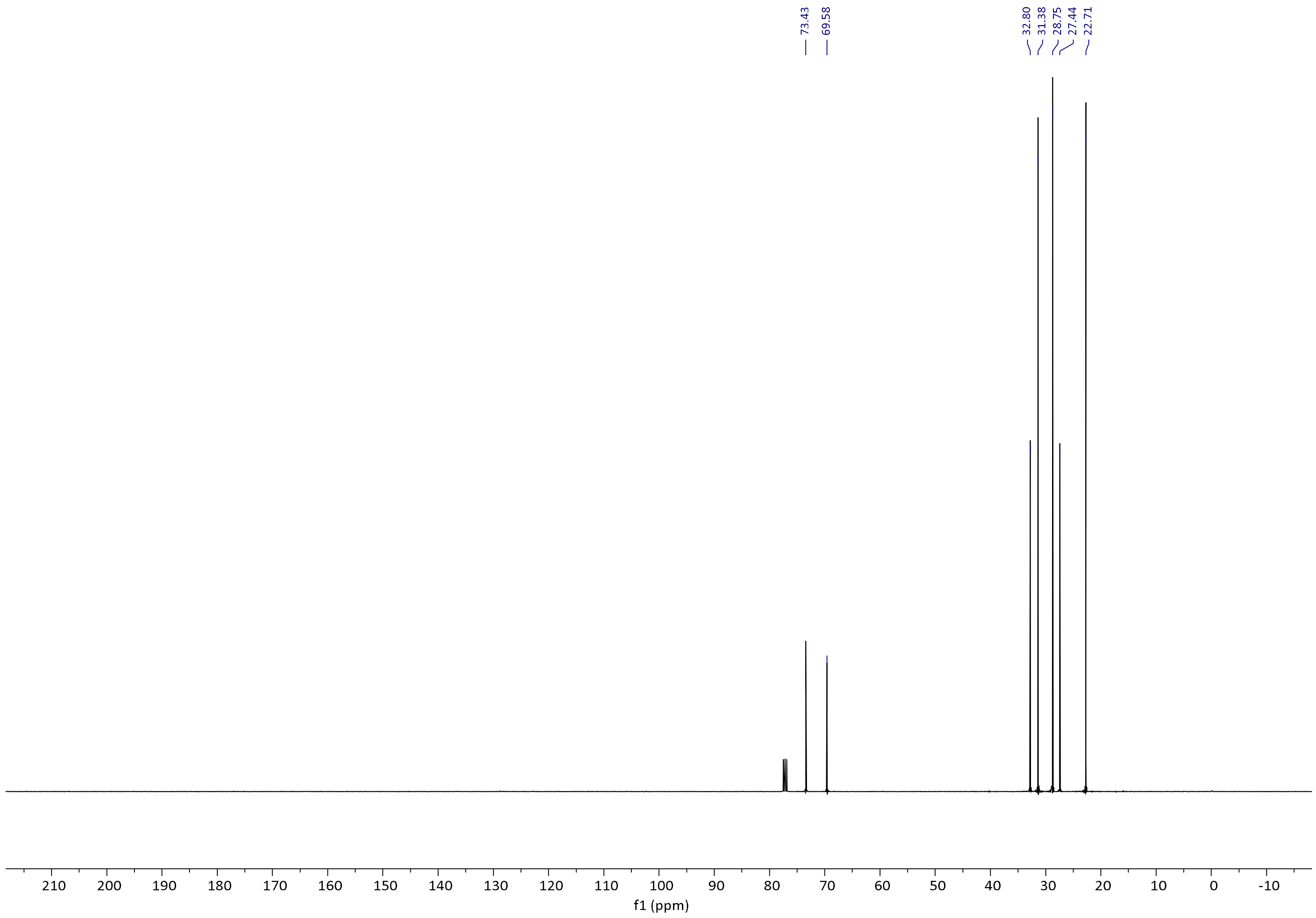

S6 Reference

1. Wang, M.; Nudelman, F.; Matthes, R. R.; Shaver, M. P., Frustrated Lewis Pair Polymers as Responsive Self-Healing Gels. *J. Am. Chem. Soc.* **2017,** *139* (40), 14232-14236.

2. Yolsal, U.; Wang, M.; Royer, J. R.; Shaver, M. P., Rheological Characterization of Polymeric Frustrated Lewis Pair Networks. *Macromolecules* **2019,** *52* (9), 3417-3425.

3. Roesler, R.; Har, B. J. N.; Piers, W. E., Synthesis and Characterization of (Perfluoroaryl)boraneFunctionalized Carbosilane Dendrimers and Their Use as Lewis Acid Catalysts for the Hydrosilation of Acetophenone. *Organometallics* **2002,** *21* (21), 4300-4302.

4. Sivaev, I. B.; Bregadze, V. I., Lewis acidity of boron compounds. *Coord. Chem. Rev.* **2014,** *270*, 75-88.

5. Behrends, I.; Bähr, S.; Czekelius, C., Perfluoroalkylation of Alkenes by Frustrated Lewis Pairs. Chemistry – A European Journal **2016**, 22 (48), 17177-17181.

6. Trunk,M., Teichert,J. F. A. Thomas, *J. Am. Chem. Soc.,* **2017**, 139,3615

7. Cuenca J.A. et al., "Study of the magnetite to maghemite transition using microwave permittivity and permeability measurements", *J. Phys.: Condens. Matter*, vol. 28, art. no.106002, 2015.

8. Santarelli, V.A., MacDonald J.A. and Pine,C. "Overlapping Dielectric Dispersions in Toluene", J. Chem. Phys. vol. 46, no. 6, pp.2367-2375, Mar. 1967.

9. Los Alamos National Laboratory Report LA-UR-87-115, Superfish group of codes.
